# Design of Highly Conductive PILs by Simple Modification of Poly(epichlorohydrin-*co*-ethylene oxide) with Monosubstituted Imidazoles

**DOI:** 10.1021/acspolymersau.4c00051

**Published:** 2024-09-12

**Authors:** Daniil R. Nosov, Elena I. Lozinskaya, Dmitrii Y. Antonov, Denis O. Ponkratov, Andrey A. Tyutyunov, Malak Alaa Eddine, Cédric Plesse, Daniel F. Schmidt, Alexander S. Shaplov

**Affiliations:** † Luxembourg Institute of Science and Technology, 5 Avenue des Hauts-Fourneaux, L-4362 Esch-sur-Alzette, Luxembourg; ‡ Department of Physics and Materials Science, University of Luxembourg, 2 Avenue de l’Université, L-4365 Esch-sur-Alzette, Luxembourg; § A.N. Nesmeyanov Institute of Organoelement Compounds Russian Academy of Sciences (INEOS RAS), Vavilov Street 28, Bld. 1, 119334 Moscow, Russia; ∥ Univ Lyon, Université Lyon 1, CNRS, Ingénierie des Matériaux Polymères, UMR 5223, F-69003 Lyon, France; ⊥ CY Cergy Paris Université, Laboratoire de Physicochimie des Polymères et des Interfaces, 5 Mail Gay Lussac, F-95031 Cergy-Pontoise Cedex, France

**Keywords:** poly(ionic liquid)s, polyelectrolyte, ionic conductivity, conductive materials, poly(epichlorohydrin-*co*-ethylene oxide)

## Abstract

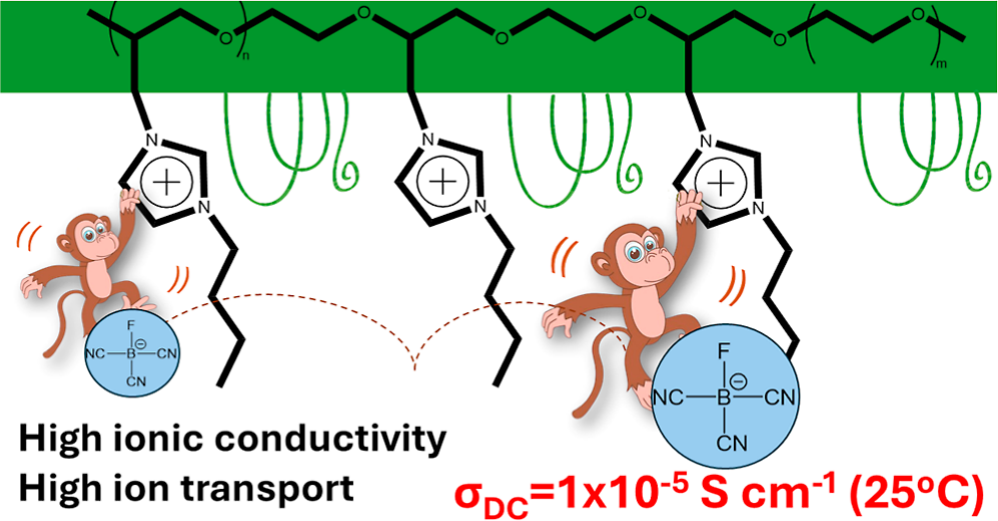

High ionic conductivity poly(ionic liquid)s (PILs) are of growing interest for their thermal and electrochemical stability, processability, and potential in safe, flexible all-solid-state electrochemical devices. While various approaches to enhance the ionic conductivity are reported, the influence of cation substituents is rarely addressed. Moreover, some of the asymmetric anions recently developed for high-conductivity ionic liquids were never tested in PILs. We report the design and synthesis of twelve novel cationic PILs prepared via quaternization of N-substituted imidazoles by commercially available poly(epichlorohydrin-*co*-ethylene oxide) (poly(EPCH-*r*-EO)) with subsequent ion metathesis. They differ by imidazolium side chain length (C_1_–C_6_ alkyl) and presence of heteroatoms (silyl, siloxane, and fluoroalkyl) and by anion type (bis(trifluoromethylsulfonyl)imide (TFSI), 2,2,2-trifluoromethylsulfonyl-*N*-cyanoamide (TFSAM), tetrafluoroborate (BF_4_), trifluoro(trifluoromethyl)borate (BF_3_CF_3_), and tricyanofluoroborate (BF(CN)_3_)). TFSI-based PILs with alkyl side chains gave lower glass transition temperatures (*T*_g_) and higher ionic conductivities than those bearing heteroatomic substituents, with *n*-butyl side chains providing a conductivity of 4.7 × 10^–6^ S cm^–1^ at 25 °C under anhydrous conditions. This increased to 1.0 × 10^–5^ and 4.5 × 10^–4^ S cm^–1^ at 25 and 70 °C, respectively, when the TFSI anion was replaced with BF(CN)_3_. All PILs showed good electrochemical (>3.2 V vs Ag^+^/Ag) and thermal (>185 °C) stability, making them excellent candidates for solid-state electrolytes in electrochemical devices.

## 1 Introduction

Nowadays, poly(ionic liquids) (PILs) have been successfully applied as solid ion conducting materials in various electrochemical devices such as supercapacitors, Li-ion batteries, solar cells, artificial muscles, etc.^1−5^ However, both the working temperature and the operational regime of such electrochemical devices were significantly dependent on the bulk ionic conductivity (σ_DC_) of PIL, thus limiting the practical application of the later. In consequence, the development of novel PILs with improved ionic conductivity is of high importance and represents the subject of competition between various scientific groups.^1,6−9^

**Scheme 1:**
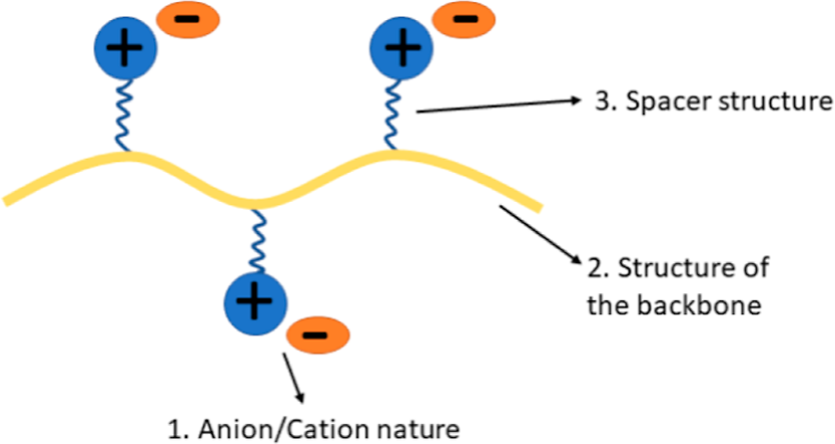
Schematic Illustration of PILs and Structural Factors Affecting Their Ionic Conductivity

The design of new PILs (Scheme 1) can be based on tuning the following parameters that are known to influence the bulk ionic conductivity of a polyelectrolyte: the anion and cation nature (1), the architecture of the polymer backbone (2), and the length and nature of the spacer (3) between the chemically bonded ion and main polymer chain.^1,3^ The influence of each of these parameters on the ionic conductivity of PILs will be discussed in detail below.

### 1.1 Type of Organic Cation

**Scheme 2:**
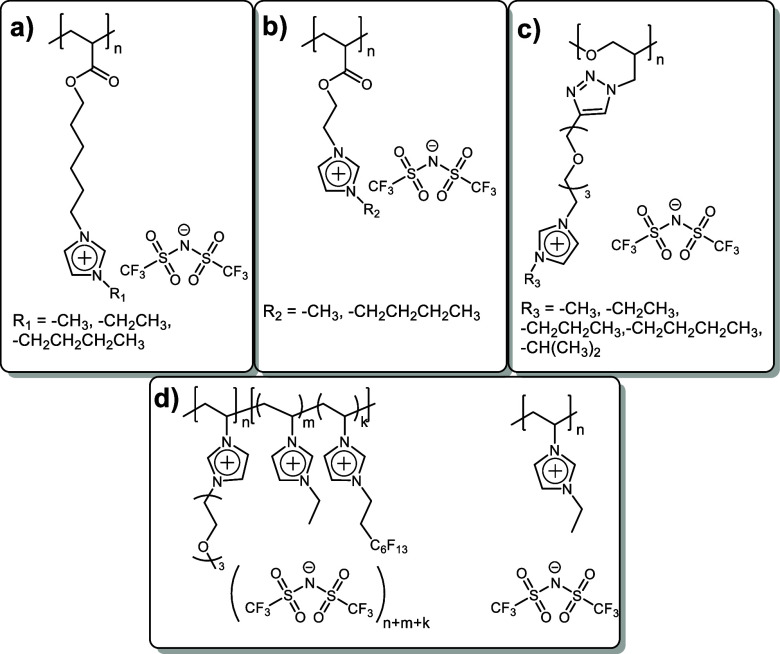
Examples of PILs Illustrating the Influence of the Substituent in the Cation on Bulk Polymer Ionic Conductivity

Cation structure, in particular its type and the substituents, showed a strong influence on the ionic conductivity of PILs.^1^ Among the vast amount of cations, polyelectrolytes with positively charged imidazolium heterocycles demonstrated the best performance in terms of conductivity and may be considered as the most promising candidates for further investigation.^10,11^ At the same time, the study of substitutes influences or side chains in imidazolium ring on PIL’s bulk ionic conductivity was mainly limited to the negligible variation in length^10−13^ and isomerism^13^ of hydrocarbon chains (Scheme 2a–c). In spite of the similarity in PIL structures, the results obtained by several research groups were contradictory. Thus, Ohno et al.^10^ revealed the extremal character of ionic conductivity in bis(trifluoromethylsulfonyl)imide (TFSI)-based PILs during the transfer of methyl to *n*-butyl substitute in imidazolium cation (Scheme 2a):

σ (25 °C, S cm^–1^): –CH_3_ (4.4 × 10^–5^) < –CH_2_CH_3_ (1.4 × 10^–4^) > –(CH_2_)_3_–CH_3_ (4.1 × 10^–5^).

On the contrary, in a very similar imidazolium PILs, the increase in the substituent length from methyl^11^ to *n*-butyl^12^ group led to the linear growth in ionic conductivity from 7.4 × 10^–10^ to 8.5 × 10^–7^ S cm^–1^ at 25 °C (Scheme 2b). Another trend was observed in the recent publication of Ikeda et al.,^13^ where the conductivity of PILs was first increasing by an half order of magnitude with the transfer from methyl to ethyl substitute and then became constant independently of the substitute length (Scheme 2c):

σ (30 °C, S cm^–1^): –CH_3_ (5.0 × 10^–6^) < –CH_2_CH_3_ (1.5 × 10^–5^) ≈ –(CH_2_)_2_–CH_3_ (1.3 × 10^–5^) ≈ –CH–(CH_3_)_2_ (1.1 × 10^–5^) ≈ –(CH_2_)_3_–CH_3_ (1.5 × 10^–5^)

At this point, the influence of imidazolium substituents containing heteroatoms (Si, F, O, etc.) on the ionic conductivity of PILs practically was not studied. Although some reports discussing such PILs appeared recently,^14−16^ they were mainly dedicated to the elaboration of the synthetic pathways for the preparation of imidazolium PILs with fluorinated, silyl, or siloxane substituents, while the conductivity of the resultant polymers was not measured. To the best knowledge of the authors, only one study by Detrembleur and Drockenmuller et al.^17^ can show the indirect comparison of the influence of fluorinated substituents on the conductivity of imidazolium PILs (Scheme 2d). The introduction of the side fluorinated chain along with triethylene glycol pendant groups in imidazolium cations resulted in higher ionic conductivity in comparison with the homopolymer analogue bearing the hydrocarbon substitute (3.0 × 10^–7^ and 2.5 × 10^–11^ S cm^–1^ (30 °C), respectively).^1^ However, the observed increase in overall conductivity may also be related solely to the presence of triethylene glycol pendant groups in the third block, known to promote the conductivity of PILs.^18^

### 1.2 Polymer Backbone

**Scheme 3:**
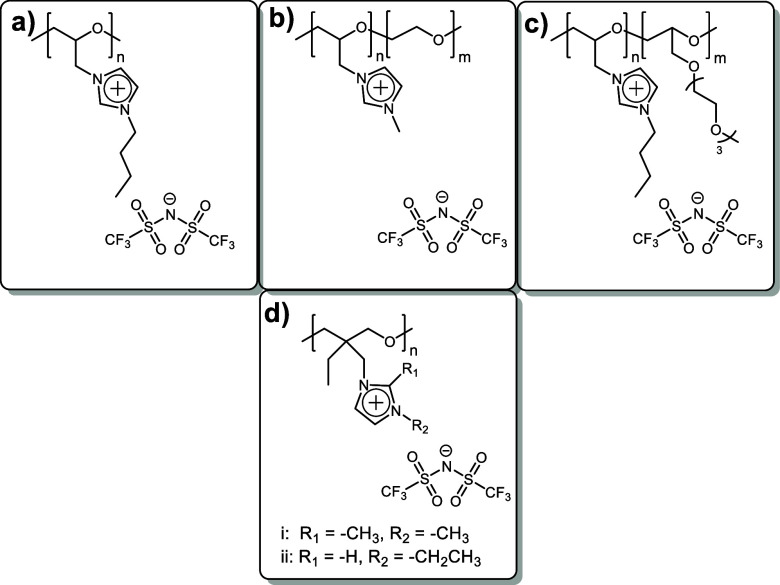
Examples of PILs with Alkylene Oxide Main Chains

The polymer backbone is another important factor to consider when designing highly conductive PILs.^1,4,6,8,19−21^ The direct comparison of conductivity in PILs having various backbones such as methacrylate, acrylate, siloxane, norbornene, etc. (see Table S1 as an example) represents a known problem as it is quite hard to find examples of PILs with similar cations, anions, and spacers, although they differ only by the nature of the main chain. However, it is possible to postulate some common trends. First, the main chain of a PIL should be flexible, as the ionic conductivity crucially depends on the PIL’s glass transition temperature (*T*_g_). As a rule of thumb, polyelectrolytes with lower *T*_g_ show higher ionic conductivity.^1,4,21,22^ It is important to note that this rule is only valid if the difference between the glass transition temperature and the temperature at which the ionic conductivity is measured does not exceed 30–35 °C.^23^ Second, the presence of alkylene oxide fragments was found to be beneficial for the conductivity of PILs as they promote ion solvation and as a result increase the ion mobility and ionic conductivity.^18,24,25^ Thus, PILs with flexible poly(ethylene oxide) (Scheme 3a–c) or poly(propylene oxide) (Scheme 3d) backbones were capable of showing significantly high ionic conductivities. Baker et al.^26^ prepared a set of polyethylene oxide PILs with the molecular weights ranging from 21,000 to 76,000 g mol^–1^ via ring opening polymerization of epichlorhydrin and subsequent polymer quaternization. These oligomeric PILs demonstrated relatively high ionic conductivity above 1 × 10^–6^ S cm^–1^ (Scheme 3a). Further on, Shaplov et al.^27^ suggested to quaternize poly(epichlorohydrin-*co*-ethylene oxide) copolymer having a high molar mass of 8.7 × 10^6^ g mol^–1^ with *N*-methyl imidazole (Scheme 3b). This approach allowed to “dilute” the charge careers with additional ethylene oxide (EO) units, helping their dissociation and providing the ionic conductivity up to 8.4 × 10^–7^ S cm^–1^ at 25 °C and simultaneously to improve the mechanical properties of PILs due to their high molecular weight of the precursor (Scheme 3b). Later on, Baker et al.^28^ proposed to copolymerize epichlorohydrin with 2-((2-(2-(2-methoxyethoxy)ethoxy)ethoxy)methyl) oxirane in different ratios. The quaternization of obtained copolymers with *N*-butyl imidazole and subsequent ion metathesis provided PILs with ionic conductivities up to 1.2 × 10^–4^ S cm^–1^ (25 °C) at an equimolar ratio of comonomers (*n*/*m* = 1:1). Such an improvement in conductivity can be explained by the introduction of EO containing side so-called “dangling” chains, additional “dilution” of charges with EO units, and relatively low *M*_n_ (2.2 × 10^3^ g mol^–1^). Finally, PILs having the poly(propylene oxide) backbone (Scheme 3d) were synthesized by Matsumoto et al.^29^ via ring opening polymerization of ionic monomers with four-membered cyclic ether oxetanyl moieties. These PILs showed only moderate ionic conductivity of 2.0 × 10^–8^ S cm^–1^ (25 °C) that can be explained by analogy with the difference in conductivity between polymer electrolytes based on poly(ethylene oxide) and poly(propylene oxide) filled with Li salts:^30,31^ the presence of the ethyl groups prevents coordination of anions with oxygen, thus disrupting the hopping mechanism of ion mobility.

### 1.3 Type of Anion

**Scheme 4:**
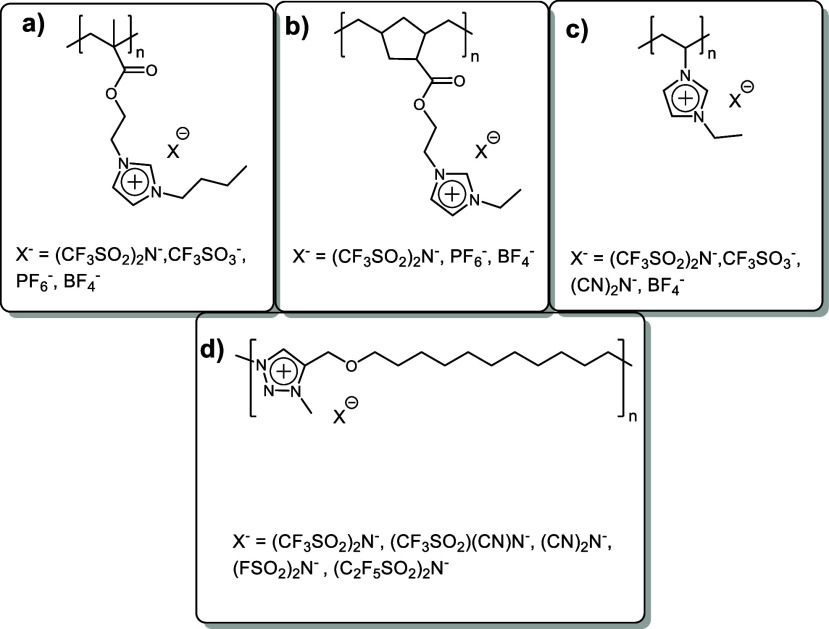
Examples of PILs Illustrating the Influence of the Anion Structure on Bulk Polymer Ionic Conductivity

Lastly, the anion structure also shows great impact on PIL properties: the reduction in size and increase in charge delocalization are the most powerful tools for the improvement of PIL ionic conductivity.^23^ Although the direct influence of anion structure on ionic conductivity of PILs can be found in a variety of published works,^12,32−38^ the majority of them were dedicated to the study of only four anions, namely TFSI, PF_6_, BF_4_, and CF_3_SO_3_. For example, for poly(1-[(2-methacryloyloxy)ethyl]-3-butylimidazolium)s (Scheme 4a), the following order of conductivity values with respect to chemical structure of the counteranions was found:

σ (30 °C, S cm^–1^): (CF_3_SO_2_)_2_N (4.0 × 10^–4^) > CF_3_SO_3_ (1.5 × 10^–5^) > BF_4_ (6.5 × 10^–6^) > PF_6_ (3.8 × 10^–6^).

In the work of Buchmeiser et al.^37^ a series of polynorbornene derivatives with different anions were synthesized and investigated. Similarly, the transition from PF_6_ to BF_4_ and further to more delocalized TFSI anion led to the increase in ionic conductivity:

σ (25 °C, S cm^–1^): (CF_3_SO_2_)_2_N (4.2 × 10^–7^) > BF_4_ (9.8 × 10^–9^) > PF_6_ (3.8 × 10^–11^).

In a number of polyvinyl imidazoles, the significant increase in conductivity was achieved by the application of a small delocalized dicyanamide anion.^35^ At the same time, PIL bearing the TFSI anion demonstrated only moderate conductivity increase^35^ in comparison with BF_4_ and CF_3_SO_3_ anions.

σ (25 °C, S cm^–1^): (CN)_2_N (1.4 × 10^–5^) ≫ (CF_3_SO_2_)_2_N (2.5 × 10^–11^) > CF_3_SO_3_ (4.9 × 10^–12^) > BF_4_ (<10^–12^).

Starting in the 2000s, the introduction of the asymmetry principle in anions structure has become a very successful approach for the synthesis of ionic liquids with the lowest melting points and viscosities and, as a result, with the highest known ionic conductivity.^39,40^ However, such novel anions were practically not tested with PILs, and to the best of our knowledge, only one report describing the comparison of PILs with asymmetric anions exists to date.^32^ In this paper, Shaplov and Drockenmuller synthesized a series of PILs having a triazolium cation and five different anions. PILs under investigation demonstrated nearly similar ionic conductivities with TFSI-based PILs outmatching those bearing the asymmetric TFSAM anion by half an order of magnitude. The observed conductivity trend at 30 °C can be represented by the raw below:

σ (30 °C, S cm^–1^): (CF_3_SO_2_)_2_N (8.5 × 10^–6^) > (C_2_F_5_SO_2_)_2_N (6.2 × 10^–6^) > (CN)_2_N (5.8 × 10^–6^) > (FSO_2_)_2_N (3.5 × 10^–6^) > (CF_3_SO_2_)(CN)N (1.8 × 10^–6^).

**Scheme 5:**
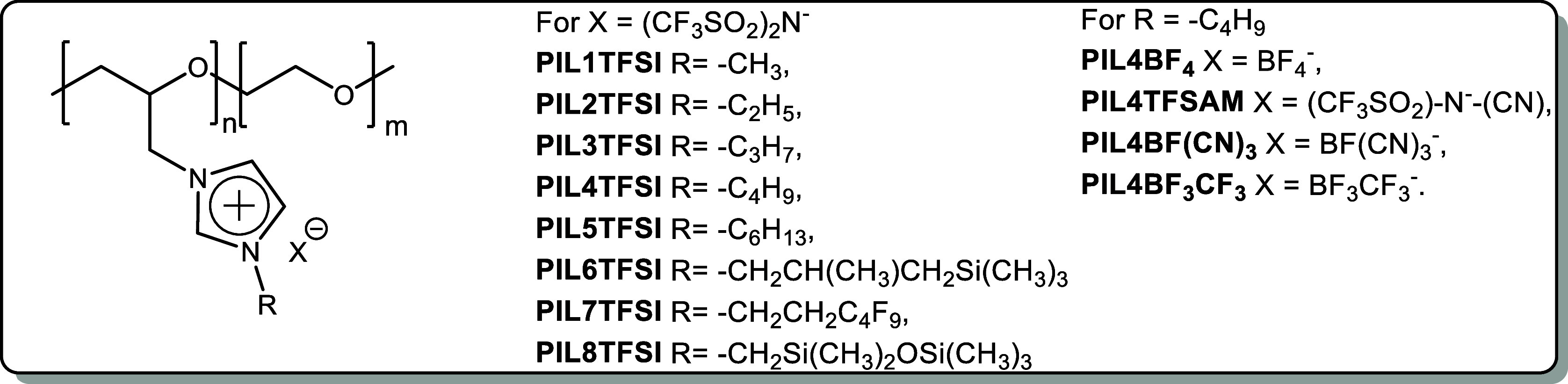
Novel PILs Designed and Synthesized in the Present Work

To address the goal of PIL bulk ionic conductivity improvement, in this work, we suggest a series of novel polyelectrolytes (Scheme 5) designed by taking into account all the principles described above. Poly(epichlorohydrin-*co*-ethylene oxide) (poly(EPCH-*r*-EO), Hydrin) having flexible chain and ethylene oxide fragments along the backbone was selected as a commercially available precursor for the novel PIL formation. The synthetic approach consists of two modification steps, including quaternization reactions of various mono N-substituted imidazoles and ion exchange reactions with metal salts bearing both known TFSI and BF_4_ anions, as well as recently introduced asymmetric 2,2,2-trifluoromethylsulfonyl-*N*-cyanoamide (TFSAM), trifluoro(trifluoromethyl)borate (BF_3_CF_3_), and tricyanofluoroborate (BF(CN)_3_) anions (Scheme 5). Thus, in the presented work, two fundamental factors influencing ionic conductivity in PILs, namely, the nature of the side chains in imidazolium cation and the structure of the anion, were investigated. At first, a set of PILs bearing TFSI anion was synthesized, varying the length of the substituents and the presence of heteroatoms in the side chains (Scheme 5). The highest ionic conductivity of 4.7 × 10^–6^ S cm^–1^ (25 °C) was demonstrated by **PIL4** with *n*-butyl substituted imidazolium cation. This served as the basis for the selection of this particular chloride precursor for ion metathesis with metal salts of new asymmetric anions.

Finally, the introduction of asymmetric anions in the PIL’s structure allowed to increase the ionic conductivity and to reach the value of 1.0 × 10^–5^ S cm^–1^ (25 °C) for PIL bearing the BF(CN)_3_ anion that, to the best knowledge of the authors (Table S1), can be considered among the top 15 conductive PILs published to date.

## 2 Experimental Section

### 2.1 Materials

*N*,*N*-dimethylformamide (DMF, anhydrous 99,8%, Acros), acetone (99.6%, Acros), acetonitrile (MeCN, anhydrous 99.9%, Acros), diethyl ether (anhydrous 99%, Acros), *n*-hexane (95%, Acros), dichloromethane (99.6%, Acros), benzene (Acros), pyridine (99+%, Acros), poly(epichlorohydrin-*co*-ethylene oxide) (Hydrin C2000XL, Zeon Europe GmbH), hydrochloric acid (HCl, 37% in water, Acros), 4-toluenesulfonyl chloride (99%, Aldrich), potassium iodide (KI, 99%, Aldrich), sodium hydrogen carbonate (NaHCO_3,_ Aldrich), potassium carbonate (K_2_CO_3_, 99%, Aldrich), iodomethane (99%, Aldrich), 1-(chloromethyl)-1,1,3,3,3-pentamethyldisiloxane (99%, ABCR), (3-chloro-2-methylpropyl)trimethylsilane (95+%, Gelest), 3,3,4,4,5,5,6,6,6-nonafluoro-1-hexanol (97%, Aldrich), sodium hydride (60 wt % dispersion in mineral oil, Aldrich), imidazole (99%, Aldrich), lithium bis(trifluoromethylsulfonyl)imide (LiTFSI, 99%, Solvionic), and sodium tetrafluoroborate (NaBF_4_, 98%, Aldrich) were used without further purification. Ultrapure deionized water was obtained using a Sartorius Arium Comfort smart station.

All potassium salts used in this study, namely potassium trifluoro(trifluoromethyl)borate (KBF_3_CF_3_),^41^ potassium tricyanofluoroborate (KBF(CN)_3_),^42^ and potassium 2,2,2-trifluoromethylsulfonyl-*N*-cyanoamide (KTFSAM),^40^ were prepared as described previously, and their spectroscopic and elemental analysis data were in full accordance with those reported in the literature.

*N*-methylimidazole (≥99%, Aldrich), *N*-ethylimidazole (>98%, TCI Chemicals), *N*-butylimidazole (98%, Aldrich), *N*-propylimidazole (98%, Iolitec), and *N*-hexylimidazole (98%, Iolitec) were distilled over CaH_2_.

NaI (99.9%, Aldrich) was dried at 110 °C/0.1 mbar for 12 h in a B-585 oven (Buchi Glass Drying Oven, Switzerland) filled with P_2_O_5_. Afterward, it was transferred under vacuum into the argon filled glovebox (MBRAUN MB-Labstar, H_2_O and O_2_ content <0.5 ppm) and was stored there prior to further utilization.

### 2.2 Synthesis of Mono N-Substituted Imidazoles

#### 2.2.1 *N*-(2-Methyl-3-(trimethylsilyl)propyl) Imidazole

**Scheme 6:**
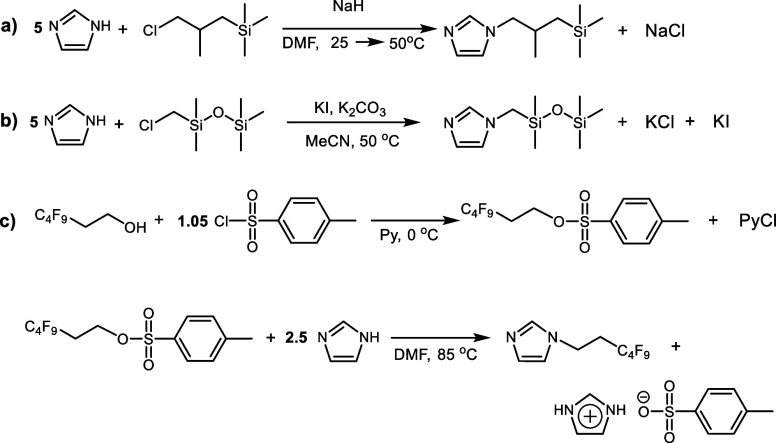
Synthetic Pathways for the Preparation of N-Substituted Imidazoles

*N*-(2-methyl-3-(trimethylsilyl)propyl) imidazole was synthesized in two steps (Scheme 6a): (1) deprotonation of imidazole with NaH in anhydrous DMF and (2) alkylation of sodium imidazole salt with (3-chloro-2-methylpropyl)-trimethylsilane, prepared separately in accordance with the previously reported procedure.^43^

Sodium hydride (8.01 g, 60 wt % dispersion in mineral oil) was loaded into the Schlenk flask equipped with a magnetic stirrer and a fritted glass filter (Schlaker reaction tube). The flask was accurately evacuated and then filled with an inert atmosphere. NaH was washed with freshly distilled *n*-hexane (3 × 50 mL) and dried at RT/0.1 mbar for 1 h to produce neat sodium hydride (4.81 g, 200.0 mmol). Further, 120 mL of anhydrous DMF were injected into the flask via a syringe under vigorous stirring. After the formation of NaH dispersion, argon flow was increased, septum was removed, and imidazole (13.63 g, 200.0 mmol) was added in several portions under inert flow (CAUTION: the addition of imidazole is accompanied by an exothermic reaction and vigorous evolution of hydrogen gas!). After stirring at RT for 30 min, the formation of a light brown solution was observed, the septum was placed back to close the Schlenk flask, and the argon flow was decreased. (3-Chloro-2-methylpropyl)-trimethylsilane (6.60 g, 40.0 mol) was added to the reaction solution dropwise via a syringe. The reaction was continued for 10 h at RT and then for 30 h at 50 °C. The resultant inorganic precipitate was filtered off, and the filtrate was diluted with 450 mL of benzene. The solution was slowly poured into ultrapure water (1250 mL) under stirring, and the organic layer was separated. The aqueous layer was washed with benzene (5 × 50 mL), and the organic layers were combined and dried over anhydrous MgSO_4_. Magnesium sulfate was filtered off, and benzene was evaporated under reduced pressure. The resultant yellowish liquid was dried at 50 °C/0.1 mbar for 3 h. Yield: 7.36 g (94%); ^1^H NMR (400.0 MHz, CDCl_3_): δ = 7.34 (s, 1H), 6.95 (s, 1H), 6.79 (s, 1H), 3.72–3.56 (m, 2H), 1.97–1.89 (m, 1H), 0.83 (d, *J* = 6.6 Hz, 3H), 0.52–0.28 (m, 2H), −0.05 (s, 9H); ^13^C NMR (100.6 MHz, CDCl_3_): δ = 137.2, 129.0, 118.9, 55.9, 31.6, 21.6, 19.8, −0.8; IR (KBr pellet): 3110 (m, ν_CH_), 2955 (vs, ν_CH_), 2897 (s, ν_CH_), 1507 (s), 1460 (m), 1284 (m), 1248 (vs, ν_Si–CH3_), 1110 (m), 1077 (m), 1032 (m), 910 (w), 840 (vs, ν_Si–CH_3__), 741 (m), 692 (m), 665 (w) cm^–1^ calcd for C_10_H_20_N_2_Si (196.37): C, 61.17%; H, 10.27%; Si, 14.30%; found, C, 60.52%; H, 10.50%; Si, 13.82%.

#### 2.2.2 *N*-((1,1,3,3,3-Pentamethyldisiloxaneyl)methyl) Imidazole

Imidazole (15.97 g, 235.0 mmol), 1-(chloromethyl)-1,1,3,3,3-pentamethyldisiloxane (9.35 g, 47.0 mmol), KI (0.15 g, 0.9 mmol), and K_2_CO_3_ (6.49 g, 47.0 mmol) were loaded into the high-pressure vessel. 150 mL of anhydrous acetonitrile were added into the vessel via a syringe, the vessel was flushed with argon, closed, and the stirring was continued at RT until the formation of fine dispersion. Then, the temperature was increased to 80 °C, and the reaction was carried out additionally for 2 days. Afterward, the reaction mass was cooled down to RT, and the inorganic precipitate was filtered off. Acetonitrile was evaporated under reduced pressure to give a yellowish solid residue, which was further dissolved in 100 mL of H_2_O. The aqueous solution was extracted with *n*-hexane (5 × 50 mL), and the organic layer was dried over anhydrous MgSO_4_. Magnesium sulfate was filtered off, and hexane was evaporated under reduced pressure. The product, representing a colorless liquid, was dried at 50 °C and 0 1 mbar for 5 h. Yield: 4.23 g (39%); ^1^H NMR (400.0 MHz, CDCl_3_): δ = 7.35 (s, 1H), 7.01 (s, 1H), 6.81 (s, 1H), 3.45 (s, 2H), 0.12 (s, 6H), 0.06 (s, 9H); ^13^C NMR (100.6 MHz, CDCl_3_): δ = 137.5, 128.9, 119.9, 39.2, 1.7, −0.9; IR (ATR-mode): 3110 (w, ν_CH_), 2955 (m, ν_CH_), 2900 (m, ν_CH_), 1507(s), 1250 (s, ν_Si–CH_3__), 1080 (s), 840 (s, ν_Si–CH_3__), 740 (m), 660 (w), 530 (w), cm^–1^ calcd for C_9_H_20_N_2_OSi_2_ (228.11): C, 47.32%; H, 8.82%; N, 12.26%; Si, 24.59%; found, C, 47.28%; H, 8.98%; N, 12.31%; Si, 24.44%.

#### 2.2.3 *N*-(3,3,4,4,5,5,6,6,6-Nonafluorohexyl) Imidazole

*N*-(3,3,4,4,5,5,6,6,6-Nonafluorohexyl) imidazole was synthesized in two steps (Scheme 6c): (1) preparation of activated 3,3,4,4,5,5,6,6,6-nonafluorohexyl-4-methylbenzenesulfonate by the reaction of 3,3,4,4,5,5,6,6,6-nonafluorohexan-1-ol with 4-toluenesulfonyl chloride and (2) subsequent imidazole alkylation by 3,3,4,4,5,5,6,6,6-nonafluorohexyl-4-methylbenzenesulfonate.

The solution of 3,3,4,4,5,5,6,6,6-nonafluorohexan-1-ol (10.00 g, 37.9 mmol) in 15 mL of pyridine was added dropwise to the solution of 4-toluenesulfonyl chloride (7.58 g, 39.7 mmol) in 30 mL of pyridine at 0 °C under inert atmosphere. The reaction mixture was stirred for 3 h at 0 °C, then gradually heated to room temperature, filtered off from the precipitated pyridine chloride, and poured into an ice-cold 2 M HCl aqueous solution. The precipitated waxy residue was washed with ultrapure water, then with saturated NaHCO_3_ aqueous solution, and finally several times with ultrapure water. Afterward, the precipitate was dissolved in 100 mL of dichloromethane and washed thoroughly with water (4 × 25 mL). The organic layer was separated, dried over anhydrous MgSO_4_, filtered, and the dichloromethane was evaporated under reduced pressure. The resultant product, representing a white waxy solid, was dried at 25 °C/0.1 mbar for 2 h. Yield: 11.36 g (72%); ^1^H NMR (400.0 MHz, DMSO-*d*_6_): δ = 7.81 (d, *J* = 8.2 Hz, 2H), 7.49 (d, *J* = 8.2 Hz, 2H), 4.29 (t, *J* = 6.7 Hz, 2H), 2.73–2.64 (m, 2H), 2.41 (s, 3H); ^13^C NMR (100.6 MHz, DMSO-*d*_6_): δ = 145.2, 131.7, 130.1, 127.6, 62.4, 29.5, 20.9; ^19^F NMR (376.5 MHz, DMSO-*d*_6_): δ = −22.6 (s), −54.8 (s), −66.0 (s), −67.6 (s).

The solution of 3,3,4,4,5,5,6,6,6-nonafluorohexyl-4-methylbenzenesulfonate (10.30 g, 24.6 mmol) in 50 mL of anhydrous DMF was added to the solution of imidazole (4.20 g, 61.7 mmol) in 10 mL of anhydrous DMF at 50 °C under an inert atmosphere. The reaction was further heated to 85 °C and carried out at this temperature for 72 h. DMF was evaporated at 80 °C/15 mbar, and the residue was extracted with 100 mL of anhydrous diethyl ether. Et_2_O solution was filtered from imidazolium tosylate, and the solvent was evaporated at 25 °C/15 mbar, providing a slightly yellow transparent oil. To remove the imidazole residue, the oil was extracted with anhydrous *n*-hexane (4 × 25 mL) and then dried at 25 °C/15 mbar. Finally, the oil was dissolved in 80 mL of dichloromethane and washed with water (3 × 10 mL), and the organic layer was dried over anhydrous MgSO_4_. Magnesium sulfate was filtered off, and dichloromethane was evaporated under reduced pressure. The product, representing colorless oil, was dried at 50 °C/0.1 mbar for 5 h. Yield: 4.21 g (54%): ^1^H NMR (400.0 MHz, CDCl_3_): δ = 7.51 (s, 1H), 7.09 (s, 1H), 6.93 (s, 1H), 4.42–4.41 (m, 2H), 2.95–2.64 (m, 2H); ^13^C NMR (100.6 MHz, CDCl_3_): δ = 137.1, 130.4, 129.7, 118.6, 69.1, 53.4, 38.8, 32.9, 31.2; ^19^F NMR (376.5 MHz, CDCl_3_): δ = −81.0 (s), −114.5 (s), −124.4 (s), −126.0 (s); IR (KBr pellet): 3112 (m, ν_CH_), 2932 (m, ν_CH_), 2859 (m, ν_CH_), 1678 (m), 1510 (s), 1439 (s), 1389 (s ν_CF_), 1354 (s, ν_CF_), 1231 (vs, ν_CF_), 1134 (s), 1100 (m, ν_CF_), 1010 (m), 878 (m), 828 (m), 750 (m), 623 (m) cm^–1^; calcd for C_9_H_7_F_9_N_2_ (314.15): C, 34.41%; H, 2.25%; F, 54.43%; found, C, 36.37%; H, 3.09%; F, 46.47%.

### 2.3 Synthesis of Poly(epiiodohydrin-*co*-ethylene Oxide)

Poly(epichlorohydrin-*co*-ethylene oxide) (3.58 g, 26.2 mmol) was dissolved in 70 mL of anhydrous acetone under an inert atmosphere at 40 °C. The freshly dried NaI (9.8 g, 65.5 mmol) was added in one portion to the flask under an inert flow. The mixture was stirred for 3 days at 50 °C; the resultant slurry was cooled down to RT and centrifuged. The yellow sticky precipitate was collected, dissolved in DMF (50 mL), and precipitated into excess H_2_O. The precipitation from DMF solution into water was repeated, and the yellowish rubber-like polymer was collected, washed with water, and dried at 60 °C/0.1 mbar for 1 day. Yield: 5.3 g (90%); ^1^H NMR (400.0 MHz, DMSO-*d*_6_): δ = 3.77–3.58 (m, 3H), 3.53 (br. s., 4H), 3.46–3.26 (m, 2H); ^13^C NMR (100.6 MHz, DMSO-*d*_6_): δ = 77.7, 76.7, 70.0, 68.5, 44.3.

### 2.4 Synthesis of Halide PILs

#### 2.4.1 Quaternization of Mono N-Substituted Imidazoles with Poly(epichlorohydrin-*co*-ethylene Oxide)

**Scheme 7:**
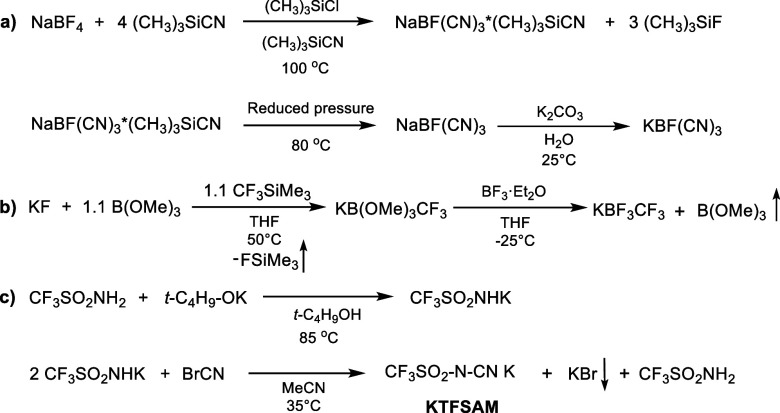
Synthetic Pathways for the Preparation of Potassium Salts with Asymmetric Anions

The majority of chloride PILs were synthesized via the quaternization reaction of respective mono N-substituted imidazoles by poly(epichlorohydrin-*co*-ethylene oxide) following the general procedure (Scheme 7) provided for the preparation of poly(1-methyl-3-[oxiran-2-ylmethyl]-1-imidazole-3-ium-*co*-ethylene oxide) chloride below.

Poly(epichlorohydrin-*co*-ethylene oxide) (3.58 g, 26.2 mmol) was dissolved in 40 mL of anhydrous DMF under an inert atmosphere on stirring at 70 °C in a high-pressure vessel (CAUTION: the dissolution can take up to 12 h). *N*-methylimidazole (21.48 g, 262.0 mmol) was added in one portion to the polymer solution under an inert flow. The temperature was then raised to 90 °C, and stirring was continued for 3 days. The viscous solution was cooled, and ∼1/2 of the solvent was removed under reduced pressure at 65 °C. Afterward, the polymer was precipitated into excess acetone. The resultant poly(1-methyl-3-[oxiran-2-ylmethyl]-1-imidazole-3-ium-*co*-ethylene oxide) chloride was obtained as a sticky light brown solid. It was thoroughly washed with acetone and dried at 70 °C and 0 1 mbar for 1 day. Yield: 5.33 g (93%); ^1^H NMR (400.0 MHz, D_2_O): δ = 8.77 (s, 1H), 7.53–7.51 (m, 2H), 4.48–4.33 (m, 2H), 3.92 (s, 3H), 3.70–3.61 (m, 7H); ^13^C NMR (100.6 MHz, D_2_O): δ = 136.7, 123.5, 123.2, 76.5, 70.4, 69.6, 50.0, 35.9, 30.3; IR (KBr pellet): 3151 (m), 3102 (m, ν_CH_), 2913 (w), 2877 (s, ν_CH_), 1647 (w), 1570 (m, ν_CN_), 1456 (w), 1347 (w), 1172 (s, ν_CN_), 1102 (s), 952 (w), 843 (w), 751 (w), 624 (m) cm^–1^.

#### 2.4.2 Quaternization of *N*-((1,1,3,3,3-Pentamethyldisiloxaneyl)methyl) Imidazole with Poly(epiidohydrin-*co*-ethylene Oxide)

Poly(epiiodohydrin-*co*-ethylene oxide) (2.93 g, 13.0 mmol) was dissolved in 50 mL of anhydrous DMF under an inert atmosphere at 70 °C in a high-pressure vessel. After the formation of clear solution, 1-((1,1,3,3,3-pentamethyldisiloxaneyl)methyl) imidazole (3.86 g, 16.9 mmol) was added in one portion to the reaction vessel. The temperature was raised to 80 °C (CAUTION: the increase in reaction temperature above 80 °C leads to cross-linking reactions), and the stirring was continued for 3 days. The viscous solution was cooled down to RT and precipitated into the excess of anhydrous Et_2_O. The resultant poly(1-methyl-3-[oxiran-2-ylmethyl]-1-imidazole-3-ium-*co*-ethylene oxide) iodide in a form of white sticky solid was dried at RT/0.1 mbar for 2 h. Yield: 3.97 g (67%); ^1^H NMR (400.0 MHz, DMSO_–_d_6_): δ = 8.74 (br. s., 1H), 7.73–7.79 (s, 2H), 4.54–4.12 (m, 3H), 4.12–3.47 (m, 22H), 0.16 (s, 6H), 0.05 (s, 9H).

### 2.5 Ion Metathesis

Polyelectrolytes **PIL1TFSI**-**PIL7TFSI**, **PIL4BF**_**4**_, **PIL4TFSAM**, **PIL4BF(CN)**_**3**_, and **PIL4BF**_**3**_**CF**_**3**_ were synthesized via ion metathesis between corresponding chloride PILs and metal salts following the general procedure given for poly(1-methyl-3-[oxiran-2-ylmethyl]-1-imidazole-3-ium-*co*-ethylene oxide) bis(trifluoromethylsulfonyl) imide (**PIL1TFSI**) below.

The solution of lithium bis(trifluoromethylsulfonyl)imide (13.43 g, 45.0 mmol, LiTFSI) in 30 mL of ultrapure water was added dropwise to the aqueous solution (100 mL) of poly(1-methyl-3-[oxiran-2-ylmethyl]-1-imidazole-3-ium-*co*-ethylene oxide) chloride (5.12 g, 23.4 mmol) at room temperature. The formation of a precipitate was observed immediately, and stirring was continued for 2 h at RT. The precipitated sticky product was collected, thoroughly washed with water, redissolved in acetone, and reprecipitated in H_2_O again. The polymer representing a slightly yellow sticky solid was dried at 70 °C/0.1 mbar for 2 days in a B-585 oven (Buchi Glass Drying Oven, Switzerland) filled with P_2_O_5_. Afterward, it was transferred under a vacuum into the argon filled glovebox (MBRAUN MB-Labstar, H_2_O and O_2_ content <0.5 ppm) and stored for 5 days prior to further investigation. Yield: 9.25 g (85%); *T*_g_ = −11.5 °C (DSC); *T*_onset_ = 290 °C (TGA); ^1^H NMR (400.0 MHz, acetone-*d*_6_): δ = 8.92 (s, 1H), 7.67 (s, 2H), 4.60–4.46 (m, 2H), 4.04 (s, 3H), 3.78–3.64 (m, 6H), 2.91 (s, 1H); ^13^C NMR (100.6 MHz, acetone-*d*_6_): δ = 138.0, 125.6–116.0 (q, J_CF_ = 321 Hz), 124.3, 77.3, 71.4, 70.9, 69.9, 51.1, 36.6; ^19^F NMR (376.5 MHz, acetone-*d*_6_): δ = −80.9 (s); IR (ATR-mode): 3159 (m, ν_CH_), 3121 (m, ν_CH_), 2881 (m, ν_CH_), 1572 (m), 1456 (m), 1354 (vs, ν_asSO_2__), 1195 (vs, ν_CF_), 1138 (s, ν_sSO_2__), 1058 (vs, ν_CF_), 741 (m), 618 (s), 571 (s), 512 (m) cm^–1^; calcd for C_11_H_15_F_6_N_3_O_6_S_2_ (463.03): C, 28.51%; H, 3.26%; F, 24.60%; found, C, 28.18%; H, 3.34%; F, 24.20%.

#### 2.5.1 Poly(1-ethyl-3-[oxiran-2-ylmethyl]-1-imidazole-3-ium-*co*-ethylene Oxide) Bis(trifluoromethylsulfonyl) Imide (**PIL2TFSI**)

Yield: 7.14 g (92%); *T*_g_ = −16.8 °C (DSC); *T*_onset_ = 290 °C (TGA); ^1^H NMR (400.0 MHz, DMSO-*d*_6_): δ = 9.05–9.03 (m, 1H), 7.80 (s, 1H), 7.65 (s, 1H), 4.38–4.22 (m, 4H), 3.83–3.65 (m, 2H), 3.52–3.33 (m, 5H), 1.43 (s, 3H); ^13^C NMR (100.6 MHz, DMSO-*d*_6_): δ = 136.3, 123.3, 122.7–116.3 (q, J_CF_ = 320 Hz), 121.3, 76.0, 70.0, 69.6, 59.8, 44.3, 15.1; ^19^F NMR (376.5 MHz, DMSO-*d*_6_): δ = −80.8 (s).

#### 2.5.2 Poly(1-propyl-3-[oxiran-2-ylmethyl]-1-imidazole-3-ium-*co*-ethylene Oxide) Bis(trifluoromethylsulfonyl) Imide (**PIL3TFSI**)

Yield: 5.65 g (90%); *T*_g_ = −23.9 °C (DSC); *T*_onset_ = 290 °C (TGA); ^1^H NMR (400.0 MHz, DMSO-*d*_6_): δ = 9.09 (s, 1H), 7.85 (s, 1H), 7.69 (s, 1H), 4.45–4.19 (m, 4H), 3.88–3.39 (m, 7H), 1.87 (s,2H), 1.43 (s, 3H); ^13^C NMR (100.6 MHz, DMSO-*d*_6_): δ = 137.0, 123.8–116.7 (q, J_CF_ = 320 Hz), 122.3, 76.4, 70.5, 69.7, 50.8, 50.3, 23.3, 10.7; ^19^F NMR (376.5 MHz, DMSO-*d*_6_): δ = −80.9 (s).

#### 2.5.3 Poly(1-butyl-3-[oxiran-2-ylmethyl]-1-imidazole-3-ium-*co*-ethylene Oxide) Bis(trifluoromethylsulfonyl) Imide (PIL4TFSI)

Yield: 5.65 g (80%); *T*_g_ = −27.7 °C (DSC); *T*_onset_ = 310 °C (TGA); ^1^H NMR (400.0 MHz, DMSO-*d*_6_): δ = 9.06 (s, 1H), 7.80 (s, 1H), 7.68 (s, 1H), 4.40–4.18 (m, 4H), 4.18–3.81 (m, 2H), 3.81–3.43 (m, 5H), 1.79 (s, 2H), 1.25 (s, 2H), 0.90 (s, 3H); ^13^C NMR (100.6 MHz, DMSO-*d*_6_): δ = 136.7, 123.4, 122.7–116.3 (q, J_CF_ = 320 Hz), 78.0, 70.1, 69.7, 48.8, 48.6, 44.1, 31.4, 18.7, 13.2; ^19^F NMR (376.5 MHz, DMSO-*d*_6_): δ = −80.9 (s); calcd for C_14_H_21_F_6_N_3_O_6_S_2_ (505.07): C, 33.27%; H, 4.19%; found, C, 33.06%; H, 4.11%.

#### 2.5.4 Poly(1-hexyl-3-[oxiran-2-ylmethyl]-1-imidazole-3-ium-*co*-ethylene Oxide) Bis(trifluoromethylsulfonyl) Imide (**PIL5TFSI**)

Yield: 8.01 g (85%); *T*_g_ = −23.1 °C (DSC); *T*_onset_ = 320 °C (TGA); ^1^H NMR (400.0 MHz, DMSO-*d*_6_): δ = 9.07 (s, 1H), 7.80 (s, 1H), 7.66 (s, 1H), 4.40–4.18 (m, 4H), 3.85–3.65 (m, 2H), 3.52–3.31 (m, 5H), 1.80 (s, 2H), 1.27 (s, 6H), 0.86 (s, 3H); ^13^C NMR (100.6 MHz, DMSO-*d*_6_): δ = 137.0, 123.1–116.7 (q, J_CF_ = 320 Hz), 122.6, 76.4, 70.5, 69.7, 50.3, 49.4, 30.9, 29.9, 25.6, 22.3, 14.1; ^19^F NMR (376.5 MHz, DMSO-*d*_6_): δ = −80.9 (s).

#### 2.5.5 Poly(1-(2-methyl-3-(trimethylsilyl)propyl)-3-[oxiran-2-ylmethyl]-1-imidazole-3-ium-*co*-ethylene Oxide) Bis(trifluoromethylsulfonyl) Imide (**PIL6TFSI**)

Yield: 6.79 g (89%); *T*_g_ = −9.2 °C (DSC); *T*_onset_ = 285 °C (TGA); ^1^H NMR (400.0 MHz, DMSO-*d*_6_): δ = 9.06 (br. s., 1H), 7.77 (br. s., 2H), 4.40–3.34 (m, 11H), 2.08 (s, 1H), 0.84 (s, 3H), 0.52–0.41 (m, 2H), 0.01 (s, 9H); ^13^C NMR (100.6 MHz, DMSO-*d*_6_): δ = 139.4, 126.2, 124.5–118.1 (q, J_CF_ = 320 Hz), 120.2, 70.2, 60.4, 55.6, 32.2, 32.0, 22.9, 20.4, 0.2; ^19^F NMR (376.5 MHz, DMSO-*d*_6_): δ = −80.8 (s); IR (KBr pellet): 3155 (m, ν_CH_), 3119 (m, ν_CH_), 2968 (m, ν_CH_), 2893 (m, ν_CH_), 1568 (s), 1468 (m), 1352 (vs, ν_asSO_2__), 1190 (vs, ν_CF_), 1138 (s, ν_sSO_2__), 1055 (vs, ν_CF_), 845 (m, ν_Si–CH_3__), 791 (m), 743 (w), 648 (s), 615 (m) cm^–1^.

#### 2.5.6 Poly(1-(3,3,4,4,5,5,6,6,6-nonafluorohexyl)-3-[oxiran-2-ylmethyl]-1-imidazole-3-ium-*co*-ethylene Oxide) Bis(trifluoromethylsulfonyl) Imide (PIL7TFSI)

Yield: 4.32 g (87%); *T*_g_ = 3.4 °C (DSC); *T*_onset_ = 280 °C (TGA); ^1^H NMR (400.0 MHz, DMSO-*d*_6_): δ = 9.16 (s, 1H), 7.92 (s, 1H), 7.67 (br. s., 1H), 4.60 (s, 2H), 4.41–4.21 (m, 2H), 3.84–3.48 (m, 8H), 3.00 (s, 2H); ^13^C NMR (100.6 MHz, DMSO-*d*_6_): δ = 137.3, 124.2–114.6 (q, J_CF_ = 320 Hz), 123.6, 122.3, 115.4, 76.1, 75.7, 69.6, 68.5, 49.8, 41.3, 29.9; ^19^F NMR (376.5 MHz,, DMSO-*d*_6_): δ = −81.2 (s), −83.1 (s), −116.0 (s), −126.6 (s), −128.2 (s); IR (KBr pellet): 3153 (m, ν_CH_), 3120 (m, ν_CH_), 3096 (m, ν_CH_), 2920 (m), 2882 (m, ν_CH_), 1568 (m), 1457 (w), 1352 (vs, ν_asSO_2__), 1233 (vs, ν_CF_), 1197 (vs, ν_CF_), 1134 (vs, ν_sSO_2__), 1058 (vs, ν_CF_), 881 (m), 791 (m), 740 (w), 710 (w), 617 (m), 601 (m), cm^–1^; calcd for C_16_H_16_F_15_N_3_O_6_S_2_ (695.39): C, 27.63%; H, 2.32%; S, 9.22%; found, C, 27.07%; H, 2.27%; S, 9.02%.

#### 2.5.7 Poly(1-butyl-3-[oxiran-2-ylmethyl]-1-imidazole-3-ium-*co*-ethylene Oxide) Tetrafluoroborate (**PIL4BF**_**4**_)

The same procedure was used as for **PIL1TFSI** with the exception that aqueous solution of NaBF_4_ (1.05 g, 9.6 mmol) in 10 mL was added dropwise to a solution of poly(1-butyl-3-[oxiran-2-ylmethyl]-1-imidazole-3-ium-*co*-ethylene oxide) chloride (1.25 g, 4.8 mmol) in 10 mL of ultrapure water. Yield: 0.9 g (60%); *T*_g_ = −3.6 °C (DSC); *T*_onset_ = 270 °C (TGA); ^1^H NMR (400.0 MHz, DMSO-*d*_6_): δ = 9.03 (br. s., 1H), 7.80 (s, 1H), 7.65 (br. s., 1H), 4.37–4.19 (m, 4H), 4.00–3.37 (m, 7H), 1.77 (s, 2H), 1.24 (s, 2H), 0.89 (s, 3H); ^13^C NMR (100.6 MHz, DMSO-*d*_6_): δ = 136.6, 123.4, 122.2, 75.9, 70.1, 69.7, 49.6, 48.7, 44.1, 31.4, 18.7, 13.2; ^19^F NMR (376.5 MHz, DMSO-*d*_6_): δ = −150.5 (s); ^11^B NMR (192.5 MHz, DMSO-*d*_6_): δ = −1.3 (s); IR (ATR-mode): 3153 (w, ν_CH_), 3116 (w, ν_CH_), 2963 (w, ν_CH_), 2934 (w, ν_CH_), 2875 (w, ν_CH_), 1564 (m), 1465 (m), 1344 (w), 1167 (m, ν_CO_), 1046 (vs), 1033 (vs, ν_BF_), 848 (m), 749 (w), 642 (m) cm^–1^; calcd for C_14_H_21_F_3_N_4_O_4_S (312.12): C, 46.18%; H, 6.78%; found, C, 45.69%; H, 6.93%.

#### 2.5.8 Poly(1-butyl-3-[oxiran-2-ylmethyl]-1-imidazole-3-ium-*co*-ethylene Oxide) 2,2,2-Trifluoromethylsulfonyl-*N*-cyanoamide (**PIL4TFSAM**)

The same procedure was used as for **PIL1TFSI** with the exception that the aqueous solution of KTFSAM (1.1 g, 5.2 mmol) in 10 mL was added dropwise to a solution of poly(1-butyl-3-[oxiran-2-ylmethyl]-1-imidazole-3-ium-*co*-ethylene oxide) chloride (0.68 g, 2.6 mmol) in 10 mL of water. Yield: 0.9 g (86%); *T*_g_ = −30.1 °C (DSC); *T*_onset_ = 260 °C (TGA); ^1^H NMR (400.0 MHz, DMSO-*d*_6_): δ = 9.06 (br. s., 1H), 7.80 (s, 1H), 7.65 (br. s., 1H), 4.38–4.19 (m, 4H), 4.00–3.37 (m, 7H), 1.78 (s, 2H), 1.26 (s, 2H), 0.90 (s, 3H); ^13^C NMR (100.6 MHz, DMSO-*d*_6_): δ = 136.6, 123.4, 122.2, 123.5–117.1 (q, *J*_CF_ = 325 Hz), 114.3, 75.8, 70.1, 69.7, 68.5, 49.6, 48.7, 44.1, 31.4, 18.7, 13.2; ^19^F NMR (376.5 MHz, DMSO-*d*_6_): δ = −79.9 (s); IR (ATR-mode): 3147 (w, ν_CH_), 3110 (w, ν_CH_), 2963 (w, ν_CH_), 2935 (w, ν_CH_), 2876 (w, ν_CH_), 2359 (w), 2342 (w), 2187 (s, ν_CN_), 1563 (m), 1464 (m), 1328 (s, ν_asSO_2__), 1212 (vs, ν_CF_), 1165 (s, ν_sSO_2__), 1114 (vs, ν_CF_), 829 (s), 751 (w), 635 (s) cm^–1^; calcd for C_14_H_21_F_3_N_4_O_4_S (398.40): C, 42.21%; H, 5.31%; F, 14.31%; found, C, 41.47%; H, 5.33%; F, 13.9%.

#### 2.5.9 Poly(1-butyl-3-[oxiran-2-ylmethyl]-1-imidazole-3-ium-*co*-ethylene Oxide) Tricyanofluoroborate (**PIL4BF(CN)**_**3**_)

The same procedure was used as for **PIL1TFSI** with the exception that an aqueous solution of KBF(CN)_3_ (1.30 g, 8.8 mmol) in 10 mL was added dropwise to a solution of poly(1-butyl-3-[oxiran-2-ylmethyl]-1-imidazole-3-ium-*co*-ethylene oxide) chloride (1.15 g, 4.4 mmol) in 10 mL of water. Yield: 1.3 g (92%); *T*_g_ = −26.4 °C (DSC); *T*_onset_ = 250 °C (TGA); ^1^H NMR (400.0 MHz, DMSO-*d*_6_): δ = 9.06 (br. s., 1H), 7.80 (s, 1H), 7.65 (br. s., 1H), 4.38–4.19 (m, 4H), 4.00–3.37 (m, 7H), 1.78 (s, 2H), 1.26 (s, 2H), 0.90 (s, 3H); ^13^C NMR (100.6 MHz, DMSO-*d*_6_): δ = 136.7, 126.5 (m), 123.4, 122.2, 75.9, 70.1, 69.7, 49.6, 48.7, 44.1, 31.4, 18.7, 13.2; ^19^F NMR (376.5 MHz, DMSO-*d*_6_): δ = −213.3- −213.0 (m); ^11^B NMR (192.5 MHz, DMSO-*d*_6_): δ = −17.9 (d, *J* = 44.7 Hz); IR (ATR-mode): 3149 (m, ν_CH_), 3113 (w, ν_CH_), 2962 (w, ν_CH_), 2934 (w, ν_CH_), 2877 (w, ν_CH_), 2214 (w, ν_CN_), 1562 (m), 1463 (m), 1344 (w), 1164 (m, ν_CO_), 1097 (vs), 1051 (vs, ν_F_), 939 (m) 902 (vs), 748 (w), 630 (s) cm^–1^; calcd for C_15_H_21_BFN_5_O_2_ (333.17): C, 54.08%; H, 6.35%; B, 3.24%; found, C, 52.30%; H, 6.19%; B, 3.03%.

#### 2.5.10 Poly(1-butyl-3-[oxiran-2-ylmethyl]-1-imidazole-3-ium-*co*-ethylene Oxide) Trifluoro(trifluoromethyl)borate (**PIL4BF**_**3**_**CF**_**3**_)

The same procedure was used as for **PIL1TFSI** with the exception that an aqueous solution of KBF_3_CF_3_ (1.56 g, 8.8 mmol) in 10 mL was added dropwise to a solution of poly(1-butyl-3-[oxiran-2-ylmethyl]-1-imidazole-3-ium-*co*-ethylene oxide) chloride (1.15 g, 4.4 mmol) in 10 mL of water. Yield: 1.4 g (89%); *T*_g_ = −15.5 °C (DSC); *T*_onset_ = 185 °C (TGA); ^1^H NMR (400.0 MHz, DMSO-*d*_6_): δ = 9.05 (br. s., 1H), 7.80 (s, 1H), 7.65 (br. s., 1H), 4.38–4.19 (m, 4H), 4.00–3.37 (m, 7H), 1.77 (s, 2H), 1.25 (s, 2H), 0.89 (s, 3H); ^13^C NMR (100.6 MHz, DMSO-*d*_6_): δ = 136.5, 123.3, 122.1, 76.1, 70.1, 68.5, 49.8, 48.6, 44.1, 31.4, 18.7, 13.2; ^19^F NMR (376.5 MHz, DMSO-*d*_6_): δ = −76.5 (dd, *J* = 64.5, 31.7 Hz, CF_3_), −156.4 (dd, *J* = 79.3, 39.2 Hz, F); ^11^B NMR (192.5 MHz, DMSO-*d*_6_): δ = - 1.5 (m); IR (ATR-mode): 3156 (m, ν_CH_), 3116 (w, ν_CH_), 2963 (w, ν_CH_), 2936 (w, ν_CH_), 2877 (w, ν_CH_), 1564 (m), 1465 (m), 1344 (w), 1252 (w), 1166 (m, ν_CO_), 1045 (vs, ν_BF_), 975 (s), 949 (vs, νc_F_) 843 (m), 747 (w), 633 (s) cm^–1;^ calcd for C_13_H_21_BF_6_N_2_O_2_ (362.12): C, 43.12%; H, 5.85%; B, 2.99%; found, C, 42.65%; H, 5.90%; B, 2.73%.

#### 2.5.11 Poly(1-((1,1,3,3,3-pentamethyldisiloxaneyl)methyl)-3-[oxiran-2-ylmethyl]-1-imidazole-3-ium-*co*-ethylene Oxide) Bis(trifluoromethylsulfonyl) Imide (**PIL8TFSI**)

Due to the high tendency to hydrolysis, ion metathesis of the respective iodide polyelectrolyte with LiTFSI was performed in anhydrous acetone.

The solution of LiTFSI (5.01 g, 17.4 mmol) in 10 mL of anhydrous acetone was added dropwise to the solution of poly(1-methyl-3-[oxiran-2-ylmethyl]-1-imidazole-3-ium-*co*-ethylene oxide) iodide (3.97 g, 8.7 mmol) in 20 mL of anhydrous acetone. The solution was further stirred for 1 h at RT, whereupon the product was precipitated into the excess of anhydrous Et_2_O. The yellowish sticky polymer was collected, washed with Et_2_O, and dried at 60 °C/0.1 mbar for 1 d. Yield: 3.95 g (50%); *T*_g_ = −0.6 °C (DSC); *T*_onset_ = 185 °C (TGA); ^1^H NMR (400.0 MHz, DMSO-*d*_6_): δ = 8.95 (br. s., 1H), 7.72 (s, 1H), 7.58 (br. s., 1H), 4.61–4.01 (m, 2H), 4.00–3.40 (m, 22H), 0.16 (s, 6H), 0.05 (s, 9H); ^13^C NMR (100.6 MHz, DMSO-*d*_6_): δ = 135.8, 123.3, 122.9, 122.6–116.2 (q, J_CF_ = 320 Hz), 77.6, 75.9, 69.7, 68.7, 41.2, 29.6, 1.7,-1.1; ^19^F NMR (376.5 MHz,, DMSO-*d*_6_): δ = −79.4 (s); IR (KBr pellet): 3153 (m, ν_CH_), 3120 (m, ν_CH_), 3096 (m, ν_CH_), 2920 (m), 2882 (m, ν_CH_), 1568, 1457, 1352 (vs, ν_asSO2_), 1197 (vs, ν_CF_), 1134 (vs, ν_sSO_2__), 1058 (vs, ν_CF_), 861 (m, ν_Si–CH_3__), 791 (m), 740 (w), 710 (w), 617 (m), 601 (m), cm^–1;^ calcd for C_16_H_29_F_6_N_3_O_7_S_2_Si_2_ (609.66): C, 31.52%; H, 4.79%; found, C, 31.43%; H, 5.12%.

## 3 Results and Discussion

### 3.1 Synthesis of Mono N-Substituted Imidazoles

All novel N-substituted imidazoles were synthesized via a *N*-alkylation reaction in the presence of an excess of imidazole to prevent unwanted quaternization (Scheme 6).

Synthesis of *N*-(2-methyl-3-(trimethylsilyl)propyl) imidazole consisted of two reaction steps: deprotonation of imidazole with NaH in DMF and subsequent alkylation of sodium imidazole-1-ide with (3-chloro-2-methylpropyl)-trimethylsilane (Scheme 6a). The isolation of the product and its purification via the extraction method provided a yellowish liquid in nearly quantitative yield (94%).

The same procedure was not applicable for the synthesis of *N*-((1,1,3,3,3-pentamethyldisiloxaneyl)methyl) imidazole (Scheme 6b), as the siloxane fragment in the alkylating agent easily underwent alcoholysis in the presence of a strong base, such as sodium imidazole-1-ide. Switching to a less basic K_2_CO_3_ was not efficient as the nascent intermediate, namely, potassium imidazole-1-ide, still partially attacked both the alkylating agent and the final product. Thus, it was necessary to increase the rate of the main reaction and to suppress the side alcoholysis reaction. First, a small amount of KI was added to perform in situ the exchange of the chloride atom with the iodide one in the alkylating agent and to enhance its reactivity. Second, the amount of K_2_CO_3_ was decreased to reduce the formation of potassium imidazole-1-ide. Both the transition to 1-(iodomethyl)-1,1,3,3,3-pentamethyldisiloxane with higher reactivity and the decrease in the excess of the potassium imidazole-1-ide allowed for the preparation of targeted *N*-((1,1,3,3,3-pentamethyldisiloxaneyl)methyl) imidazole as a colorless liquid in 40% yield.

Due to the commercial availability of 3,3,4,4,5,5,6,6,6-nonafluorohexan-1-ol, the *N*-(3,3,4,4,5,5,6,6,6-nonafluorohexyl) imidazole was synthesized via another route (Scheme 6c). On the first step, activated 3,3,4,4,5,5,6,6,6-nonafluorohexyl-4-methylbenzenesulfo-nate was obtained by the reaction of 4-toluenesulfonyl chloride with 3,3,4,4,5,5,6,6,6-nonafluorohexan-1-ol. Pyridine was used in this reaction simultaneously as the solvent, catalyst, and HCl acceptor. The second step involved the alkylation of imidazole with 3,3,4,4,5,5,6,6,6-nonafluorohexyl-4-methylbenzenesulfonate in anhydrous DMF. Imidazole was taken in excess to form the desired *N*-(3,3,4,4,5,5,6,6,6-nonafluorohexyl) imidazole and to act as a scavenger of 4-methylbenzenesulfonic acid. The product was obtained in 70% yield as a white, waxy solid.

The structure and purity of synthesized mono N-substituted imidazoles were confirmed by ^1^H, ^13^C, and ^19^FNMR and IR spectroscopy as well as by elemental analysis (see section II in the Supporting Information).

### 3.2 Synthesis of Potassium Salts with Asymmetric Anions

Three potassium salts with various asymmetric anions were prepared using synthetic routes depicted in Scheme 7. KBF(CN)_3_ was synthesized using the method reported by Ignat’ev et al.^42^ The first step involved the substitution reaction between sodium tetrafluoroborate and trimethylsilyl cyanide in the presence of a trimethylsilyl chloride as a catalyst (Scheme 7, a). Solvate-free high purity potassium salt was obtained by heating to 80 °C under reduced pressure and subsequent treatment with aqueous hydrogen peroxide and K_2_CO_3_ solutions.

KBF_3_CF_3_ was obtained using the improved procedure proposed by our group recently (Scheme 7b).^41^ The main advantage of the suggested method is the substitution of corrosive HF with the more benign BF_3_*Et_2_O complex as a source of fluorine atoms.

Finally, the so-called KTFSAM salt was synthesized in accordance with the procedure developed by our group previously (Scheme 7c).^40^ It consists of the two reaction steps, namely, the reaction of the trifluorosulfoneamine with potassium *tert*-butoxide and further interaction of the as prepared CF_3_SO_2_NHK salt with BrCN in acetonitrile solution.

All potassium salts used in this study were characterized by elemental analysis and NMR and IR spectroscopy. Their spectroscopic data were in a full accordance with the data reported in the literature.^40−42^

### 3.3 Synthesis of Halide PILs

**Scheme 8:**
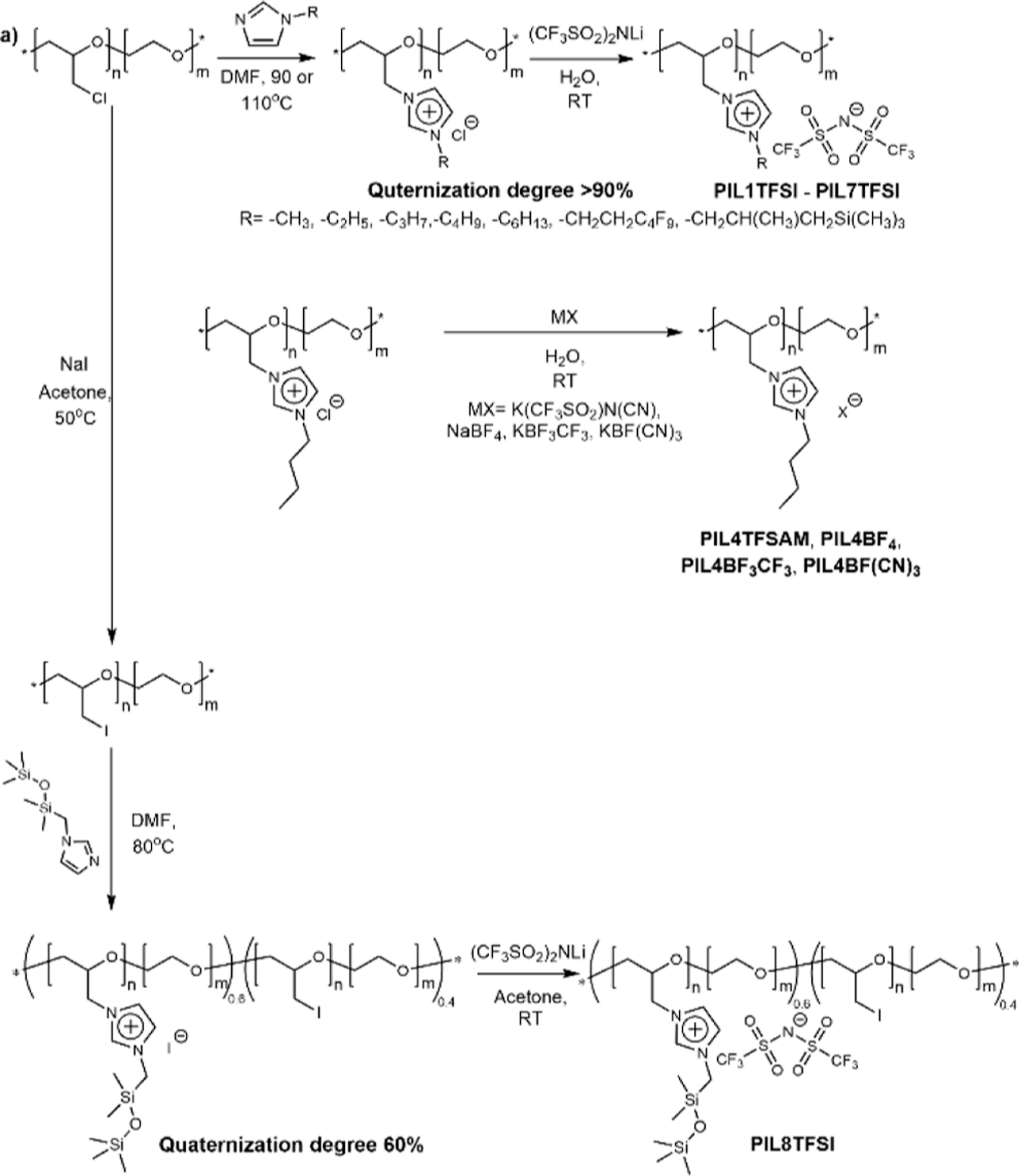
Synthesis of Cationic PILs via the Modification Idealized Structures Shown for All Polymers where Quaternization Was Effectively Complete (Table S1)

Commercially available Hydrin poly(epichlorohydrin-*co*-ethylene oxide) copolymer with high molecular weight (see Figures S10–S11) was selected as a starting material for the synthesis of PILs as the presence of additional ethylene oxide fragments in the backbone in comparison with the epichlorohydrin homopolymer allowed to reduce charge density, increase backbone flexibility, promote ion solvation, and increase their mobility. The general reaction pathway for the synthesis of novel cationic PILs consisted of two steps (Scheme 8): (1) quaternization reaction of poly(epichlorohydrin-*co*-ethylene oxide) with an excess of respective mono N-substituted imidazole in DMF and (2) ion metathesis between chloride PIL and selected metal salts in aqueous medium. The suggested approach differs by its simplicity and allows avoiding the complicated ring opening polymerization used by Baker et al.^28^

**Figure 1:**
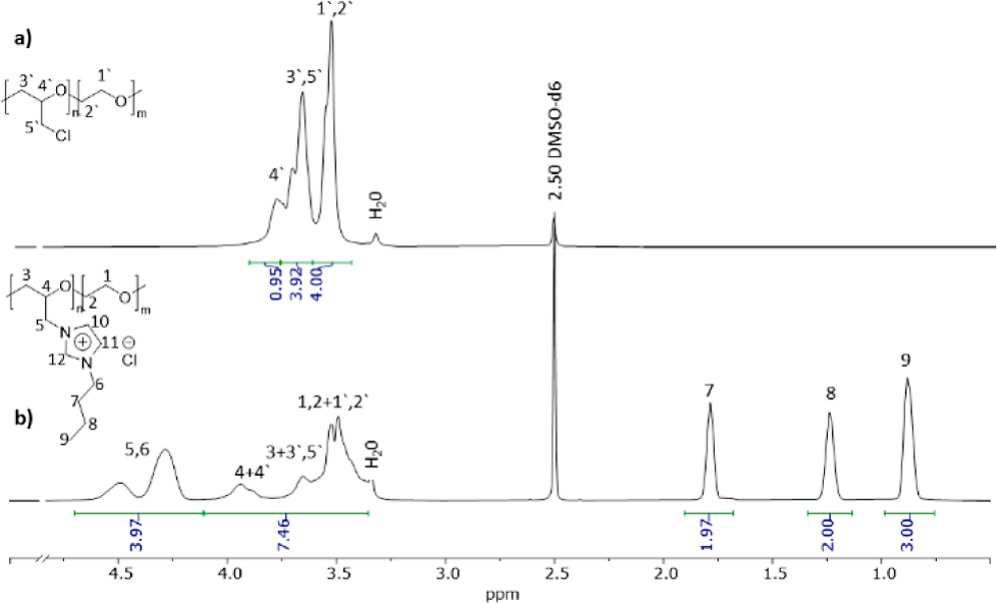
Comparison of ^1^H NMR of poly(epichlorohydrin-*co*-ethylene oxide) (a) and poly(1-butyl-3-[oxiran-2-ylmethyl]-1-imidazole-3-ium-*co*-ethylene oxide) chloride (**PIL4Cl**) (b). (For full and detailed ^1^H NMR spectra, please see the Supporting Information file).

The quaternization degree was determined using NMR spectroscopy (Figure 1, Section VII in the Supporting Information file and Table S1). Comparing ^1^H NMR spectra of neat poly(epichlorohydrin-*co*-ethylene oxide) and quaternized polymer (poly(1-butyl-3-[oxiran-2-ylmethyl]-1-imidazole-3-ium-*co*-ethylene oxide) chloride in the given example), it can be seen that signals 4 and 5, assigned to CH and CH_2_ groups, respectively, after modification moved to downfield while the other signals stayed almost in the same position (Figure 1). The calculation of quaternization degree (see Table S1) was performed by comparison of real and theoretical integral values for signals 1–4 in the 3.34–3.82 ppm region.

The study of the temperature effect on the completeness of the quaternization step revealed that 80 °C is the minimum required temperature to achieve a modification degree above 85%. For short alkyl chains (from *N*-methyl to *N*-propyl imidazole), the performance of the reaction at 90 °C was sufficient to reach 93–95% degree of quaternization, while for long substitutes, the temperature of 110 °C was necessary to gain comparable modification. Next, the influence of N-substituted imidazole excess on the quaternization degree was investigated. It was found that for the achievement of high reaction conversions, the 10-fold excess of the respective N-substituted imidazole is required. Finally, the application of the determined optimized reaction conditions (10-fold excess of N-substituted imidazole, 80 °C for *N*-methyl-, *N*-ethyl-, and *N*-propyl imidazoles, 110 °C for other N-substituted imidazoles) allowed to reach 90–97% quaternization degree for all synthesized chloride PILs (Table S1).

The same conditions were not applicable for the synthesis of the halide precursor of **PIL8TFSI** (Scheme 8) with siloxane side chains, as the excess of monosubstituted imidazole even in the presence of traces of water at high temperatures was leading to the hydrolysis of siloxane bonds and further polymer degradation and cross-linking. For that reason, the excess of *N*-((1,1,3,3,3-pentamethyldisiloxaneyl)methyl) imidazole was reduced from 10 to 1.3 equivalents, the temperature was decreased to 80 °C, and more reactive poly(epiiodohydrin-*co*-ethylene oxide) was used. These precautions provided fully soluble iodide PIL with a degree of quaternization equal to 60%.

### 3.4 Ion Metathesis

To obtain PILs with TFSI anions (Schemes 5 and 7, **PIL1TFSI**–**PIL7TFSI**), ion metathesis with LiTFSI salt was conducted in an aqueous medium. The application of a small excess of LiTFSI and the hydrophobic nature of the formed polyelectrolytes resulted in the precipitation of PILs during the ion exchange process. The subsequent additional precipitation from acetone solution into an excess of water provided the desired **PIL1TFSI–PIL7TFSI** in 80–90% yield with high purity. Depending on the substituents at the imidazolium cation, **PIL1TFSI–PIL7TFSI** represented yellowish cold-flowing transparent rubbers with various viscosities.

However, the ion metathesis in an aqueous medium was not applicable for the synthesis of **PIL8TFSI** with a siloxane side chain (Scheme 7). To avoid the degradational impact of water, the ion exchange with LiTFSI was conducted in anhydrous acetone, while the final product was precipitated into an excess of anhydrous Et_2_O.

Finally, the chloride precursor of PIL4 with an *n*-butyl substituted imidazolium cation has been selected for the ion exchange with potassium salts bearing asymmetric anions (Scheme 8). Such a choice was driven by the fact that PIL4TFSI was demonstrating the highest ionic conductivity among synthesized TFSI-based PILs (see Section 3.6). The ion metathesis with potassium salts in aqueous medium resulted in high-purity PILs obtained with sufficient yields of 86–92%. Both **PIL4TFSAM**, **PIL4BF**_**3**_**CF**_**3**_, and **PIL4BF(CN)**_**3**_ were precipitated in the course of the ion exchange reaction. It is necessary to mention that neither BF_3_CF_3_ nor BF(CN)_3_ anions were previously used for the preparation of PILs, thus making **PIL4BF**_**3**_**CF**_**3**_ and **PIL4BF(CN)**_**3**_ the first examples in the field.

**Figure 2:**
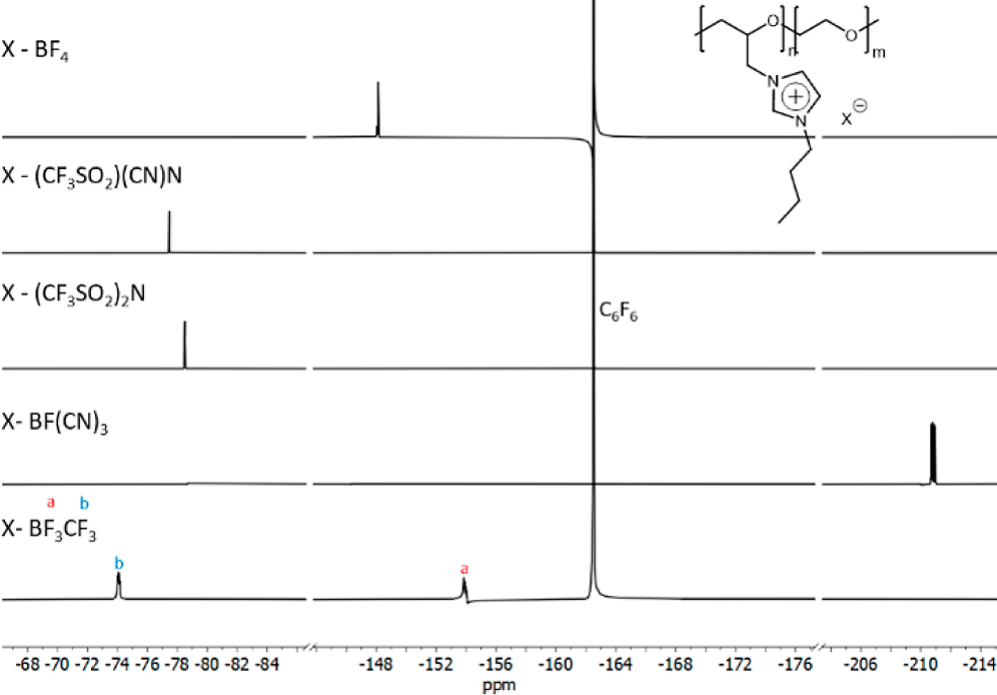
^19^F NMR spectra of *n*-butyl-substituted PILs with different anions.

The absence of the chloride anions after ion metathesis was confirmed by a simple test with silver nitrate. The structure and purity of PILs were further proved by elemental analysis and NMR (Figures S2–S7) and IR (Figure S9) spectroscopy. The detailed assignment of ^1^H NMR spectra of PILs is represented in the experimental part (seeSection V and Figures S2–S7, S9 in the Supporting Information). Similar to ILs,^12^ the significant change in chemical shift for protons of the imidazolium cation was observed after the ion exchange from the chloride anion. All PILs with delocalized anions (TFSI, TFSAM, BF_4_, BF_3_CF_3_, and BF(CN)_3_) showed the chemical shift of the imidazolium ring protons compared to the chloride precursor, although this shift was nearly identical. The comparison of the fluorine chemical shifts in ^19^F NMR spectra of synthesized PILs is shown in Figure 2. As the negative inductive effect of the substituent on the boron atom increases, the chemical shift of the fluorine moves to the higher field, reaching approximately −211 ppm for **PIL4** with the BF(CN)_3_ anion. A similar dependence can be seen for **PIL4TFSI** and **PIL4TFSAM**, showing the transition from −80.9 to −79.9 ppm for TFSI and TFSAM anions, respectively.

Previously, it was common to associate the level of conductivity in PILs with the delocalization of charge on the anion and cation.^1^ However, in the case of the three boron anions used in the current work, it is more appropriate to discuss the electron density on the boron atom rather than the charge delocalization because, in all instances, the boron atom is in an sp^3^ hybridization state and forms only sigma bonds with its substituents (no π-bonds). To compare the electron densities on the boron atoms within BF_4_, BF_3_CF_3_, and BF(CN)_3_ anions, ^11^B NMR spectroscopy was additionally employed (Figure S7). The resulting chemical shifts for **PIL4BF**_**4**_, **PIL4CF**_**3**_**BF**_**3**_, and **PIL4BF(CN)**_**3**_ were found to be 1.26, −1.43, and −17.94 ppm, respectively (Figure S7). Based on these results, it can be concluded that the boron atom in the BF_4_ anion has a similar level of electron density as the boron atom in BF_3_CF_3_.^44^ In its turn, the boron atom in BF_3_CF_3_ is less electron-rich than the boron atom in the BF(CN)_3_ anion.^45,46^ This trend aligns with the strength of the negative inductive effect of the substituents attached to the boron atom (F ≈ CF_3_ > CN).^47,49^ Based on these discussions, the lower electron density on the boron atom in the BF_4_ anion suggests that the interaction energy between the chemically bonded cation and counteranion in **PIL4BF**_**4**_ should be lower than that in **PIL4CF**_**3**_**BF**_**3**_ and **PIL4BF(CN)**_**3**_. Consequently, this would imply better ion-conducting properties for **PILBF**_**4**_ (see Section 3.6.). However, the electron density distribution in the anion is not the sole factor influencing the conductivity of the PILs and ILs. Recently, the structure-dependent physical properties of the ionic liquid electrolytes were discussed in terms of the van der Waals radius, the atomic charge distribution over the anion backbones, the interaction energy of the anion and cation coupled with the presence of ion pairs, and the size and asymmetry of the anion.^45^ Thus, the complex effect of anion structure on ion pairing can be further understood by analyzing the ^1^H NMR spectra of a series of imidazolium ILs with the same anions (Figure S8). The transition from BF_4_ to CF_3_BF_3_ and then to B(CN)_4_ results in a deshielding of the acidic proton C2 on the imidazolium cation, with a corresponding change in its chemical shift from 8.94 to 9.08 and then to 9.10 ppm (Figure S8). This observation in accordance with the previously published reports^48^ highlights the fact that the interaction between the B(CN)_4_ anion and imidazolium cation is lower than in the case of the BF_4_ anion, thus further explaining the difference in conductivity (see Section 3.6.) and *T*_g_ (see Section 3.5.) of the respective PILs.

**Figure 3:**
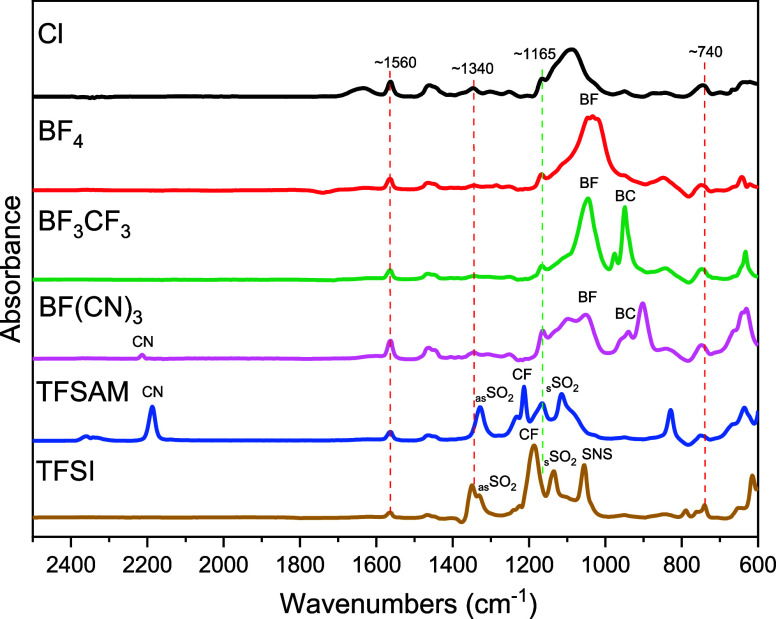
IR spectra of *n*-butyl substituted PILs with different anions (simplified spectra; for full spectra, see Figure S9).

PILs were further characterized by IR spectroscopy (Figures 3 and S9). Characteristic bands for imidazolium ring (red dashed lines) at ∼740 (ν(CH_2_(N))), ∼1337 (ν(CN_ring_)), and ∼ 1568 (ν(CC_ring_)) cm^–1^ as well as for the ether groups (green dashed lines) at ∼ 1165 (ν(COC)) cm^–1^ were found in all analyzed samples.^49^ Polymers **PILBF**_**4**_, **PIL4BF**_**3**_**CF**_**3**_, and **PIL4BF(CN)**_**3**_ with boron-based anions showed signals at ∼ 1062 (ν(BF)) cm^–1^. In addition, **PIL4BF**_**3**_**CF**_**3**_ and **PIL4BF(CN)**_**3**_ demonstrated a signal at ∼940 cm^–1^, attributed to the B–C bond vibration. The signal at ∼2200 cm^–1^ assigned to the vibration of the C–N group was found for **PIL4TFSAM** and **PIL4BF(CN)**_**3**_.^49,50^ Commonly, the intensity of the band characteristic for the inactivated CN group is low.^51^ In the case of the TFSAM anion, the CF_3_SO_2_–*N*-fragment plays the role of a strong electron acceptor group that results in high polarization of the CN group and causes a high intensity of the signal at 2187 cm^–1^. Considering PIL with BF(CN)_3_ anion, the electron acceptor effect of only one fluorine atom is distributed/divided to the three CN groups that results in their insufficient polarization and subsequently in weak intensity of the band at 2214 cm^–1^ (Figures 3 and S9). The characteristic bands of sulfonylimide anions were observed in **PIL4TFSI** at ∼1332 (asymmetric SO_2_), ∼1187 (CF), ∼1136 (symmetric SO_2_), and ∼1056 (SNS) cm^–1^, correspondingly. At the same time, for **PIL4TFSAM**, the signal at 1060 cm^–1^ was absent, and the CF stretching signal was shifted to 1218 cm^–1^, in full agreement with published reports.^40,49^

### 3.5 PILs Thermal Properties

**Figure 4:**
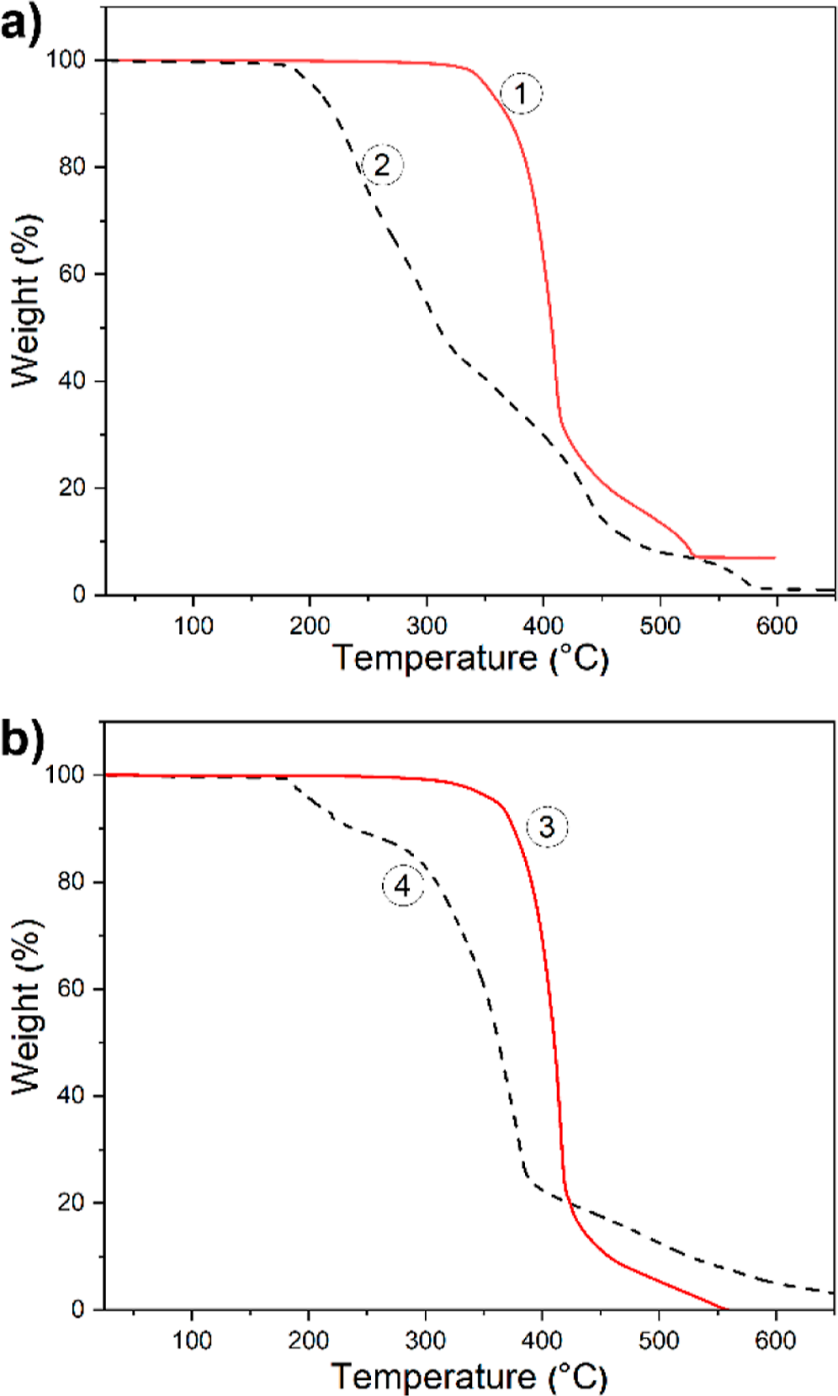
Comparison of TGA traces for **PIL5TFSI** (1), **PIL8(SiOSi)TFSI** (2), **PIL4TFSI** (3), and **PIL4BF**_**3**_**CF**_**3**_ (4).

The thermal degradation behavior of PILs was investigated by thermogravimetric analysis in air (Figure 4 and Table 1). For TFSI-based PILs, the decomposition temperatures with respect to the chemical structure are represented below by the following order:

**Table 1:**
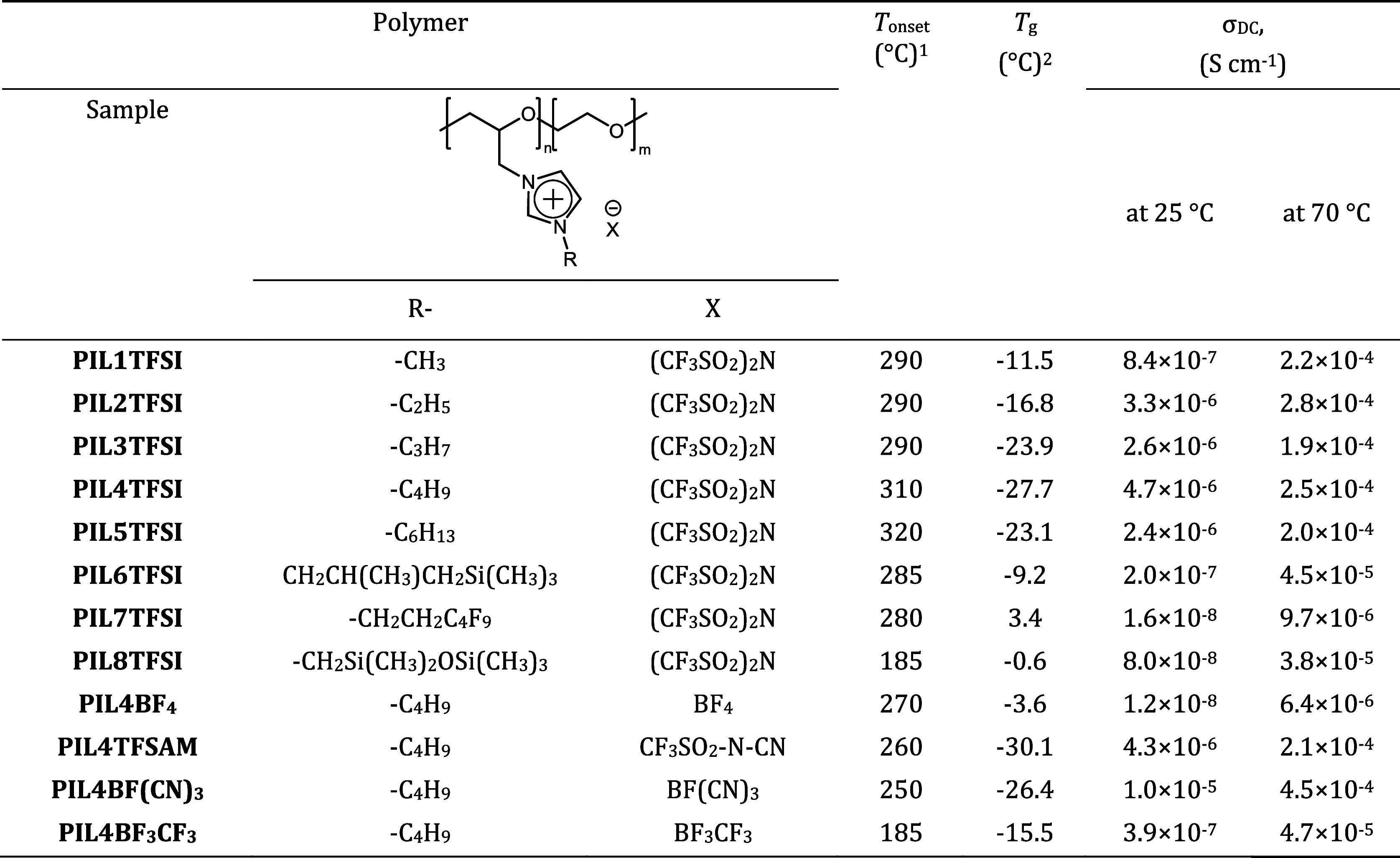
Selected Properties of PILs

1 Onset mass loss temperature by TGA in air at a heating rate of 5 °C min^–1^.

2 By DSC in N_2_ at a heating rate of 5 °C min^–1^.

*T*_onset_ (°C): **PIL5(C_6_)TFSI** (320) > **PIL4(C_4_)TFSI** (310) > **PIL3(C_3_)TFSI** (290) ≈ **PIL2(C_2_)TFSI** (290) ≈ **PIL1(C_1_)TFSI** (290) > **PIL6(Si(CH_3_)_3_)TFSI** (285) > **PIL7(CF_3_)TFSI** (280) > **PIL8(SiOSi)TFSI** (185 °C).

Among TFSI PILs, all samples with alkyl side chains showed satisfactory thermal stability, with onset thermal degradation ranging from 290 to 320 °C. In contrast, polymers with heteroatoms in the imidazole substituent demonstrated slightly decreased thermal stability, with the lowest one *T*_onset_ = 185 °C for **PIL8TFSI** having a siloxane side chain. The later is in full agreement with the thermal behavior of liner or branched polydimethylsiloxanes that commonly possess a *T*_onset_ of around 150 °C.^52^ The transfer from TFSI to BF4 anion in PIL4 was accompanied by a decrease in the onset weight loss temperature of 310 to 270 °C, which was in agreement with the data published for PILs previously.^53^ The introduction of the CN group into the anion structure resulted in a further decrease of PIL thermal stability from 310 to 260 °C for TFSI and TFSAM representatives and from 270 to 250 °C for BF_4_ and BF(CN)_3_-based PILs (Table 1). Finally, **PIL4BF**_**3**_**CF**_**3**_ showed the lowest thermal stability, possibly due to a high tendency of the BF_3_CF_3_ anion toward elimination of the CF_2_ moiety and formation of BF_4_ anion along with other byproducts.^53^ The decomposition temperatures of PILs with respect to the chemical structure of their anions can be represented in the following order:

*T*_onset_ (°C) **PIL4TFSI** (310) > **PIL4BF**_**4**_ (270) > **PIL4TFSAM** (260) > **PIL4BF(CN)**_**3**_ (250) > **PIL4BF**_**3**_**CF**_**3**_ (185 °C)

**Figure 5:**
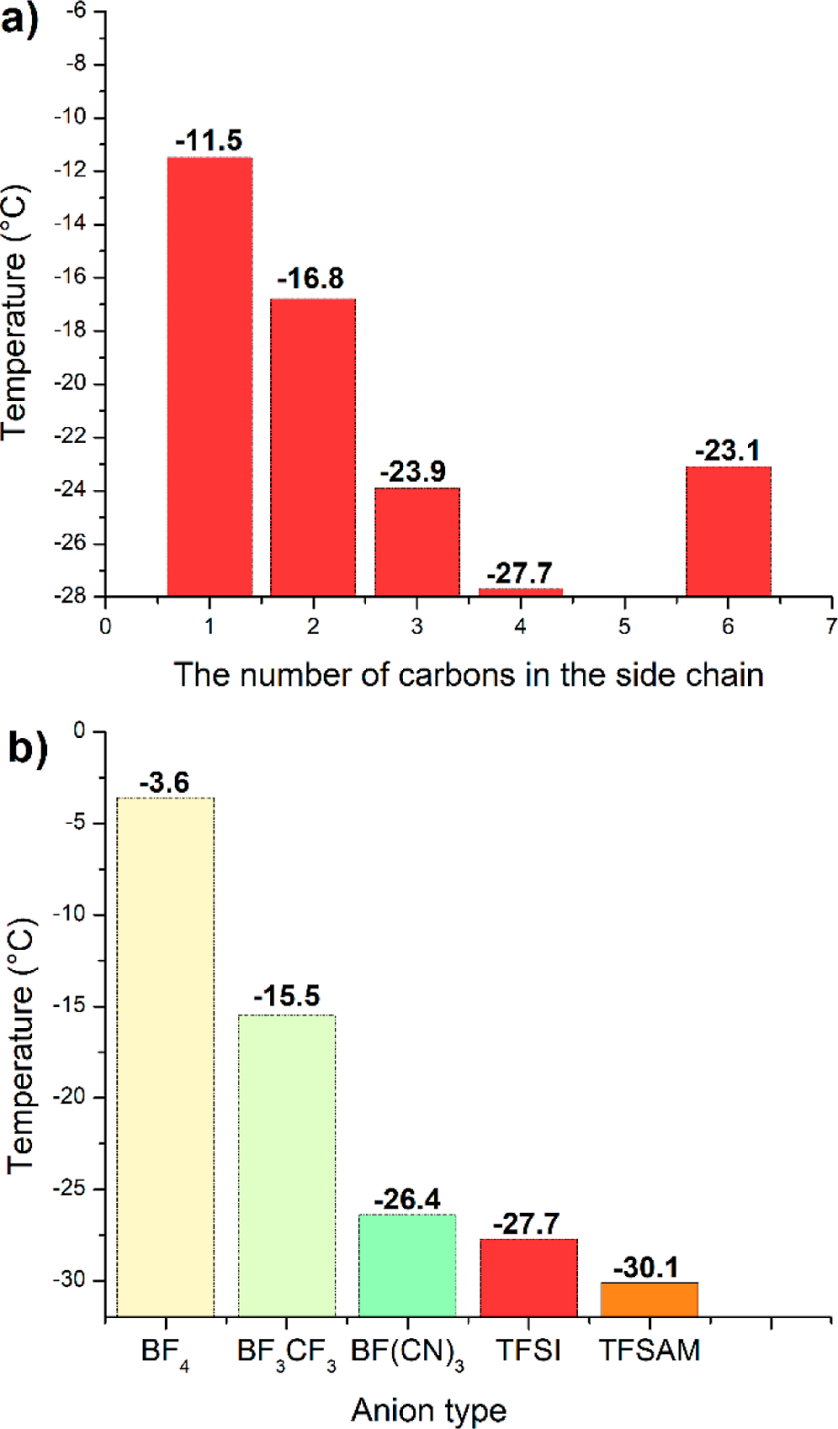
Glass transition temperature dependence on the structure for TFSI PILs with different alkyl substituents (a) and for *n*-butyl substituted PILs with various anions (b).

Further on, DSC was used to determine the glass transition temperature of PILs (Figures S13–S24 and Table 1). It should be noted that all polymers were observed to be cold-flowing viscoelastic liquids with glass-like transparency. Consistent with these observations, the DSC data collected (Figures S13–S24) confirm that all polymers are fully amorphous with a single *T*_g_. This assumption was additionally validated by WAXD analysis of the selected samples (Figure S25). The *T*_g_ values of TFSI-based PILs with alkyl side chains are shown on Figure 5. The observed trend displays a minimum, with the *T*_g_ of the PILs first decreasing with increasing alkyl side chain length up to *n* = 4 (*T*_*g*_ = −27.7 °C) for **PIL4TFSI** and then increasing at *n* = 6 (Figure 5). The same trend was observed previously for hexafluorophosphate imidazolium ionic liquids,^54^ with *T*_m_ decreasing until *n* = 6, at which point an increase was observed. This may be explained by improved chain packing and an associated increase in interchain interactions once the alkyl substituents are sufficiently long. The glass transition temperatures of all PILs with TFSI anions can be arranged in the following order:

*T*_g_ (°C): **PIL7(CF**_**3**_**)TFSI** (3.4) > **PIL8(SiOSi)TFSI** (−0.6) > **PIL6(Si(CH**_**3**_**)**_**3**_**)TFS**I (−9.2) > **PIL1(C**_**1**_**)TFSI** (−11.5) > **PIL2(C**_**2**_**)TFSI** (−16.8) > **PIL5(C**_**6**_**)TFSI** (−23.1) > **PIL3(C**_**3**_**)TFSI** (−23.9) > **PIL4(C**_**4**_**)TFSI** (−27.7 °C).

**PIL6TFSI**, **PIL7TFSI**, and **PIL8TFSI** with heteroatoms in the side chain demonstrated *T*_g_ values of −9.2, 3.4, and −0.6 °C, respectively. Thus, all PILs with side chains containing heteroatoms showed higher *T*_g_s in comparison with analogous PILs having alkyl substituents. Finally, among the studied TFSI-based PILs, **PIL7TFSI** with the fluorinated chain possesses the highest overall *T*_g_.

The influence of anion structure on PIL glass transition temperature was investigated by DSC and is represented below by the following order:

*T*_g_ (°C): **PIL4BF**_**4**_ (−3.6 °C) > **PIL4BF**_**3**_**CF**_**3**_ (−15.5 °C) > **PIL4BF(CN)**_**3**_ (−26.4 °C) ≈ **PIL4TFSI** (−27.7 °C) > **PIL4TFSAM** (−30.1 °C).

As stated above (see Section 3.4), the symmetry of the anion, its size, and interaction energy with the cation, as well as the charge delocalization, have a great impact on PILs *T*_g._ The transition from symmetric BF_4_ to asymmetric BF_3_CF_3_ and then to BF(CN)_3_ led to the decrease in *T*_g_ from −3.6 to −15.5 and −26.4 °C, respectively. A similar decrease of *T*_g_ can be seen by replacing the TFSI anion with the asymmetric TFSAM one.

### 3.6 PILs Electrochemical Properties

**Figure 6:**
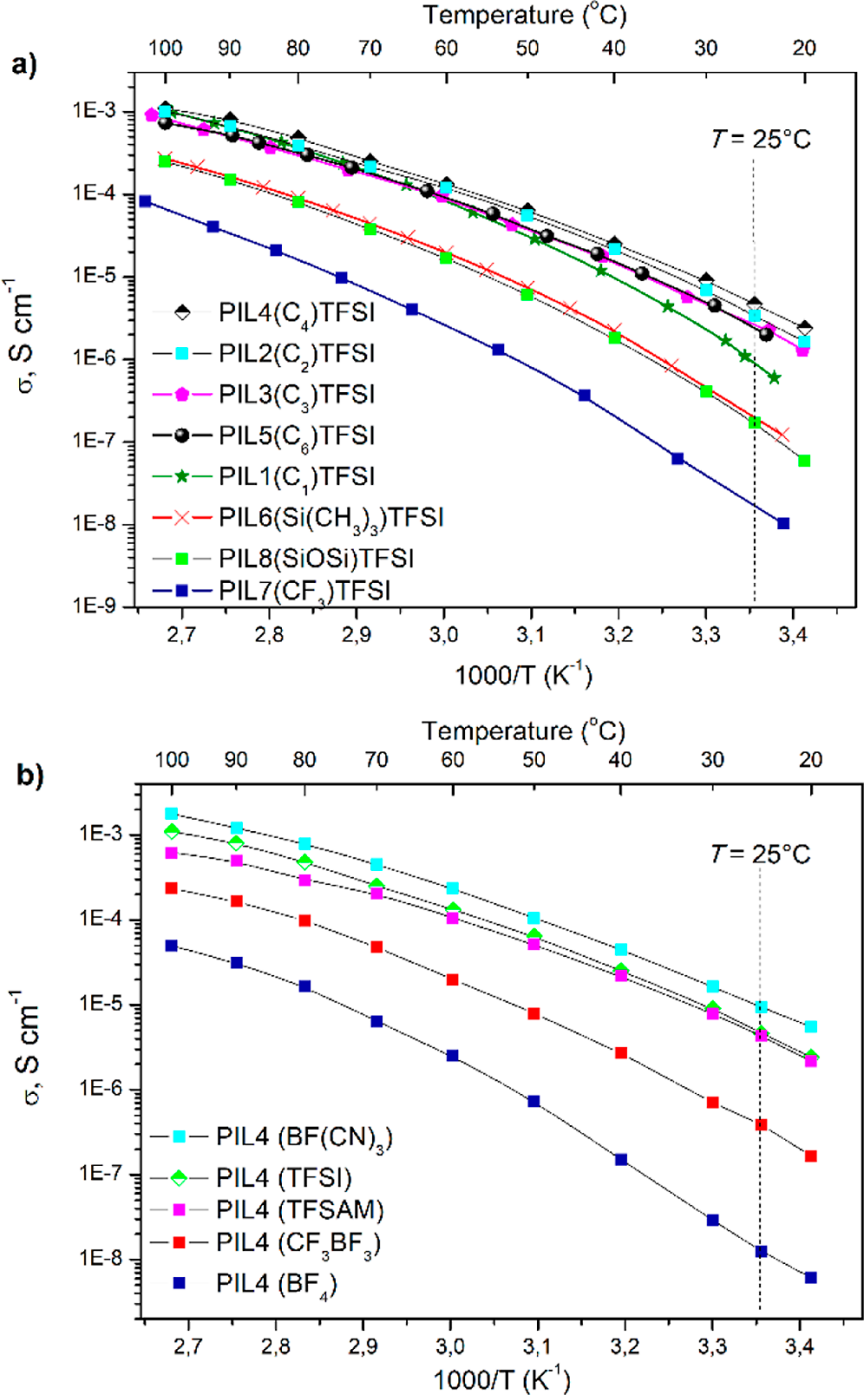
Temperature dependence of bulk ionic conductivity for TFSI PILs with different side chains (a) and for *n*-butyl-substituted PILs with various anions (b).

The ionic conductivity of PILs was measured over a wide temperature range using EIS (Figure 6). EIS data for all temperatures (Nyquist plots), as well as their linear fittings and equivalent circuit model fittings, are presented in Section XII and Figures S26 and S27 of the Supporting Information file. For all PIL, conductivity increased with increasing temperature; however, temperature dependence did not follow the linear Arrhenius behavior (Figure 6). This can be explained by the fact that anion diffusion occurs via two different mechanisms: (1) hopping of the anions between chemically bonded cations and (2) local segmental motion of polymer chains with coordinative oxyethylene fragments. In contrast, the temperature evolution of σ_DC_ followed a typical Vogel–Fulcher–Tammann (VFT) behavior for all studied PILs. The dependences were fitted with the VFT eq S6 and the fitting parameters are listed in Table S2 (see Section XII in the Supporting Information). The values obtained from the best fittings of the experimental curves (Figure 6) are in good accordance with those previously reported for other PILs.^55^

The determination of ionic conductivity at 25 °C revealed 3 orders of magnitude difference in σ_DC_ of PILs (Figure 6 and Table 1). This difference was significantly decreased with the increase in temperature.

For TFSI-based PILs, the conductivity values increased from 8.0 × 10^–8^ to 4.7 × 10^–6^ S cm^–1^ and can be ranked in the following decreasing order:

σ (25 °C, S cm^–1^): **PIL4(C**_**4**_**)TFSI** (4.7 × 10^–6^) > **PIL2(C**_**2**_**)TFSI** (3.3 × 10^–6^) > **PIL3(C**_**3**_**)TFSI** (2.6 × 10^–6^) > **PIL5(C**_**6**_**)TFSI** (2.4 × 10^–6^) > **PIL1(C**_**1**_**)TFSI** (8.4 × 10^–7^) > **PIL6(Si(CH**_**3**_**)**_**3**_**)TFSI** (2.0 × 10^–7^) > **PIL8(SiOSi)TFSI** (8.0 × 10^–8^) > **PIL7(CF**_**3**_**)TFSI** (1.6 × 10^–8^ S cm^–1^).

Within the whole temperature range, **PIL4TFSI** with the *n*-butyl side chain showed the highest conductivity values, while **PIL7TFSI** with the fluorinated chain demonstrated the lowest conductivity (Figure 6a). For polymers with alkyl substituents, the general trend of increasing conductivity with decreasing *T*_*g*_ is observed, with one exception—**PIL2TFSI** shows a higher than expected conductivity given its *T*_*g*_. Otherwise, **PIL4TFSI** with the lowest *T*_g_ exhibited the highest conductivity, while **PIL1TFSI**, with the highest *T*_g_ showed the lowest conductivity. Moreover, PILs with heteroatoms in the side chain **(PIL6TFSI–PIL8TFSI)** possessed 1 order of magnitude lower ionic conductivity in comparison with analogous PILs having alkyl substituents, which was found to be in correlation with their higher glass transition temperatures.

The effect of the counteranion’s structure on PIL ionic conductivity was even higher (Figure 6b). The conductivity trend for PILs with different anions at 25 °C is represented below:

σ (25 °C, S cm^–1^): **PIL4BF(CN)**_**3**_ (1.0 × 10^–5^) > **PIL4TFSI** (4.7 × 10^–6^) ≈ **PIL4TFSAM** (4.3 × 10^–6^) > **PIL4BF**_**3**_**CF**_**3**_ (3.9 × 10^–7^) > **PIL4BF**_**4**_ (1.2 × 10^–8^ S cm^–1^).

The transfer from symmetric BF_4_ anion to asymmetric BF_3_CF_3_ and BF(CN)_3_ anion resulted in nearly 3 orders of magnitude increase in ionic conductivity from 1.2× 10^–8^ to 1.0 × 10^–5^ S cm^–1^ (25 °C). On the contrary, the introduction of a smaller and asymmetric TFSAM anion practically does not affect the ionic conductivity of **PIL4TFSAM** in comparison with **PIL4TFSI**, which was in agreement with the work published previously by Drockenmuller et al.^32^ It can be concluded that among PILs synthesized in the current study, **PIL4TFSI** and **PIL4BF(CN)**_**3**_ demonstrated the highest ionic conductivities. It is worth noting that the value of 1.0 × 10^–5^ S cm^–1^ found for **PIL4BF(CN)**_**3**_ at 25 °C can be ranked among the top 15 PILs with the highest conductivities published to date (Table S3).

**Figure 7:**
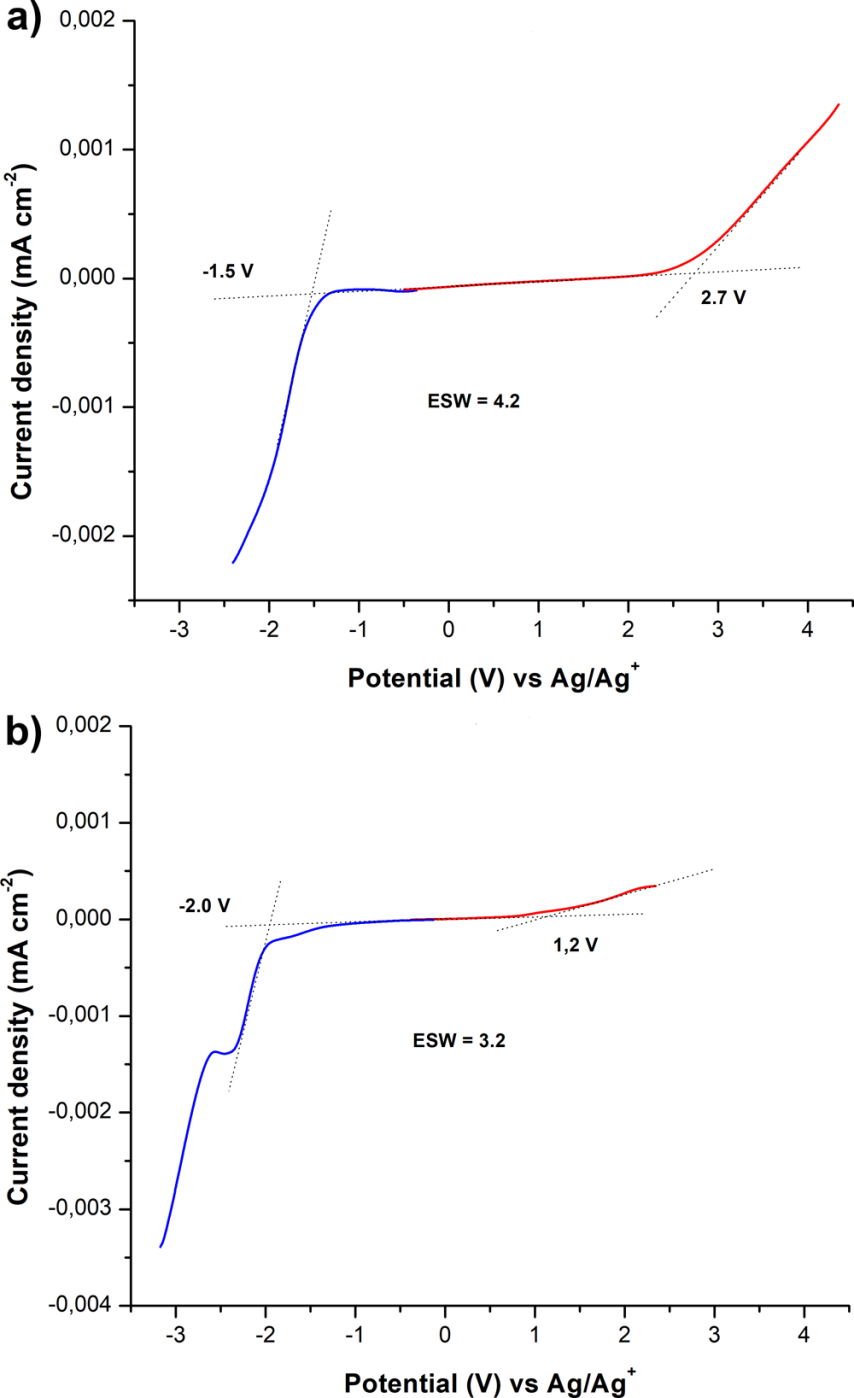
Electrochemical stability window for **PIL4TFSI** (a) and **PIL4BF(CN)**_**3**_ (b) at 25 °C (Pt foils as the working and counter electrodes and Ag mesh as the reference electrode, scan rate of 5 mV s^–1^).

Lastly, the electrochemical stability limits of PILs with the highest ionic conductivity were assessed via cyclic voltammetry (CV). Figure 7 shows the anodic and cathodic scans of **PIL4TFSI** and **PIL4BF(CN)**_**3**_ at 25 °C. The oxidation potential for **PIL4TFSI** against a platinum electrode was found to be higher than that of **PIL4BF(CN)**_**3**_, reaching a value of 2.7 V vs Ag^+^/Ag. On the contrary, the reduction potential of **PIL4TFSI** was lower than of **PIL4BF(CN)**_**3**_: −1.5 vs −2.0 V, respectively (Figure 7). For **PIL4BF(CN)**_**3**_, an additional irreversible peak was found at −2.4 V, which can be attributed to the oxidation processes of carbon atoms in the anion. The overall evolution of the electrochemical stability of PILs can be summarized as follows: ESW **PIL4TFSI** (4.2 V) > ESW **PIL4BF(CN)**_**3**_ (3.2 V). At this juncture, the TFSI anions show higher electrochemical stability than the tricyano fluoroborate one. Additionally, the electrochemical stability vs Li^+^/Li was studied at 70 °C for **PIL4TFSI** filled with 10 wt % of LiTFSI (Figure S29). Well-defined reversible lithium plating/stripping processes were clearly observed in the form of two reduction/oxidation peaks between −0.4 and 0.3 V vs Li^+^/Li, which confirm the efficient transfer of lithium ions through the polymer network and at the polymer electrolyte/electrode interface. The oxidation stability of the **PIL4TFSI/LiTFSI** solid electrolyte was studied during the anodic scan and was determined as 4.8 V vs Li^+^/Li, which indicates the high potential of the prepared polyelectrolytes to be used in Li batteries as well.

## 4 Conclusions

A series of twelve cationic PILs were synthesized by simple modification of the commercially available based on the poly(epichlorohydrin-*co*-ethylene oxide) copolymer with various N-substituted imidazoles and further ion metathesis reactions. The influence of the side chain and anion structures on the properties of the resultant PILs was studied in detail. It was found that the presence of heteroatoms in the side chains containing silyl, siloxane, and perfluorinated fragments led to the increase in glass transition temperatures and a decrease in ionic conductivity of PILs in comparison with analogs bearing alkyl chains. Among PILs with alkyl side chains, a minimum in the dependence of the *T*_g_ on the number of carbon atoms in the imidazolium substitutes has been determined, with an inverse relationship observed vs ionic conductivity in all but one case. In particular, PIL with *n*-butyl side chains gave the lowest *T*_g_ (−25.4 °C) and the highest ionic conductivity (4.7 × 10^–6^ S cm^–1^ at 25 °C) among the studied polyelectrolytes with TFSI anions.

The best performing polyelectrolyte with *n*-butyl substituents was further investigated with respect to the influence of the counteranion. For the first time, PILs with BF_3_CF_3_ and BF(CN)_3_ anions were synthesized and compared to polyelectrolytes with BF_4_, TFSI anions, and TFSAM anions. The introduction of the asymmetry into the anion structure allowed for further increases in ionic conductivity up to 1.0 × 10^–5^ S cm^–1^ at 25 °C for **PIL4BF(CN)**_**3**_, which is among the top 15 most conductive PILs published to date (Table S1). Such results highlight the promise of this class of materials as enabling technologies for the fabrication of future solid-state electrochemical devices with enhanced safety and performance.

## Supporting Information Available

Full details of poly(epichlorohydrin-*co*-ethylene oxide) and methods used in the study, ^1^H, ^13^C, ^19^F, ^11^B NMR, and IR spectra of the compounds, DSC and GPC plots, EIS data for all temperatures (Nyquist plots) as well as their linear fittings, equivalent circuit model fittings, BDS and WAXD plots, summary of the PILs with the highest ionic conductivity published to date, and determination of the quaternization degree and CV of PIL4TFSI with 10% of LiTFSI (PIL4TFSi/LiTFSI) vs Li/Li^+^ at 70 °C (PDF)
