# Supplementary material for: Design of Highly Conductive PILs by Simple Modification of Poly(epichlorohydrin-co-ethylene oxide) with Monosubstituted Imidazoles
Source: ACS Polym Au. 2024 Sep 12;4(6):512–26. doi: 10.1021/acspolymersau.4c00051 (PMC11638787; doi:10.1021/acspolymersau.4c00051)
Supplement: Supplementary file 1 — lg4c00051_si_001.pdf [file lg4c00051_si_001.pdf]

Supplementary Information for

Design of highly conductive PILs by simple modification of poly(epichlorohydrin-co-ethylene oxide) with monosubstituted imidazoles

Daniil R. Nosov [1,2], Elena I. Lozinskaya [3], Dmitrii Y. Antonov [3], Denis O. Ponkratov [3], Andrey A. Tyutyunov [3], Malak Alaa Eddine [4], Cédric Plesse [5], Daniel F. Schmidt [1*] and Alexander S. Shaplov [1,*]

[1] Luxembourg Institute of Science and Technology (LIST), 5 avenue des Hauts-Fourneaux, L-4362 Esch-sur-Alzette, Luxembourg

[2] Department of Physics and Materials Science, University of Luxembourg, 2 Avenue de l'Université, L-4365 Esch-sur-Alzette, Luxembourg

[3] A.N. Nesmeyanov Institute of Organoelement Compounds, Russian Academy of Sciences (INEOS RAS), Vavilov street 28, 119991, GSP-1, Moscow, Russia

[4] Univ Lyon, Université Lyon 1, CNRS, Ingénierie des Matériaux Polymères, UMR 5223, F-69003, Lyon, France

[5] CY Cergy Paris Université, Laboratoire de physicochimie des polymères et des interfaces (LPPI), 5 mail Gay Lussac, F-95031 Cergy-Pontoise Cedex, France

[*] Correspondence: daniel.schmidt@list.lu (D.S); alexander.shaplov@list.lu (A.S.), Tel. +352 2758884579 (A.S.).

Contents

I. Poly(epichlorohydrin-co-ethylene oxide) 3

II. Methods 3

III. Determination of the quaternization degree (Q) 5

Table S1. Quaternization degrees determined for PILs via ^{1}H NMR. 6

IV. Spectroscopic analysis 7

V. Gel permeation chromatography 16

VI. DSC plots of polymers 18

VII. Representative WAXD plots for qualitative analysis 24

VIII. Electrochemical Impedance Spectroscopy (EIS) 25

Table S2. Parameters obtained by VFT fits of the EIS data. 28

IX. Broadband dielectric spectroscopy (BDS) 29

X. Cyclic voltammetry (CV) of PIL4TFSI vs. Li/Li$^+$ at 70 °C 31

Table S3. Comparison of the ionic conductivity data for the selected PILs reported in literature and for those obtained in the present work (updated on May, 2024). 33

I. Poly(epichlorohydrin-co-ethylene oxide)

ECO **Hydrin®** **EPICHLOROHYDRIN RUBBER**

Grade	Chlorine Content [wt %]	Mooney Viscosity ML 1+4, 100°C [MU]	Relative Density	Tg [°C]	Main Characteristics & Applications
**Hydrin® C2000**	26	90 – 102	1,28	– 41	Copolymer. Used for fuel pump diaphrams, hoses, tubes, coated fabrics, and vibration mounts.
**Hydrin® C2000L**	26	65 – 75	1,28	– 41	Copolymer grades with low viscosity.
**Hydrin® C2000LL**	26	53 – 65	1,28	– 41	
**Hydrin® C2000XL**	26	40 – 52	1,28	– 41	

Poly(epichlorohydrin-co-ethylene oxide) (Hydrin® C2000XL, Zeon Europe GmbH), m:n = 1:1, [M_w = 8.73 $\times$ 10^6 g/mol, M_w/M_n = 4.5 by GPC in THF at 30°C, calibration with PMMA standards], [M_w = 2.41 $\times$ 10^6 g/mol, M_n = 1.10 $\times$ 10^5 g/mol, M_w/M_n = 21.8 by GPC in 0.1 M LiTFSI in DMF at 50 °C, calibration with PMMA standards] was used as received.

II. Methods

NMR spectra were recorded on AMX-400 or Avance III™ HD 600 MHz (Bruker, Germany) spectrometer at 25°C in the indicated deuterated solvent and are listed in ppm. The signals corresponding to the residual protons and carbons of the deuterated solvent were used as an internal standard for ^{1}H and ^{13}C NMR, respectively. The C_6F_6 (−164.9 ppm) and $F_3B{\cdot}OEt_2$ were used as an external standard for ^{19}F and ^{11}B NMR, respectively. IR spectra were acquired on a Magna-750 (Nicolet Instrument Corporation, USA) or on INVENIO R (Brucker, Germany) Fourier IR-spectrometer using KBr pellets or ATR technology (128 scans, resolution is 4 cm^{-1}) and Spectragryph optical spectroscopy software [1].

Thermal gravimetric analysis (TGA) was carried out in air on a TGA2 STARe System (Mettler Toledo, Switzerland) applying a heating rate of 5 °C min^{-1}. The onset weight loss temperature (T_{onset}) was determined as the point in the TGA curve at which a significant deviation from the horizontal was observed. The resulting temperature was then rounded to the nearest 5°C. For Differential Scanning Calorimetry (DSC) measurements all samples were hermetically sealed in Al pans inside the argon-filled glove-box (MBRAUN MB-Labstar, H_2O and O_2 content < 0.5 ppm). DSC was performed on a DSC 300 Caliris® Select (Netzsch, Germany) differential calorimeter applying a heating rate of 5°C min^{-1} in the range of -80 to

100 °C. Two heating-cooling cycles were carried out for each sample. Glass transition temperatures (T_g) were calculated from the second heating curve by the shift of the baseline of the DSC curve.

Ionic conductivity (σ_{DC}) was measured by electrochemical impedance spectroscopy (EIS) with a VSP potentiostat/galvanostat (Bio-Logic Science Instruments, France). To avoid any influence of moisture/ humidity on the conductivity of polymer electrolytes, the latter were preliminary dried at 80 °C/0.1 mbar for 12 h in the B-585 oven (Buchi Glass Drying Oven, Switzerland) filled with P_2O_5 and were transferred under vacuum inside an argon-filled glovebox (MBRAUN MB-Labstar, H_2O and O_2 content<0.5 ppm). The samples were sandwiched between two stainless steel electrodes. The distance between the electrodes was kept equal to 250 μm using a Teflon spacer ring with an inner area of 0.50 cm^2. Symmetrical stainless steel/**PIL**/stainless steel assembly was clamped into the 2032-coin cell and afterwards was taken out from the glovebox. EIS experiments were carried by applying a 10 mV perturbation in the frequency range from 10^{-2} to 2×10^5 Hz and in a temperature range from 20 to 100 °C. Temperature was controlled using the programmed M-53 oven (Binder, Germany), where cells were allowed to reach thermal equilibrium for at least 45 min before each test.

Cyclic voltammetry (CV) was used to determine the electrochemical stability window (ESW) of PILs at 25°C. The ESW was studied under an argon atmosphere in a glovebox (MBRAUN MB-Labstar, H_2O and O_2 content <0.5 ppm) at room temperature using a VSP potentiostat/galvanostat (Bio-Logic Science Instruments, France). The three-electrode cells were assembled by sandwiching the polymer sample between two Pt flat electrodes (used as working and counter electrodes) and silver mesh (used as pseudo-reference electrode) to form the following architecture: Pt/coPIL/Ag mesh/coPIL/Pt. The ECW test was performed by scanning at 5 mV s^{-1} rate from the open circuit potential (OCV) toward positive or negative potentials.

Wide Angle X-ray Diffraction (WAXD) was performed on X-Ray difractometer D8 Discover (Bruker). The following conditions of analysis were applied: 2θ range: 5°<2θ<70°, measurement time per step: 197s, step size 0.026°. Primary optics: focusing Mirror, Soller slit: 0.04 rad, mask: 10 mm, divergence slit: 1/2°, antiscatter slit: 1/2°. Secondary optics: 1-D mode, programmable antiscatter slit: fixed slit of 0.5°, Soller slit: 0.04 rad, large Ni filter, PIXcel3D (1D detection mode).

III. Determination of the quaternization degree (Q)

The quaternization degree **Q** was determined by ^{1}H NMR using integral in the region of 4.10-3.40 ppm and the following equations:

$$Q = \frac{I^n_{PIL4Cl}}{I^n_{PIL4Cl}+I^n_{Hydrin}} \times 100\% \quad \text{(eq. S1)}$$

$$I^n_{PIL4Cl} = \frac{I^{theor}_{PIL4Cl}}{n_{PIL4Cl}} = \frac{7}{7} = 1 \quad \text{(eq. S2)}$$

$$I^n_{Hydrin} = \frac{I_{Hydrin}}{n_{Hydrin}} = \frac{I^{exp}_{PIL4Cl}-I^{theor}_{PIL4Cl}}{n_{Hydrin}} = \frac{7.46-7.0}{9} = 0.051 \quad \text{(eq. S3)}$$

$$Q = \frac{1}{1+0.051} \times 100\% = 95\% \quad \text{(eq. S4)}$$

, where I^n_{PIL4Cl} is the normalized integral for quaternized polymer PIL4Cl in the region of 4.10-3.40 ppm, I^n_{Hydrin} is the normalized integral for unmodified Hydrin polymer in the region of 4.10-3.40 ppm, I^{theor}_{PIL4Cl} is the theoretical integral for fully quaternized polymer PIL4Cl in the region of 4.10-3.40 ppm, n_{PIL4Cl} is the theoretical number of protons corresponding to fully quaternized polymer PIL4Cl in region of 4.10-3.40 ppm (Figure S1b, signals 1-4), I_{Hydrin} is the integral for unmodified Hydrin in the region of 4.10-3.40 ppm, n_{Hydrin} is the theoretical number of protons corresponding to unmodified Hydrin in region of 4.10-3.40 ppm (Figure S1a, signals 1-5), I^{exp}_{PIL4Cl} is the experimentally determined integral for fully quaternized polymer PIL4Cl in the region of 4.10-3.40 ppm.

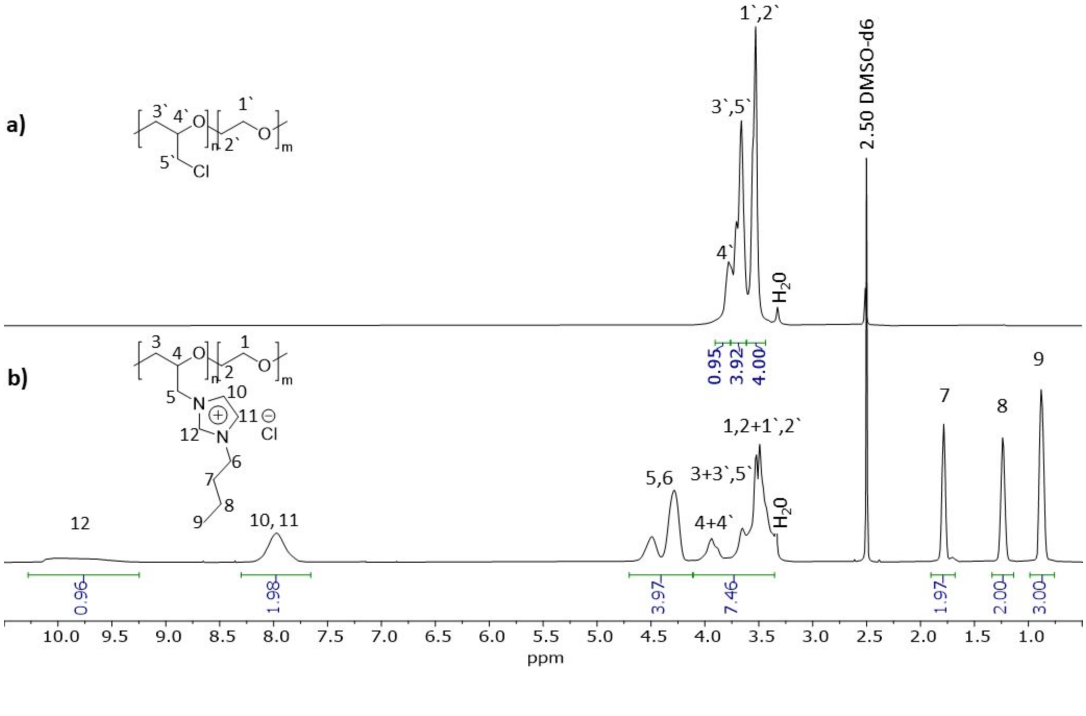

Figure S1. Comparison of ^{1}H NMR of poly(epichlorohydrin-co-ethylene oxide) (a) and poly(1-butyl-3-[oxiran-2-ylmethyl]-1-imidazole-3-ium-co-ethylene oxide) chloride (b).

Table S1. Quaternization degrees determined for PILs via ^{1}H NMR.

**Sample**	**Quaternization degree, Q (%)**
**PIL1Cl**	**94**
**PIL2Cl**	**96**
**PIL3Cl**	**97**
**PIL4Cl**	**95**
**PIL5Cl**	**95**
**PIL6Cl**	**96**
**PIL7Cl**	**90**
**PIL8Cl**	**60**

IV. Spectroscopic analysis

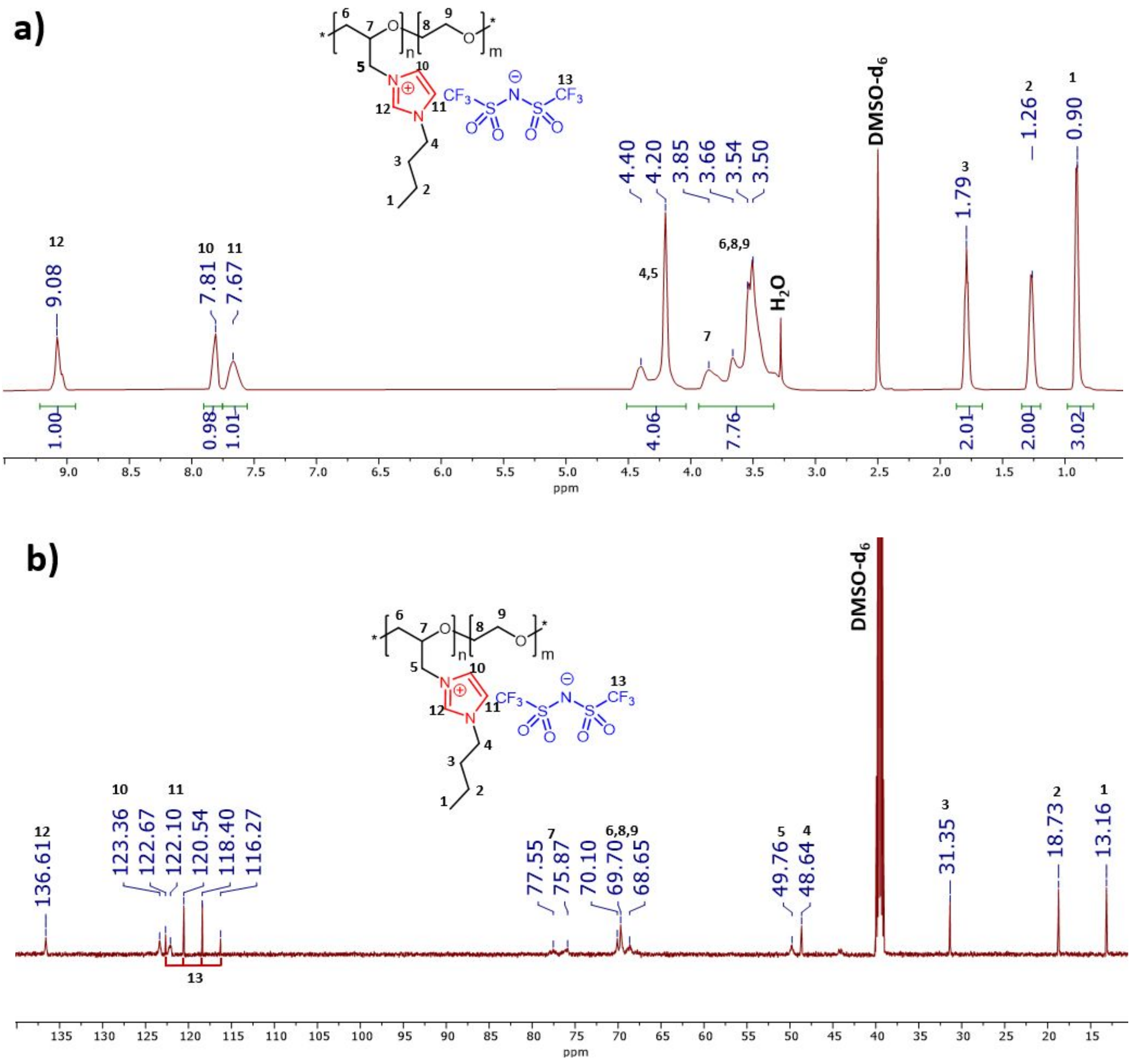

Figure S2. ^{1}H NMR (a), ^{13}C NMR (b) of **PIL4TFSI** (25 °C, DMSO- d_6).

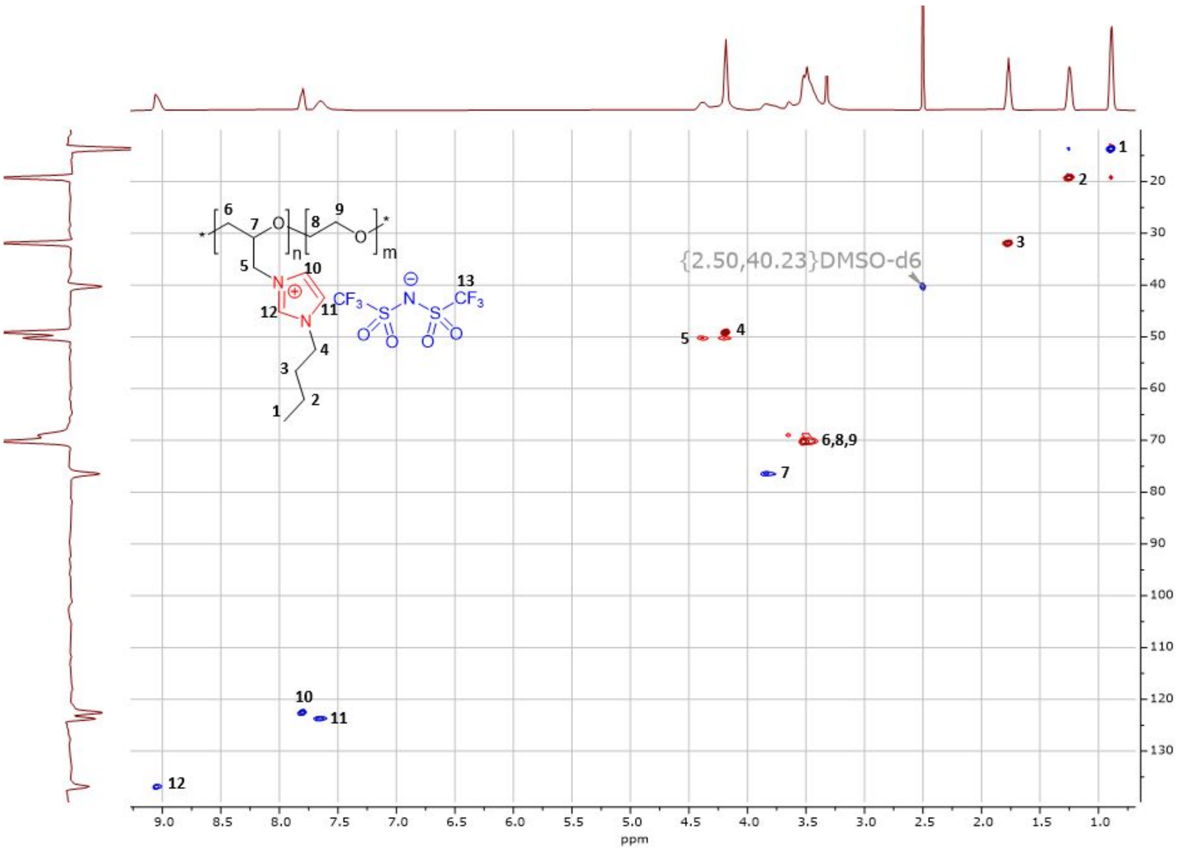

Figure S3. HSQC of **PIL4TFSI** (25 °C, DMSO- d_6).

a)

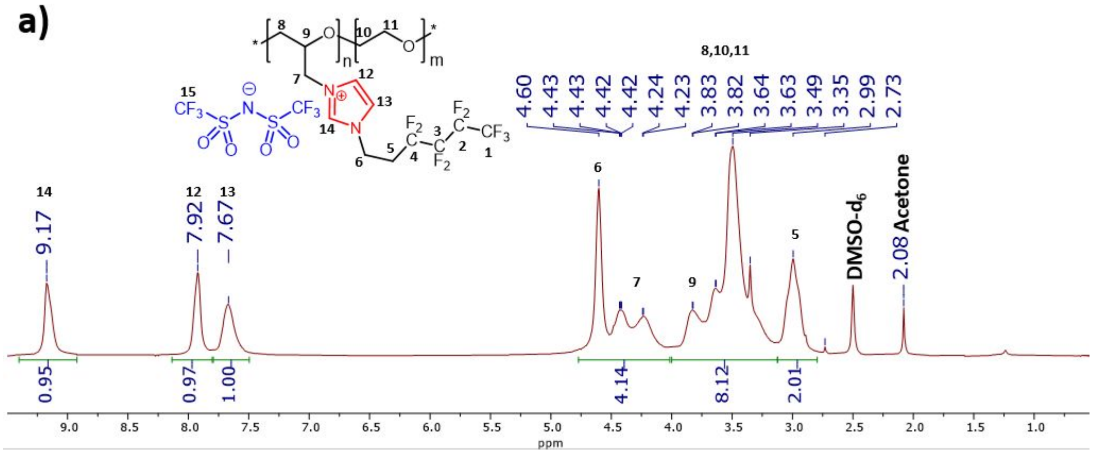

b)

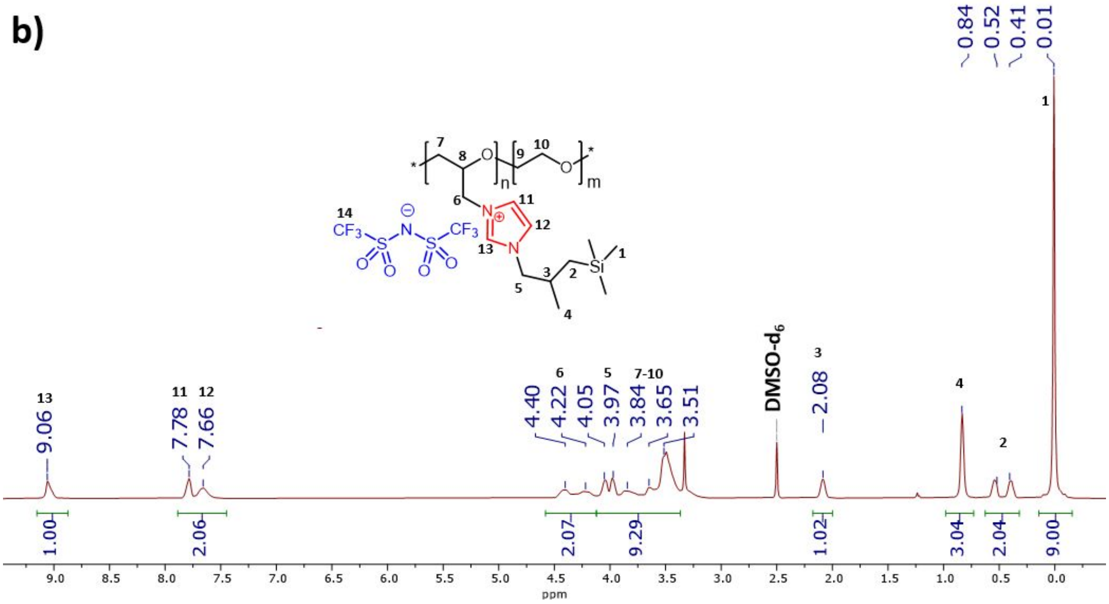

c)

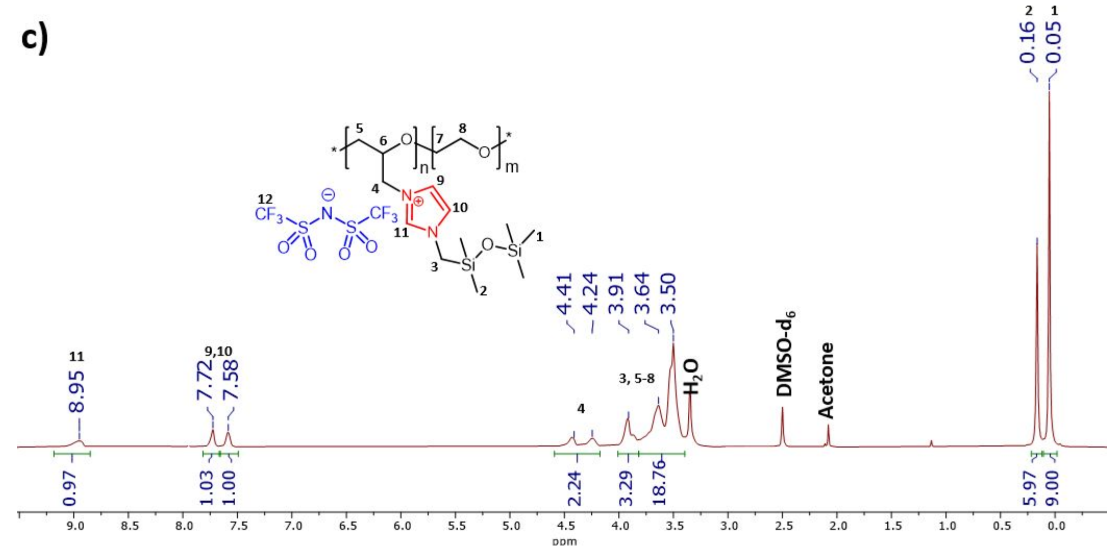

Figure S4. ^{1}H NMR of polymers **PIL7TFSI** (a), **PIL6TFSI** (b), **PIL8TFSI** (c) (25 °C, DMSO-d_6).

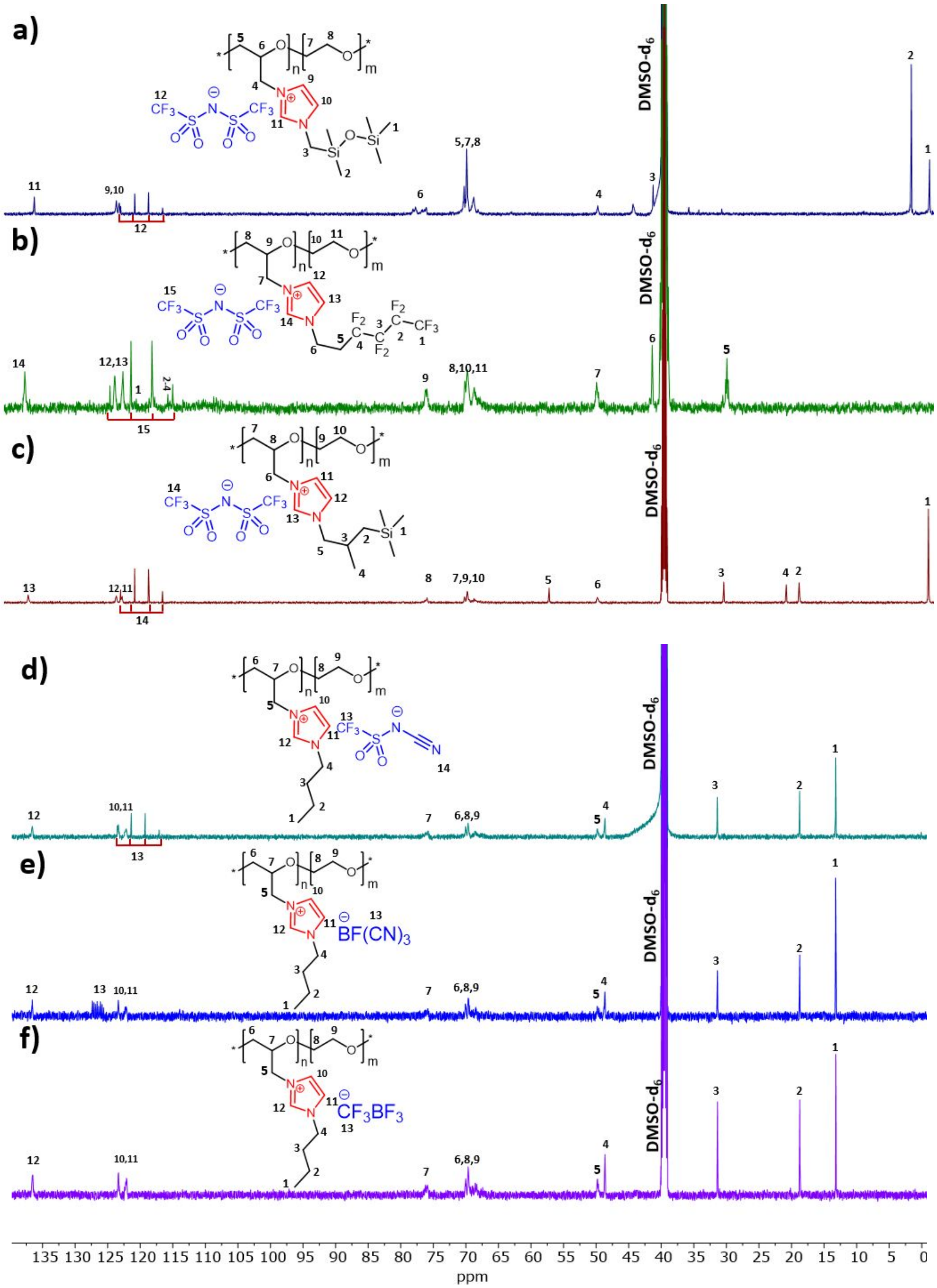

Figure S5. ^{13}C NMR of polymers **PIL8TFSI** (a), **PIL7TFSI** (b), **PIL6TFSI** (c), **PIL4TFSAM** (d), **PIL4BF(CN)$_3$** (e) and **PIL4CF$_3$BF$_3$** (f) (25 °C, DMSO-d$_6$).

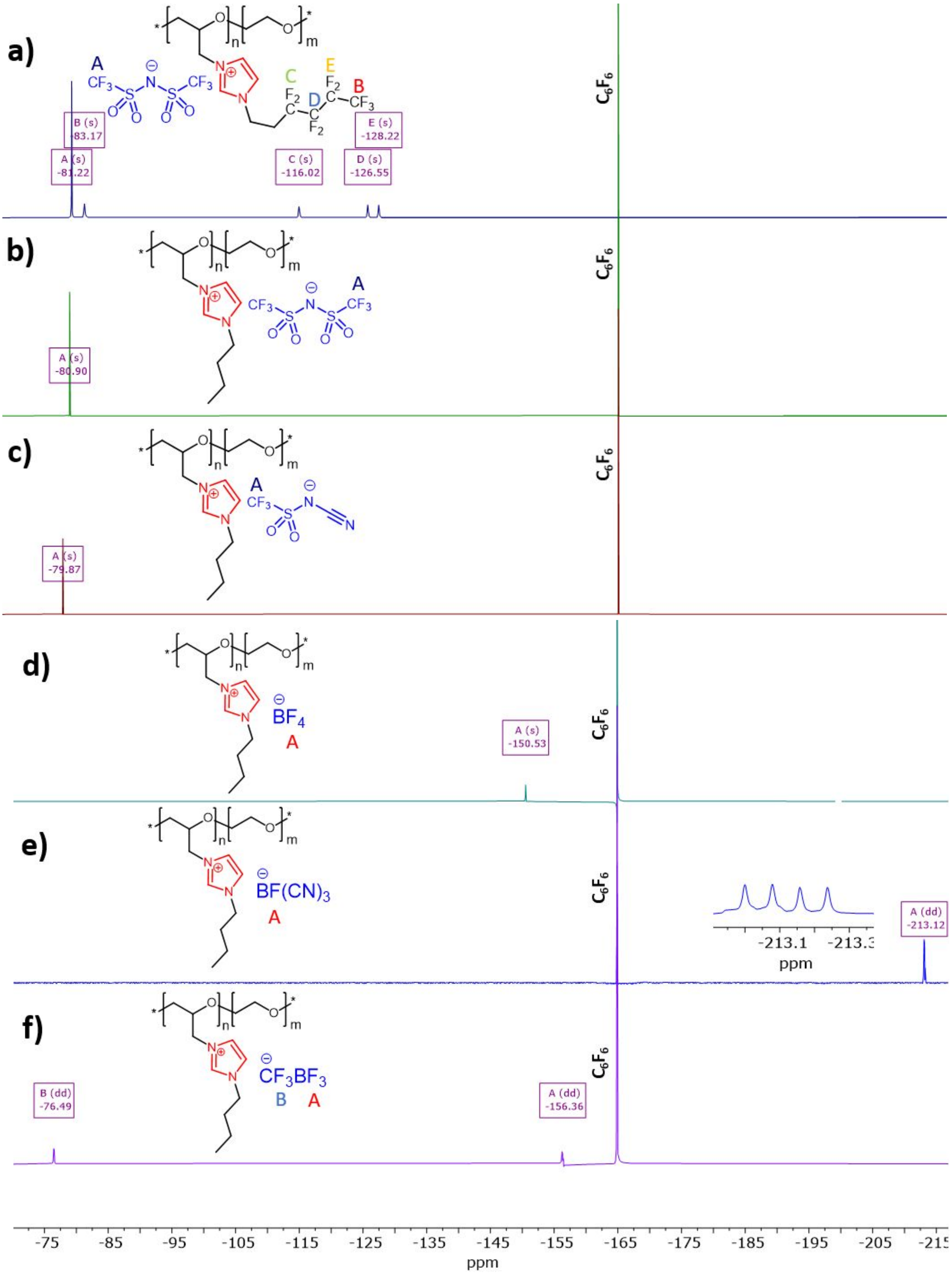

Figure S6. ^{19}F NMR of polymers **PIL7TFSI** (a), **PIL4TFSI** (b), **PIL4TFSAM** (c), **PIL4BF$_4$** (d), **PIL4BF(CN)$_3$** (e) and **PIL4CF$_3$BF$_3$** (f) (25 °C, DMSO-d$_6$).

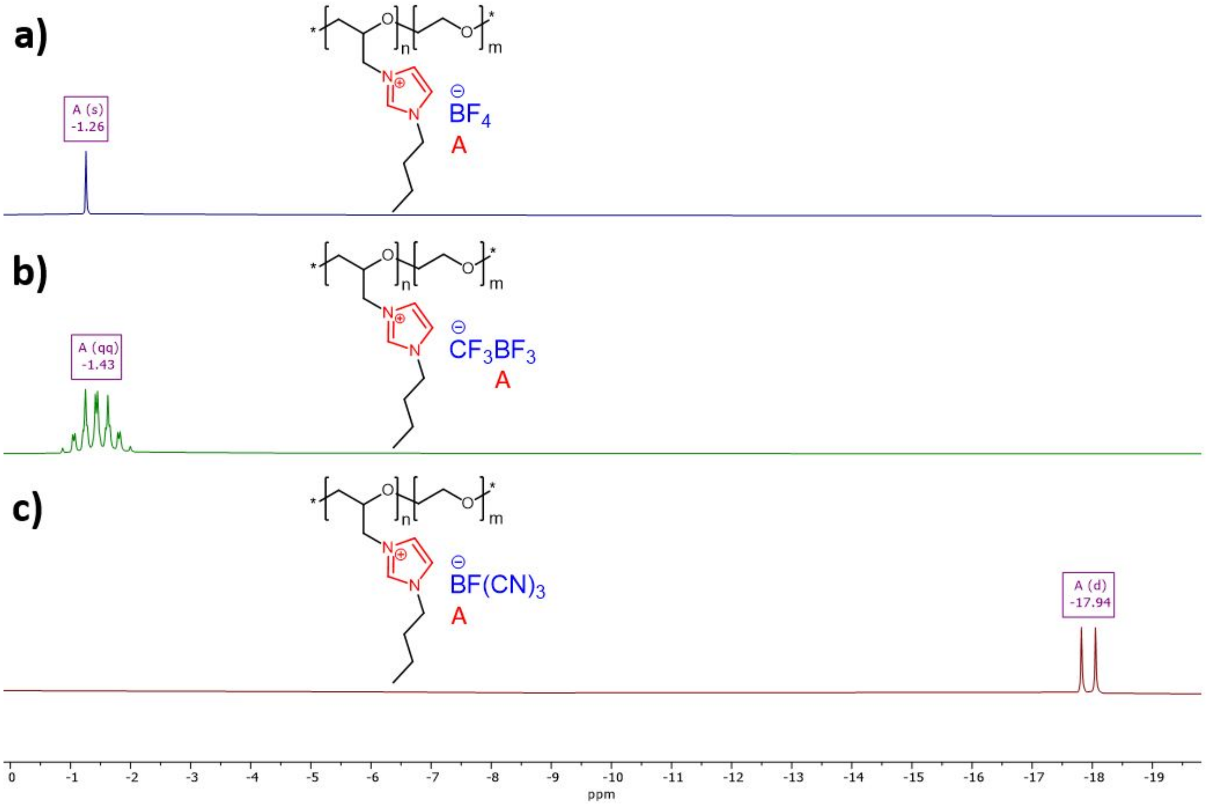

Figure S7. ^{11}B NMR of polymers **PIL4BF$_4$** (a), **PIL4CF$_3$BF$_3$** (b), **PIL4BF(CN)$_3$** (c), (25 °C, DMSO-d$_6$).

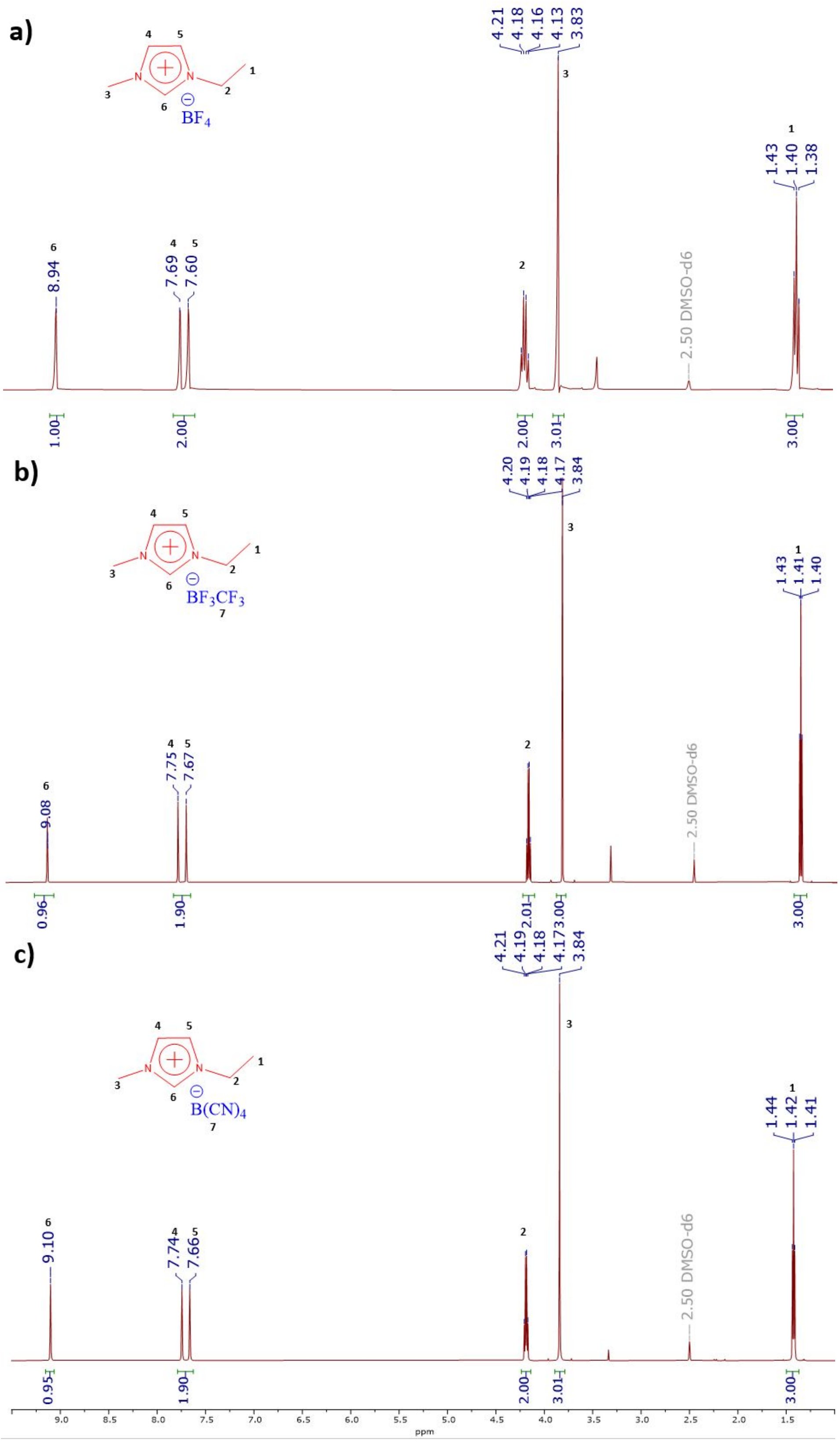

Figure S8. ^{1}H NMR of polymers 1-ethyl-3-methylimidazolium tetrafluoroborate (a), 1-ethyl-3-methylimidazolium trifluoro(trifluoromethyl)borate (b), 1-ethyl-3-methylimidazolium tetracyanoborate (c), (25 °C, DMSO-d_6).

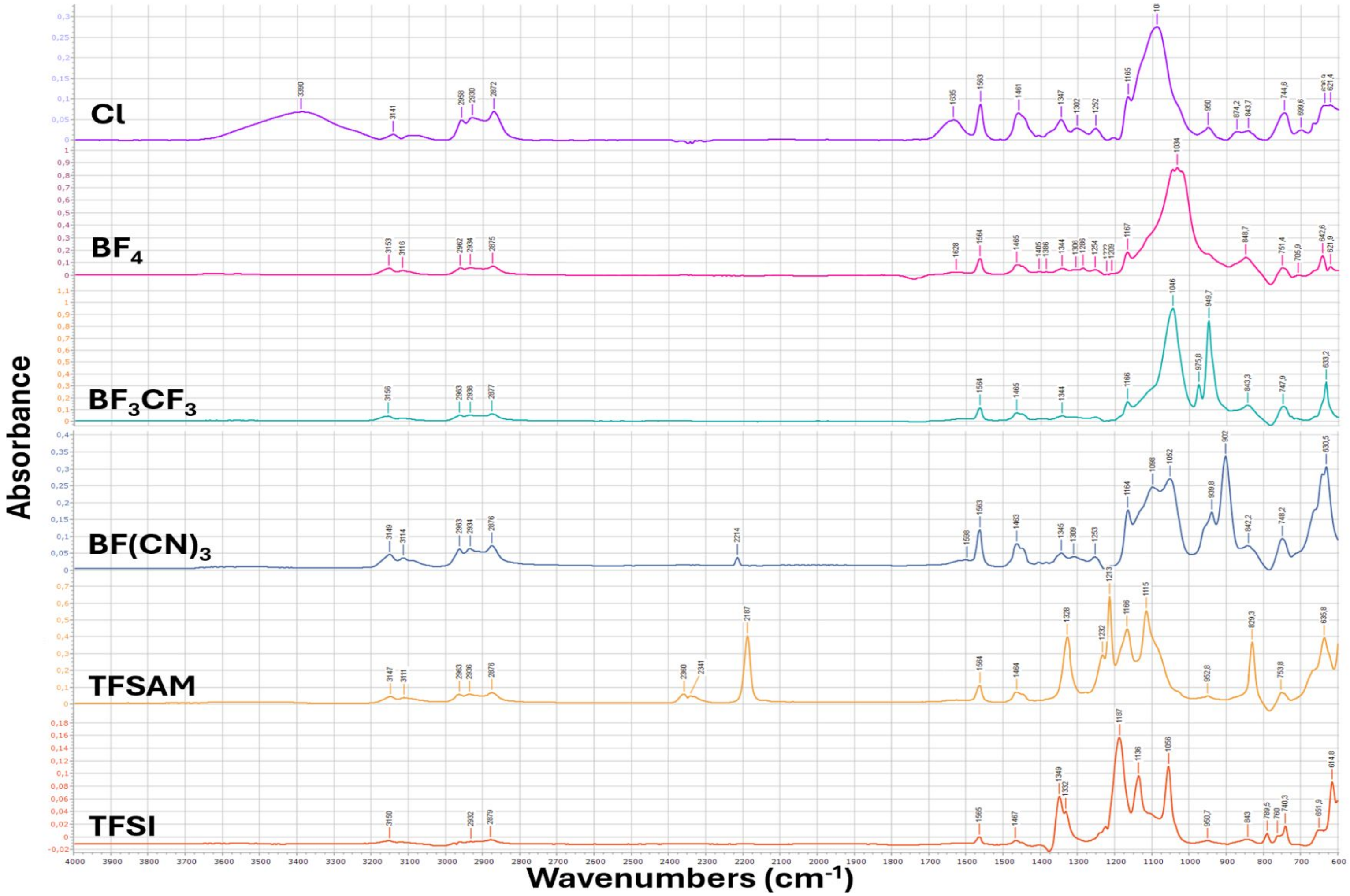

Figure S9. IR spectra of n-butyl substituted PILs with different anions: **PIL4Cl** (violet), **PIL4BF$_4$** (magenta), **PIL4CF$_3$BF$_3$** (green), **PIL4BF(CN)$_3$**

(blue), **PIL4TFSAM** (yellow) and **PIL4TFSI** (orange).

V. Gel permeation chromatography

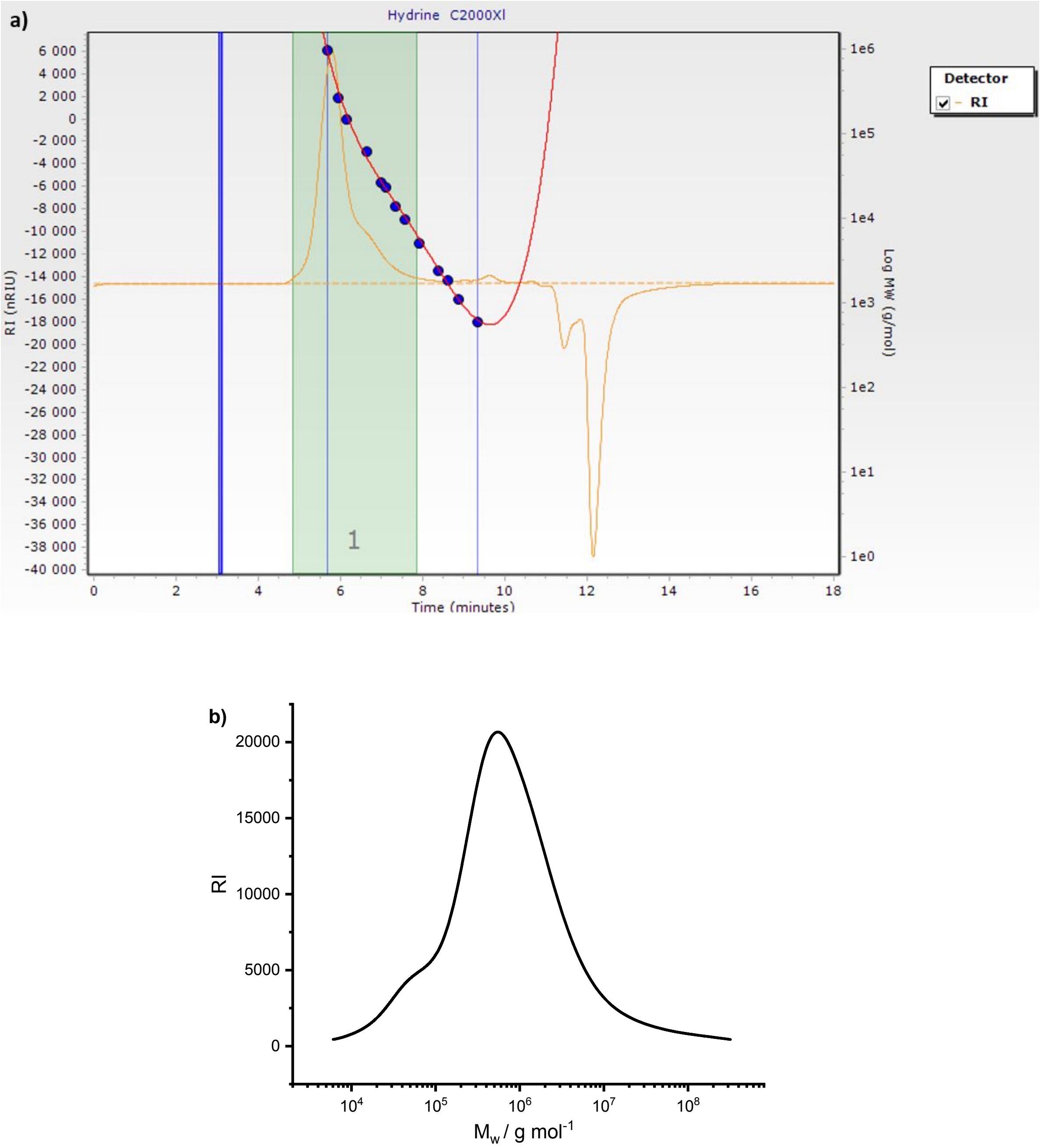

Figure S10. SEC traces of poly(epiclorohydrin-co-ethylene oxide) Hydrin® C2000XL. M_w = 2.41 $\times$ 10^6 g/mol, M_n = 1.10 $\times$ 10^5 g/mol, M_w/M_n = 21.8 (50 °C, 0.1 M LiTFSI in DMF, calibration with PMMA standards).

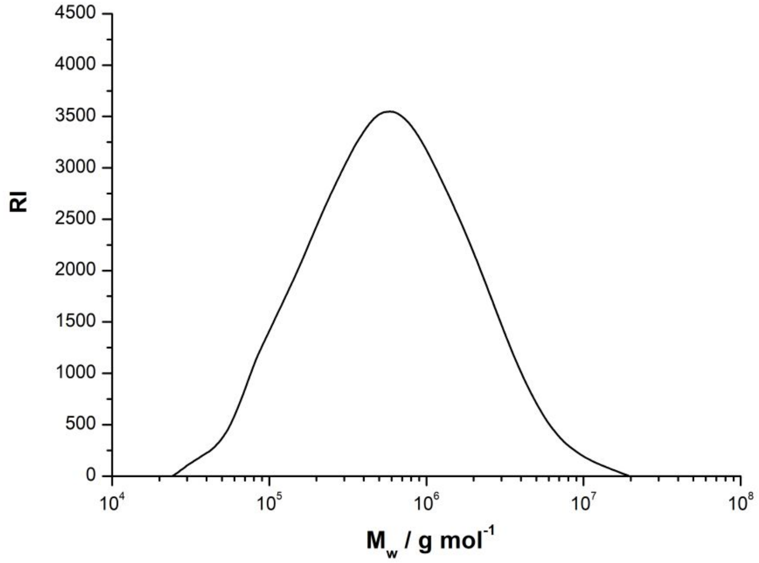

Figure S11. SEC trace of poly(epiclorohydrin-co-ethylene oxide) Hydrin® C2000XL. $M_w = 8.73 \times 10^6$ g/mol, $M_w/M_n = 4.5$ (30 °C, THF, calibration with PMMA standards).

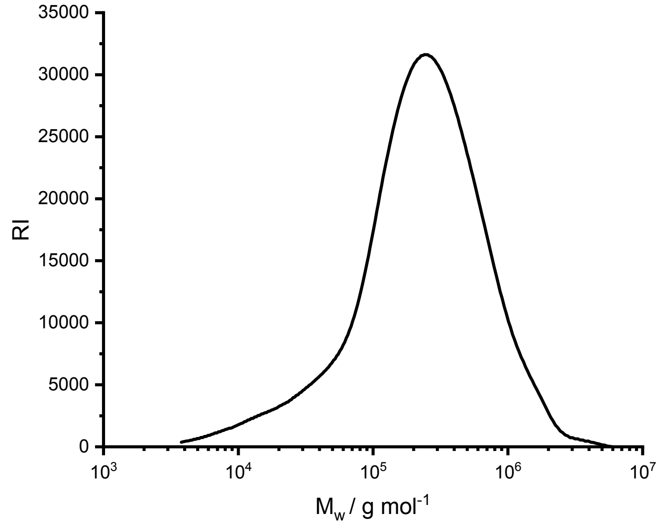

Figure S12. SEC trace of **PIL4TFSI**. $M_w = 3.41 \times 10^5$ g/mol, $M_n = 9.82 \times 10^4$ g/mol, $M_w/M_n = 3.5$ (50 °C, 0.1 M LiTFSI in DMF, calibration with PMMA standards).

VI. DSC plots of polymers

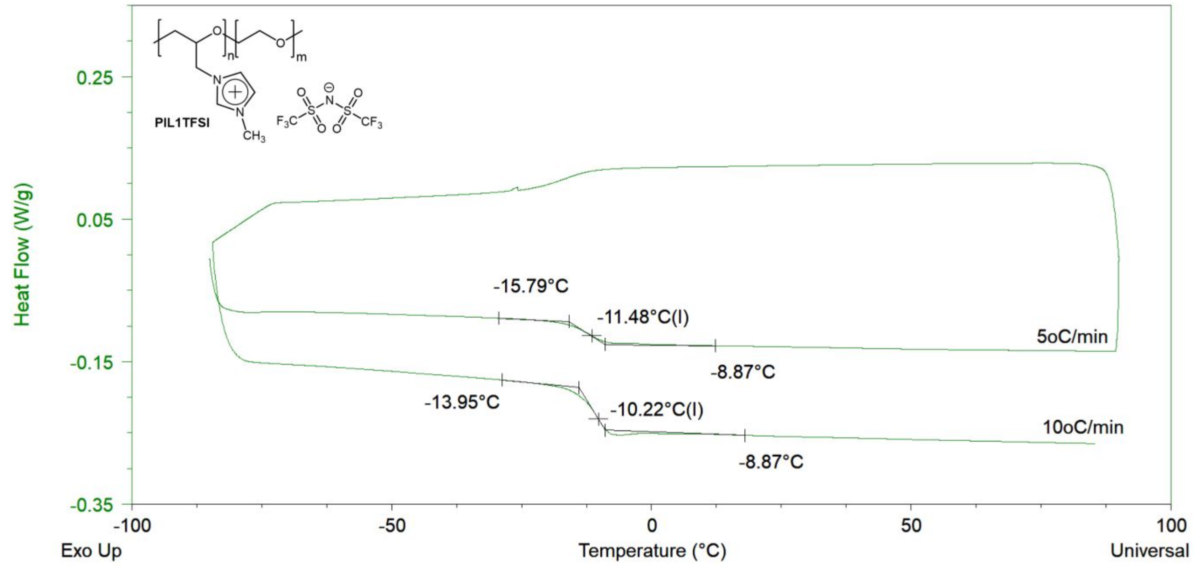

Figure S13. DSC plot of **PIL1TFSI**.

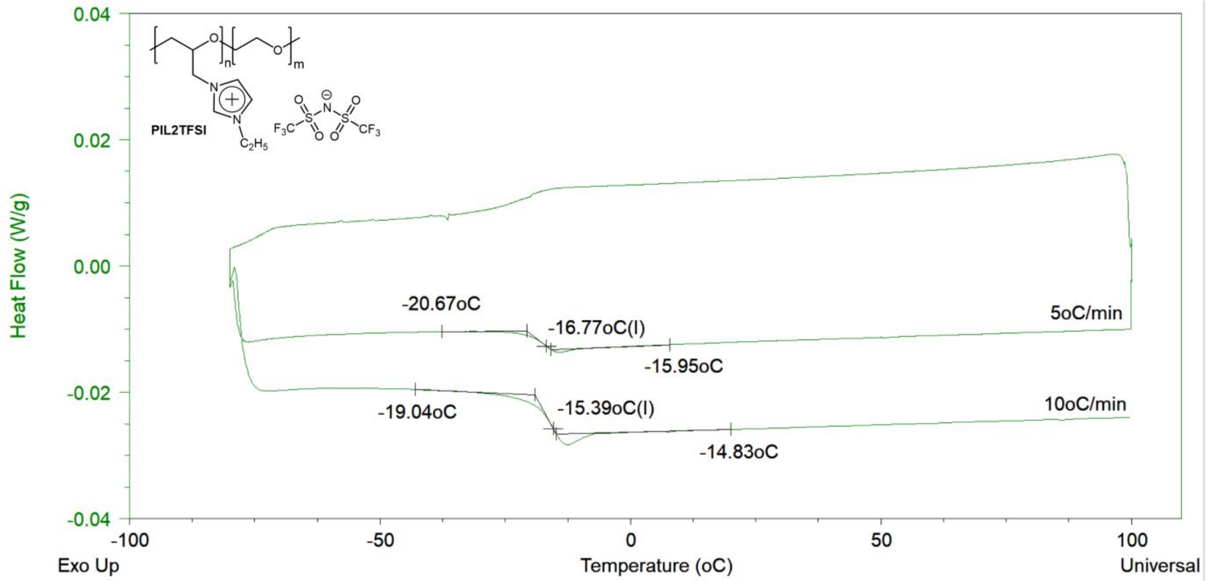

Figure S14. DSC plot of **PIL2TFSI**.

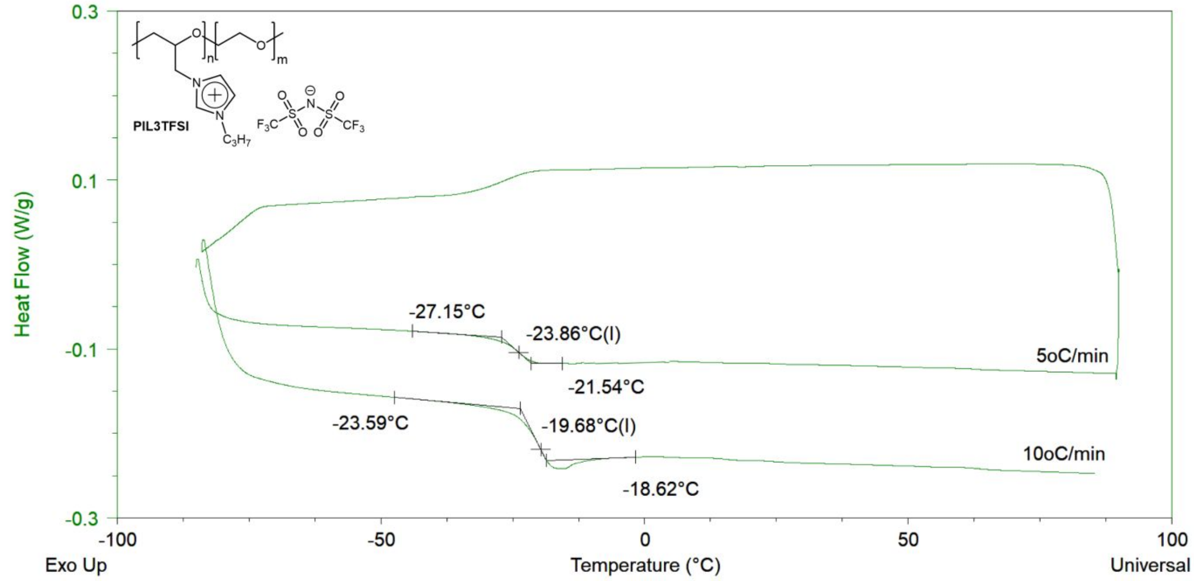

Figure S15. DSC plot of **PIL3TFSI**.

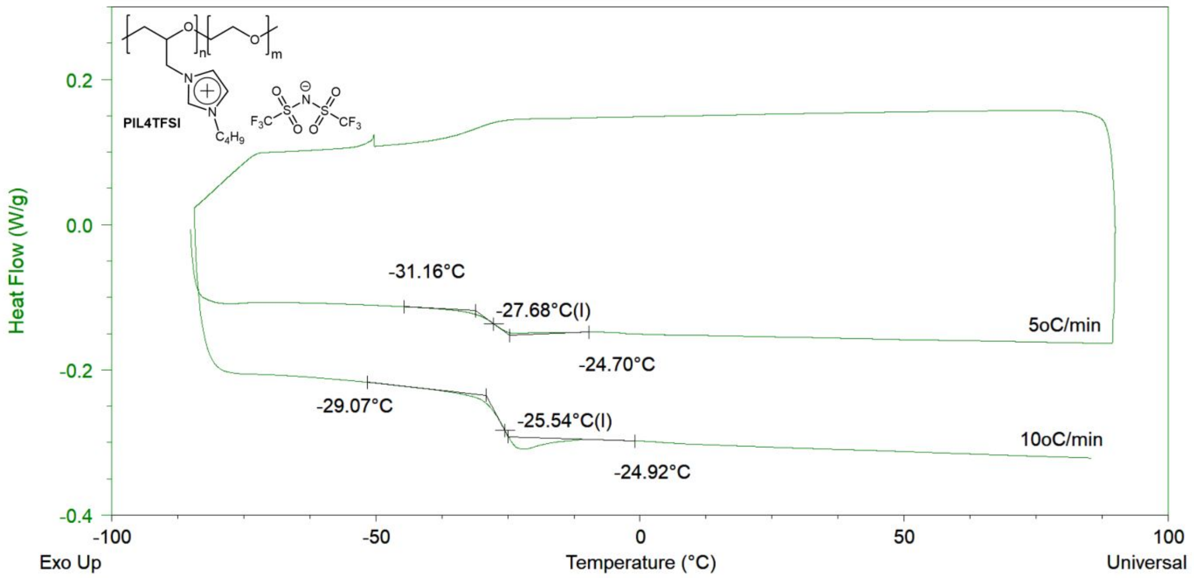

Figure S16. DSC plot of **PIL4TFSI**.

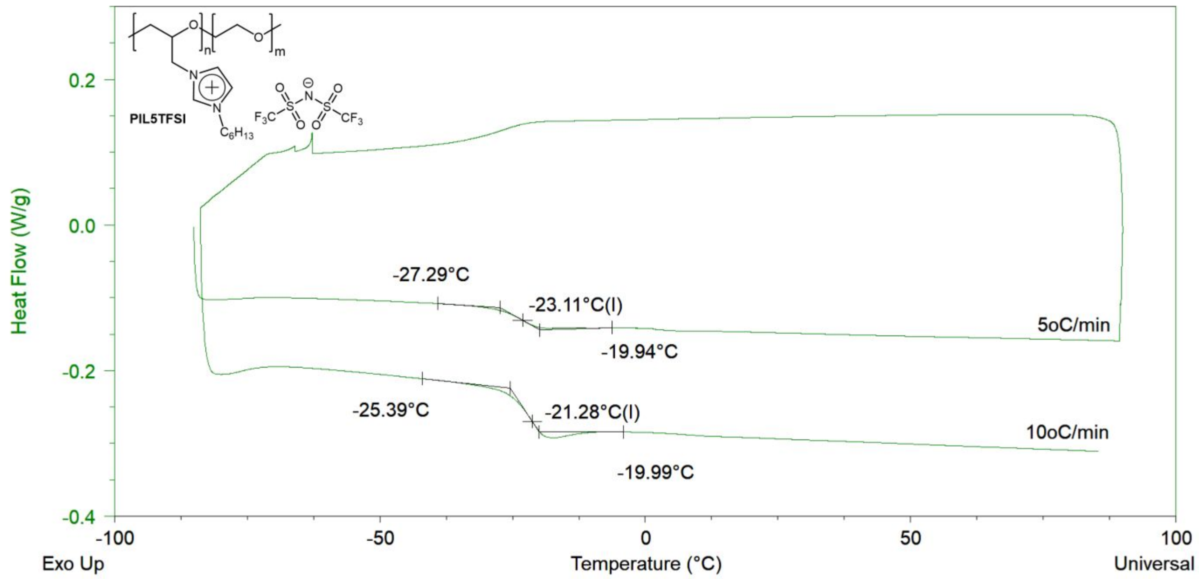

Figure S17. DSC plot of **PIL5TFSI**.

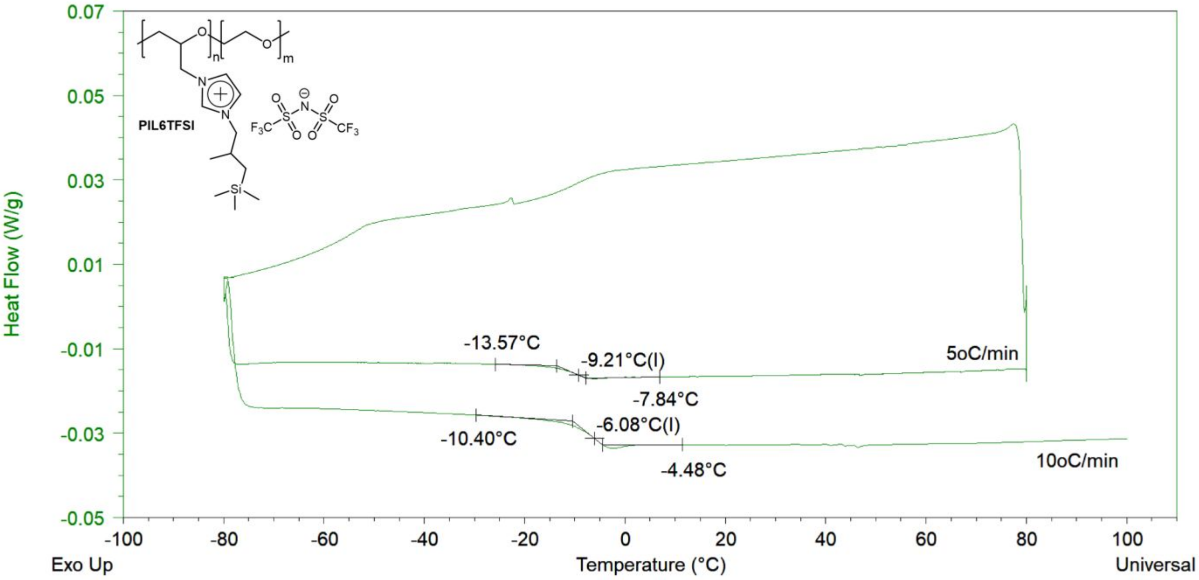

Figure S18. DSC plot of **PIL6TFSI**.

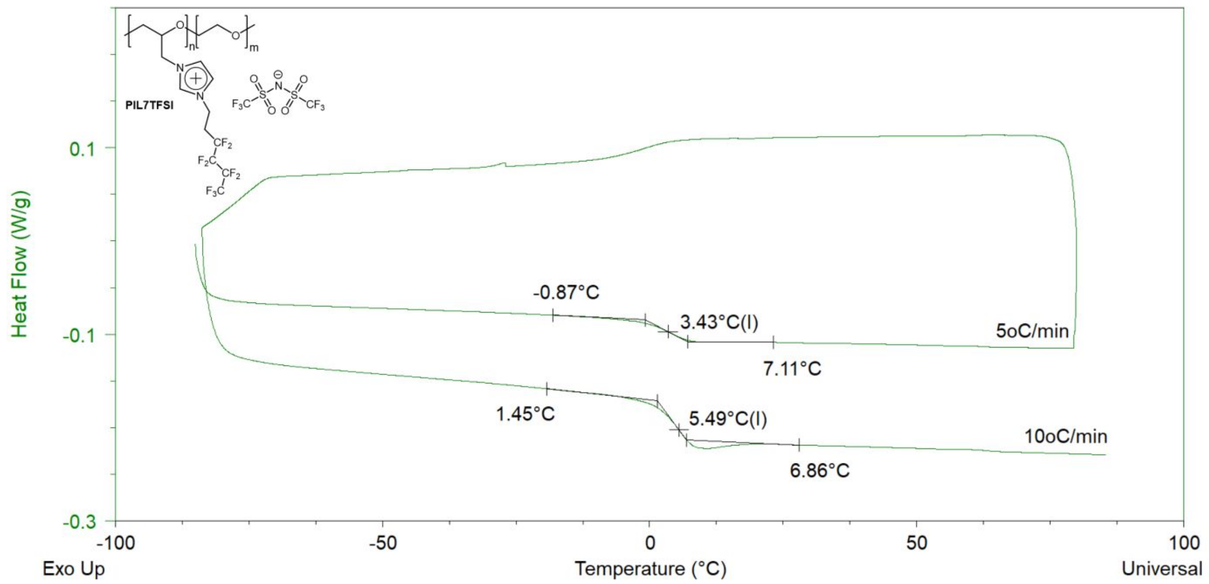

Figure S19. DSC plot of **PIL7TFSI**.

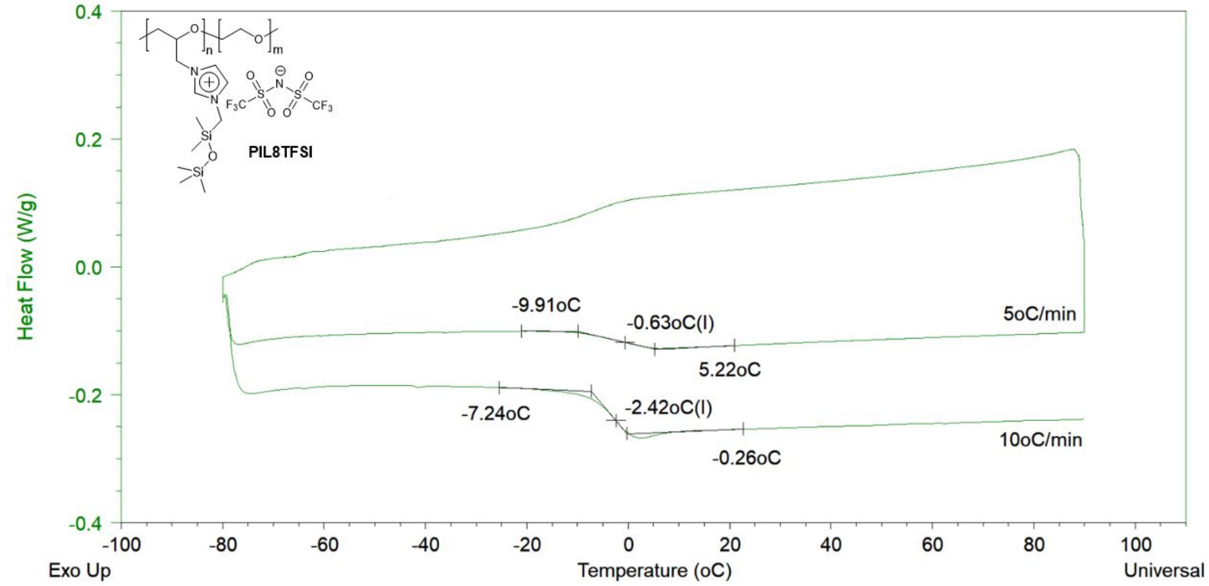

Figure S20. DSC plot of **PIL8TFSI**.

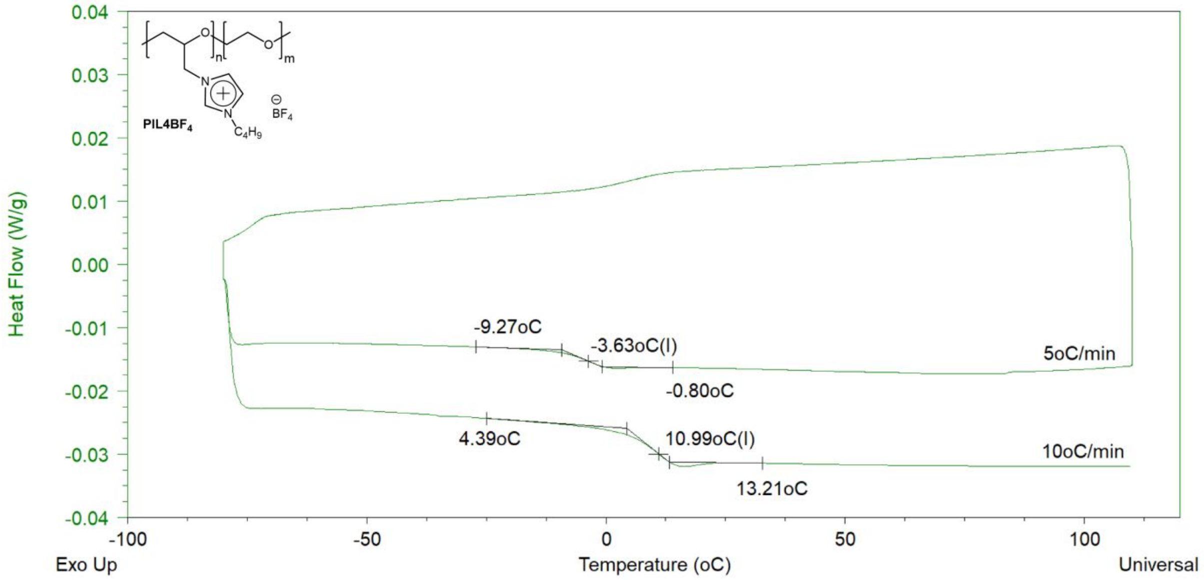

Figure S21. DSC plot of **PIL4BF$_4$**.

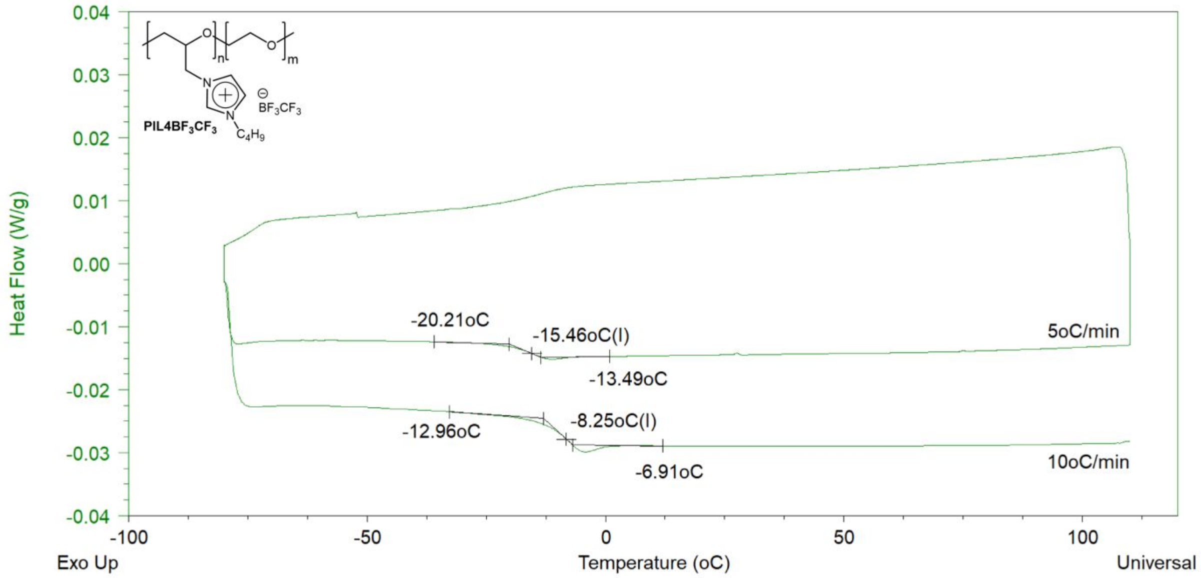

Figure S22. DSC plot of **PIL4BF$_3$CF$_3$**.

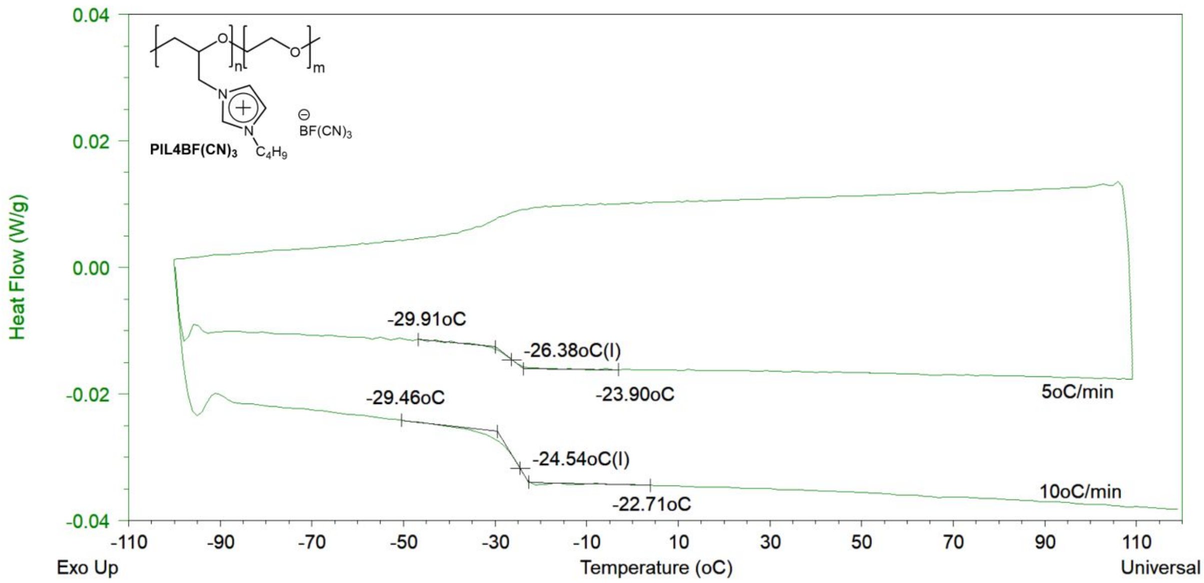

Figure S23. DSC plot of **PIL4BF(CN)$_3$**.

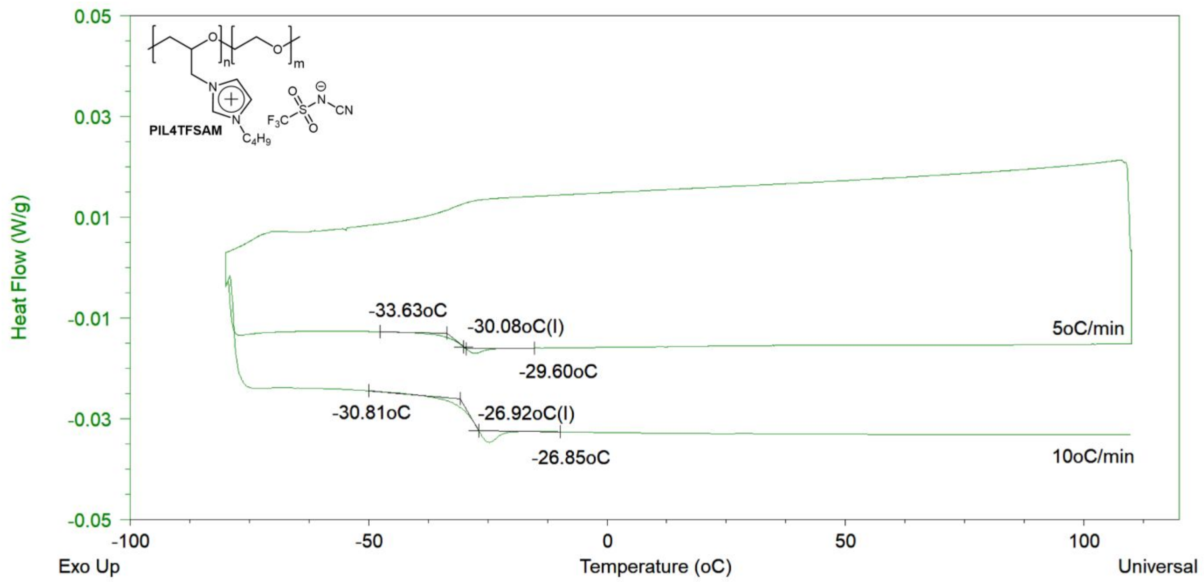

Figure S24. DSC plot of **PIL4TFSAM**.

VII. Representative WAXD plots for qualitative analysis

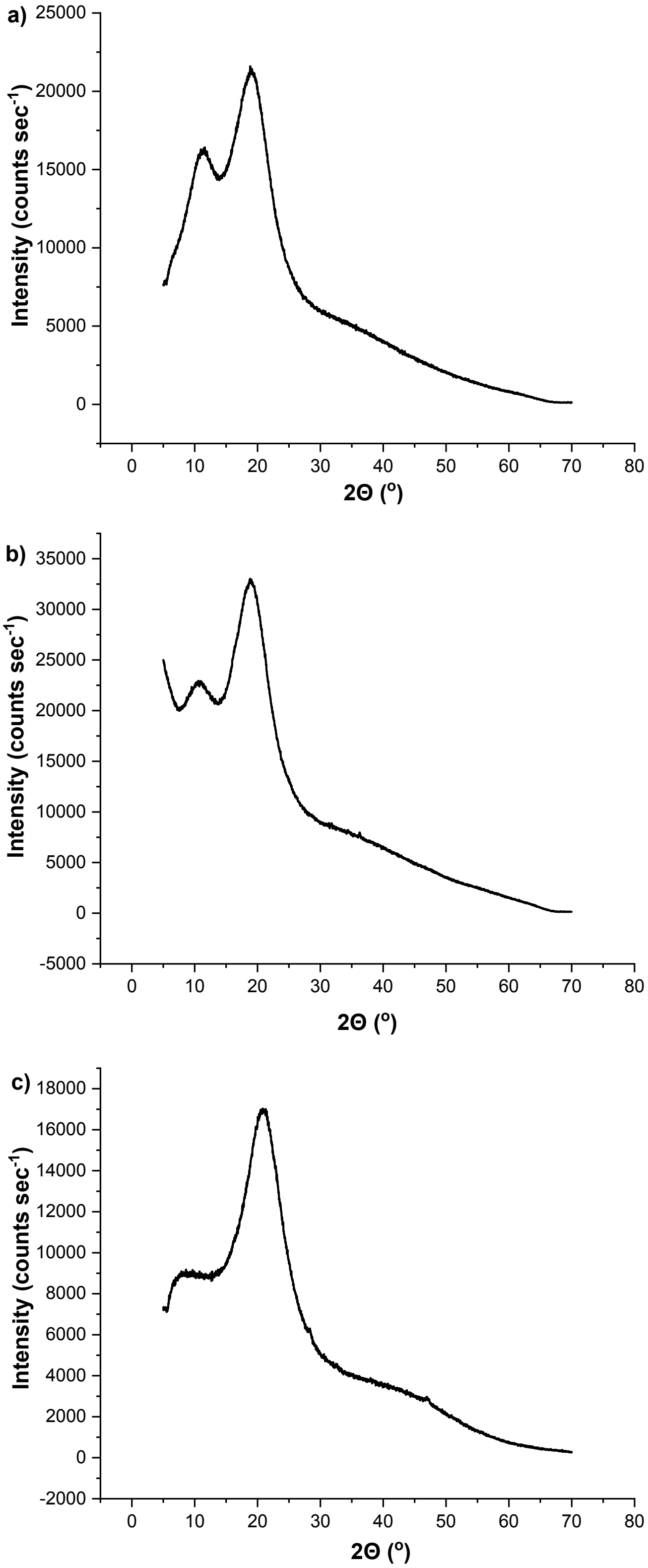

Figure S25. WAXD plots of **PIL4TFSI** (a), **PIL5TFSI** (b) and **PIL4BF$_4$** (c).

VIII. Electrochemical Impedance Spectroscopy (EIS)

Ionic conductivity (σ_{DC}) was measured by electrochemical impedance spectroscopy (EIS or complex alternating current (AC) impedance analysis) with a VSP potentiostat/galvanostat (Bio-Logic Science Instruments, France). To avoid any influence of moisture/ humidity on the conductivity of polymer electrolytes, the latter were preliminary dried at 80 °C/0.1 mbar for 12 h in the B-585 oven (Buchi Glass Drying Oven, Switzerland) filled with P_2O_5 and were transferred under vacuum inside an argon-filled glovebox (MBRAUN MB-Labstar, H_2O and O_2 content<0.5 ppm). The samples were sandwiched between two stainless steel electrodes. The distance between the electrodes was kept equal to 250 μm using a Teflon spacer ring with an inner area of 0.50 cm^2. Symmetrical stainless steel/PIL/stainless steel assembly was clamped into the 2032-coin cell and afterwards was taken out from the glovebox. EIS experiments were carried by applying a 10 mV perturbation in the frequency range from 10^{-2} to 2×10^5 Hz. The measurements were carried out in the temperature range from 20 to 100°C. The temperature was controlled using a programmed M-53 oven (Binder, Germany), where cells were allowed to reach thermal equilibrium for at least 45 minutes before each test.

The ohmic resistance (R_Ω) of the sample, obtained by linear fit from the Nyquist plot (Figure S26) at the low frequency end of the semicircle, was used to calculate the ionic conductivity using the following equation:

$$\sigma = \sigma_{AC} = \frac{l}{S}\frac{1}{R_\Omega} \qquad \text{(eq. S5)}$$

where l is the sample thickness and S is the electrode area.

The determined value of the AC conductivity for **PIL4TFSI** was $\sigma_{AC} = 4.74\times10^{-6}$ S/cm at 25°C.

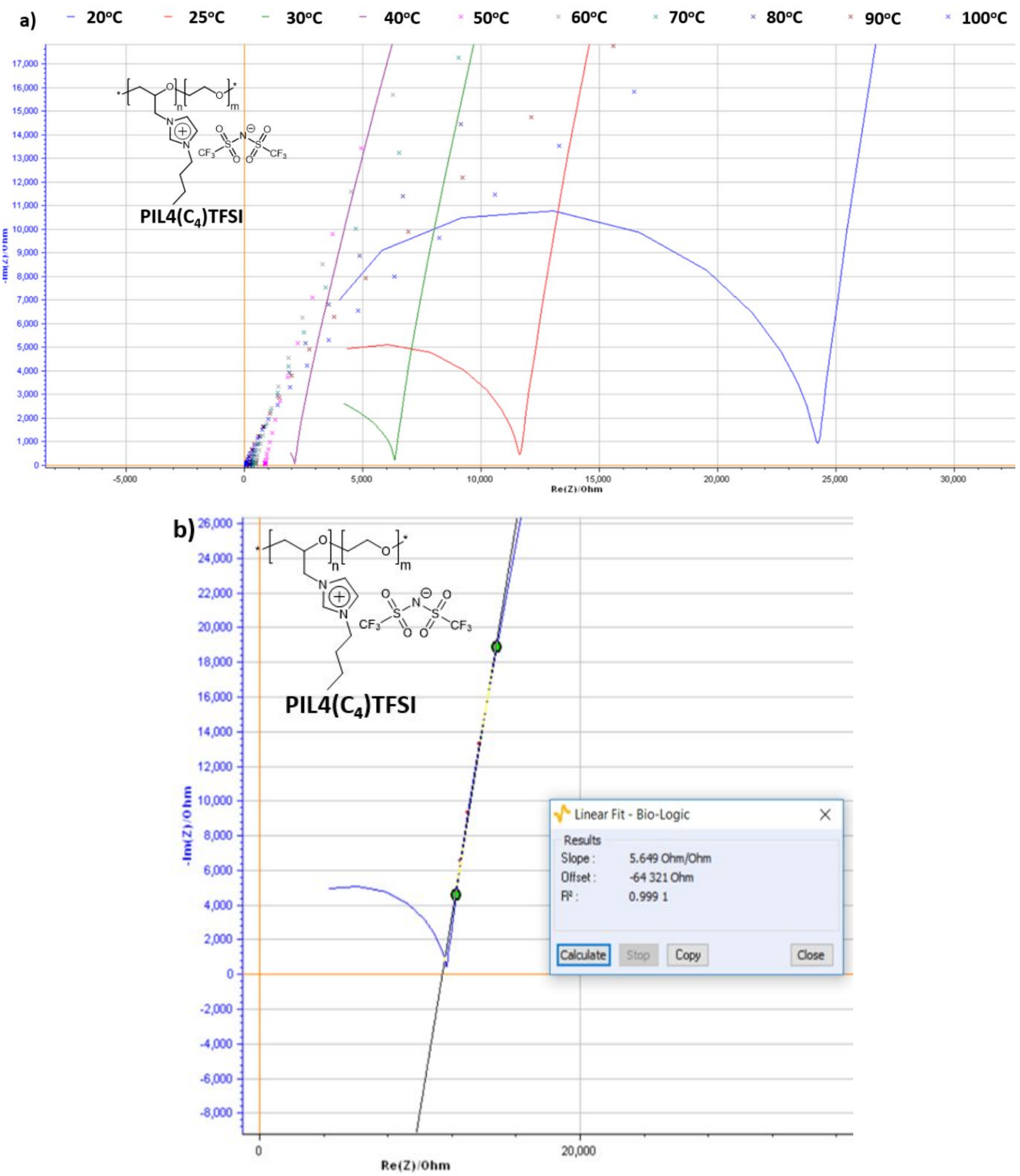

Figure S26. Nyquist plots for **PIL4(C4)TFSI** in the range of temperatures between 20 °C and 100 °C (a) and the Nyquist plot for **PIL4(C4)TFSI** at 25°C with linear fitting (b).

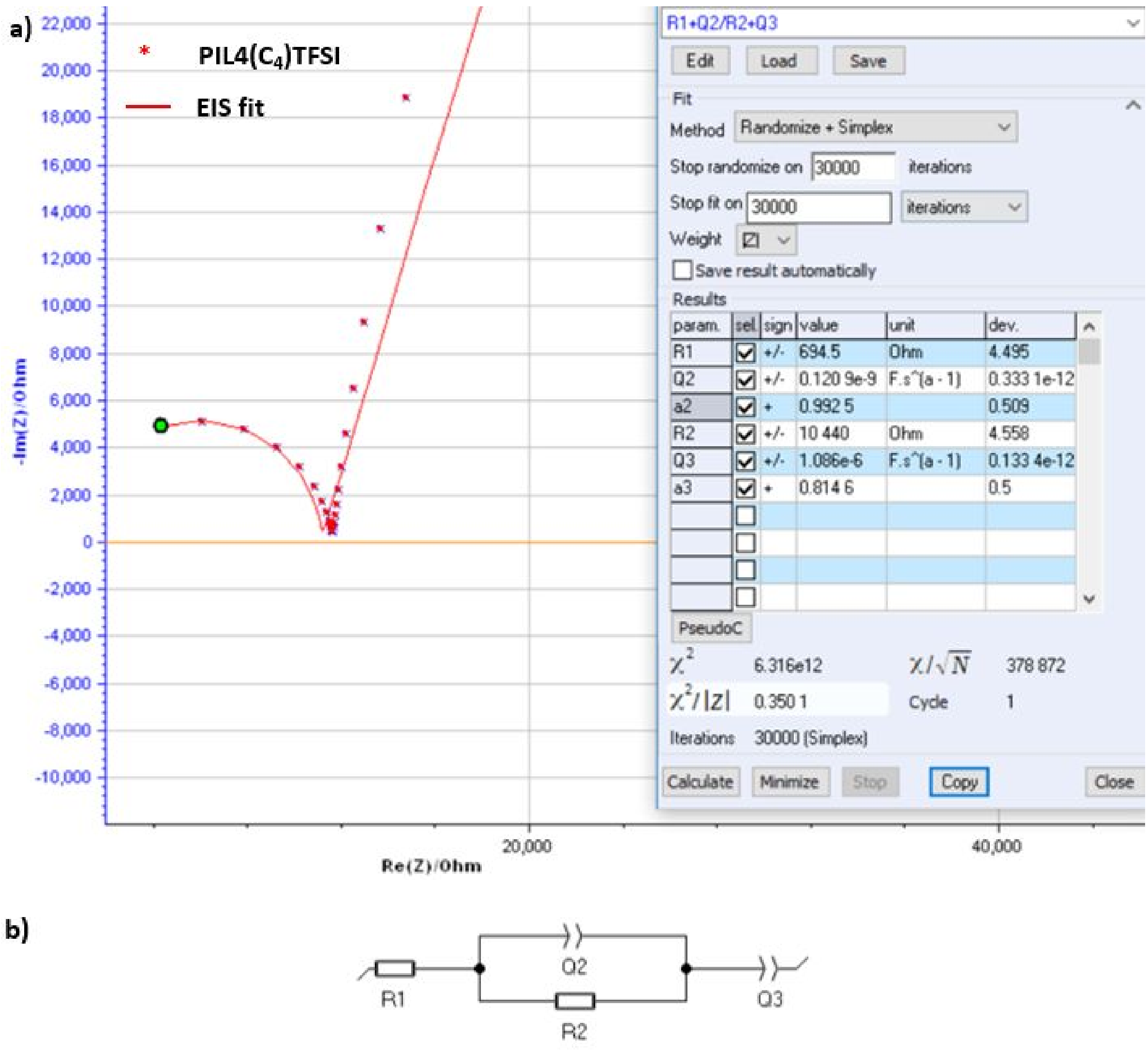

Figure S27. Nyquist plot for **PIL4(C4)TFSI** at 25°C with Z fitting (a) and the equivalent circuit model employed to generate the simulation data in the Nyquist plot for the **PIL4(C4)TFSI** (b).

The Nyquist plot of **PIL4(C4)TFSI** with equivalent circuit fitting is presented in Figure S27a, while the equivalent circuit model is depicted in Figure S27b. It is composed of an electrolyte resistance (R_1), and internal resistance (R_2). The two other elements of the circuit model employed are represented by constant phase elements (Q_2 and Q_3, respectively).

The inverse temperature dependence of σ_{DC} follows a typical Vogel–Fulcher–Tammann (VFT) behavior for all studied PILs. The experimental results were thus fitted with the VFT equation S6:

$$\sigma_{DC} = \sigma_\infty \times exp(-\frac{B}{(T-T_{VFT})}) \qquad \text{(eq S6)}$$

where σ_∞ is the ionic conductivity in the limit of high temperatures, B is the fitting parameter related to the activation energy of the ionic conduction, and T_{VFT} is the Vogel temperature. The obtained parameters for all PILs are listed in Table S2.

Table S2. Parameters obtained by VFT fits of the EIS data.

sample	σ_∞, S cm^{-1}	*B*, *K*	T_{VFT}, *K*	$T_g - T_{\mathrm{VFT}}$, *K*	E_a, *kJ/mol*[a]
**PIL1TFSI**	0.89	992	226	37	88.6
**PIL2TFSI**	0.56	1010	213	45	72.3
**PIL3TFSI**	1.62	1325	198	55	72.4
**PIL4TFSI**	1.47	1228	200	48	70.0
**PIL5TFSI**	0.43	995	215	37	70.9
**PIL6TFSI**	0.46	1133	220	47	87.5
**PIL7TFSI**	4.02	1926	197	82	101.4
**PIL8TFSI**	0.57	1193	218	53	93.9
**PIL4BF$_4$**	4.33	1957	197	72	106.6
**PIL4TFSAM**	0.12	802	220	23	64.8
**PIL4BF(CN)$_3$**	3.80	1392	189	53	66.9
**PIL4BF$_3$CF$_3$**	1.31	1459	201	57	83.9

[a] Activation energy was determined from the slope of a best linear fit to the VFT data.

IX. Broadband dielectric spectroscopy (BDS)

Dielectric spectroscopy studies were performed on a Novocontrol Broadband Dielectric Spectrometer equipped with an Alpha analyzer and a Quatro temperature controller. **PIL4TFSI** was sandwiched between the gold-coated brass electrodes. The experiments were carried out isothermally increasing the temperature from 20 to 50° C in 10°C steps in the 10^{-1}-10^{7} Hz frequency range. Using the Novocontrol Broadband Dielectric Spectrometer the complex impedance Z*(ω) of a PIL at angular frequency ω of the applied electric field is directly measured. The conductivity spectrum is calculated using the equation S7:

$$\sigma * = \sigma_{DC} = \sigma' - i\sigma'' = \frac{1}{Z*(\omega)}\frac{l}{S} \quad \text{(eq. S7)}$$

where C_0 is the capacity of the empty sample capacitor, l is the sample thickness, and S is the electrode area.

The value of the DC conductivity was obtained by thorough analysis of the conductivity spectrum and the dipolar contribution. The DC conductivity was taken from the plateau region (Figure S28a) at an angular frequency corresponding to the maximum of dielectric loss tangent (Figure S28b). The determined value of the DC conductivity for **PIL4TFSI** was σ_{DC} = 4.81×10^{-6} S/cm at 25°C.

While DC conductivity refers to the conductivity of a material when a constant or direct current (DC) is applied, the AC conductivity refers to the conductivity when an alternating current (AC) is applied, which changes direction periodically. DC conductivity focuses on direct current and involves a constant electric field. AC conductivity deals with alternating current and involves an oscillating electric field. EIS method is usually measuring the AC conductivity, while BDS is providing the estimation of the DC conductivity. Whereas most studies on PILs use exclusively one of these complementary analytical techniques, we already have compared the values obtained by both techniques under identical preparation conditions in our previous publication[2]. It was revealed that the conductivity σ values for PILs obtained from BDS and AC impedance techniques are perfectly overlapping[2]. This means that in the studied ranges of temperatures and ionic conductivities the utilization of both techniques is identically reliable and, in both cases, provides the values of σ_{DC}.

Similarly to the previous report[2] in the current work the conductivity values for **PIL4TFSI** obtained by EIS technique and BDS method were identical (4.74×10^{-6} and 4.81×10^{-6}S/cm at 25°C, respectively).

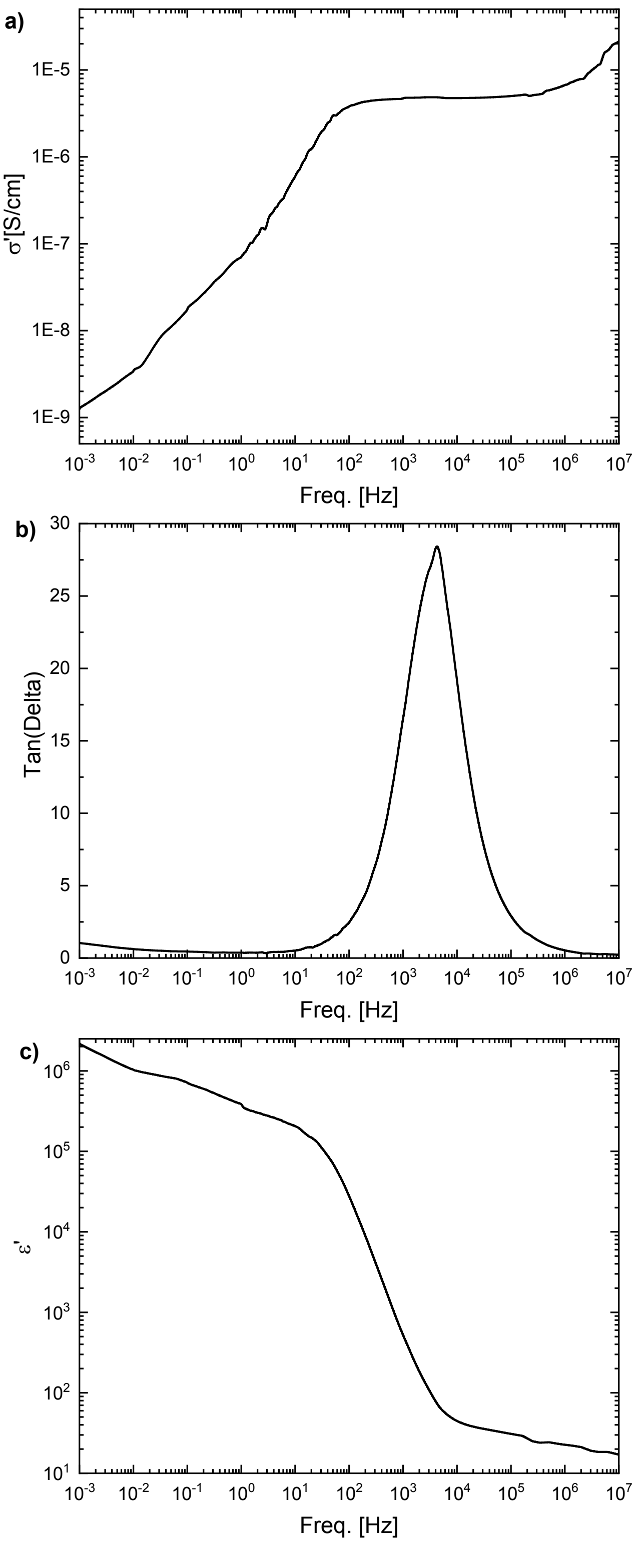

Figure S28. Ionic conductivity as a function of frequency (a), dielectric loss tangent as a function of frequency (b), dielectric constant as a function of frequency (c) measured by BDS at 25 °C for **PIL4TFSI**.

X. Cyclic voltammetry (CV) of PIL4TFSI vs. Li/Li^+ at 70 °C

The sample of **PIL4TFSI/LiTFSI** was prepared as follows: LiTFSI (0.055 g, 0.19 mmol, 10 wt%) was dissolved in 5 ml of anhydrous MeCN. This solution was then added to the solution of **PIL4TFSI** (0.500 g, 0.99 mmol, 90 wt%) in 5 ml of MeCN. After stirring for 1 h at 25°C MeCN was evaporated under reduced pressure, and the resulting sticky product was dried at 70 °C /0.1 mbar for 2 days in B-585 oven (Buchi Glass Drying Oven, Switzerland) filled with P_2O_5. Afterwards it was transferred under vacuum into the argon filled glove box (MBRAUN MB-Labstar, H_2O and O_2 content < 0.5 ppm) and was stored for 5 days prior to further investigation.

CV was used to determine the electrochemical stability window (ESW) of **PIL4TFSI/LiTFSI** at 70 °C. VMP3 multipotentiostat (20 V, ±400 mA, Bio-Logic Science Instruments) and ECC-Std test cells (EL-Cell GmbH, Germany) were used to carry out the electrochemical characterization. Moisture contaminations were avoided by assembling the cells inside the Ar-filled glove-box (MBRAUN MB-Labstar, H_2O and O_2 content < 0.5 ppm). The two-electrode cells were assembled by sandwiching copolymers between working electrode and a lithium metal foil, which served as the reference and the counter electrode, simultaneously. Separate tests were performed for the determination of the cathodic and anodic electrochemical stability. Stainless steel and copper disks were used as working electrodes during anodic and cathodic scans, respectively. To evaluate anodic limits, potential sweeps were carried out between OCV and 5.5 V vs. Li^+/Li at a constant rate of 2.0 mV s^{-1}. To determine cathodic limits, potential sweeps were performed between OCV and -0.4 V vs. Li^+/Li at the same constant rate.

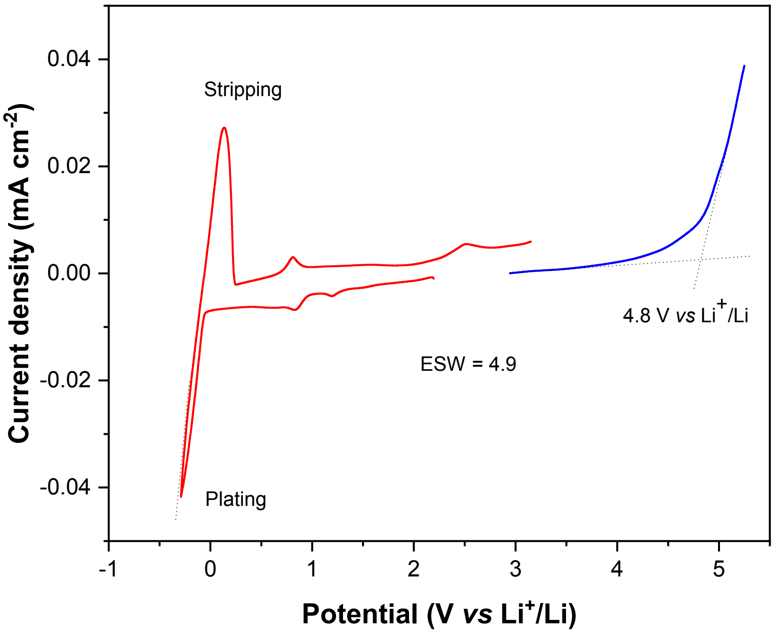

Figure S29. CV diagram of the **PIL4TFSI** with 10 wt% of LiTFSI vs. Li^+/Li at 70°C, scan rate of 2 mV s^{-1}

Table S3. Comparison of the ionic conductivity data for the selected PILs reported in literature and for those obtained in the present work (updated on May, 2024).

No	PIL	σ_{DC}[a], S cm^{-1}	*T*[b], °C	Mn[c], kDa	DP[c]	Reference
1	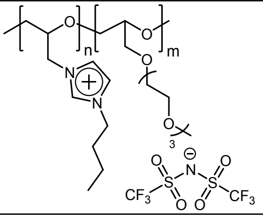	1.2×10^{-4}	25	11.8[d]	35	3
2	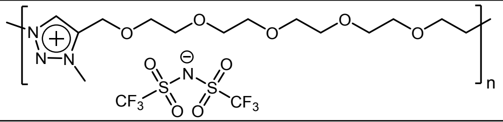	1.2×10^{-4}	30	16.2[d]	27	4
3	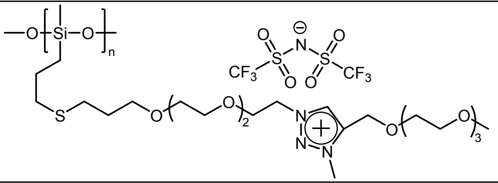	6.7×10^{-5}	30	30.2	36	5
4	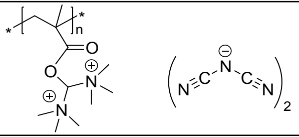	5.5×10^{-5}	25	1830 [d]	-	6
5	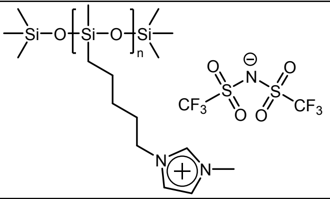	5.0×10^{-5}	25	18.5[d]	37	7
6	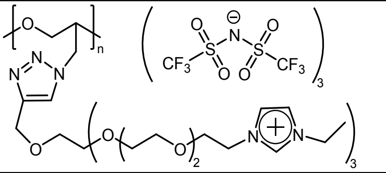	3.6×10^{-5}	25	1900.0[d]	1100	8
7	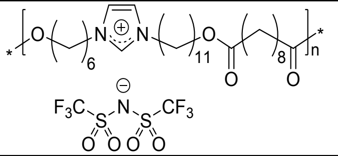	3.2×10^{-5}	25	26.0	43	9
8	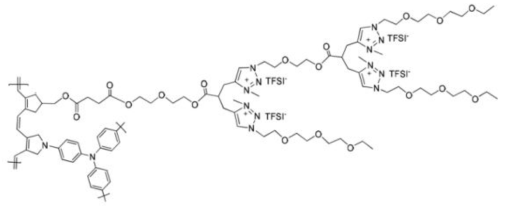	2.9×10^{-5}	30	23.0	9	10
9	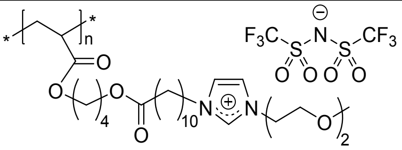	2.8×10^{-5}	25	6.7	8	11

10	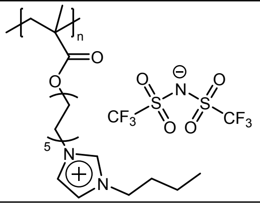	2.5×10^{-5}	30	47.1	75	12
11	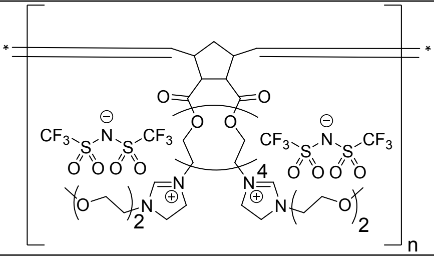	2.3×10^{-5}	25	45.0	27	13
12	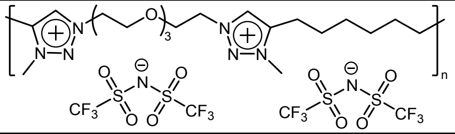	1.8×10^{-5}	25	71.7	72	14
13	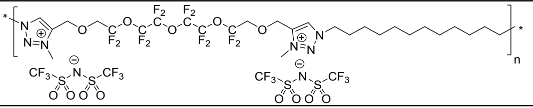	1.2×10^{-5}	30	-	-	15
14	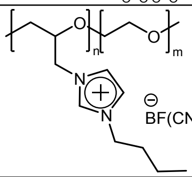	1.0×10^{-5}	25	8730.0[e]	92000	**This work**
15	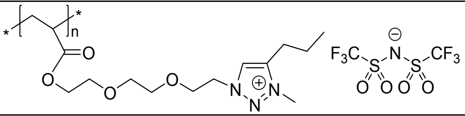	1.0×10^{-5}	30	113.6	182	16
16	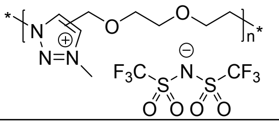	1.0×10^{-5}	25	44.0	86	2

[a] DC conductivity under anhydrous conditions;
[b] Temperature at which σ_{DC} was measured;
[c] Number-average molecular weight and calculated degree of polymerization;
[d] From a neutral precursor polymer;
[e] Mw of a neutral precursor polymer;

SUPPLEMENTARY REFERENCES

(1) Menges, F. Spectragryph-Optical Spectroscopy Software, 1.2.15. 2020. Available Online: Http://Www.Effemm2.de/Spectragryph (Accessed on 12 May 2021).

(2) Colliat-Dangus, G.; Obadia, M. M.; Vygodskii, Y. S.; Serghei, A.; Shaplov, A. S.; Drockenmuller, E. Unconventional Poly(Ionic Liquid)s Combining Motionless Main Chain 1,2,3-Triazolium Cations and High Ionic Conductivity. *Polym Chem* **2015**, *6* (23), 4299–4308. https://doi.org/10.1039/C5PY00526D.

(3) Hu, H.; Yuan, W.; Jia, Z.; Baker, G. L. Ionic Liquid-Based Random Copolymers: A New Type of Polymer Electrolyte with Low Glass Transition Temperature. *RSC Adv* **2015**, *5* (5), 3135–3140. https://doi.org/10.1039/c4ra13432j.

(4) Puguan, J. M. C.; Boton, L. B.; Kim, H. Triazole-Based Ionene Exhibiting Tunable Structure and Ionic Conductivity Obtained via Cycloaddition Reaction: A New Polyelectrolyte for Electrochromic Devices. *Solar Energy Materials and Solar Cells* **2018**, *188* (June), 210–218. https://doi.org/10.1016/j.solmat.2018.09.009.

(5) Jourdain, A.; Serghei, A.; Drockenmuller, E. Enhanced Ionic Conductivity of a 1,2,3-Triazolium-Based Poly(Siloxane Ionic Liquid) Homopolymer. *ACS Macro Lett* **2016**, *5* (11), 1283–1286. https://doi.org/10.1021/acsmacrolett.6b00761.

(6) Shaplov, A. S.; Lozinskaya, E. I.; Losada, R.; Wandrey, C.; Zdvizhkov, A. T.; Korlyukov, A. A.; Lyssenko, K. A.; Malyshkina, I. A.; Vygodskii, Y. S. Polymerization of the New Double-Charged Monomer Bis-1,3(N,N,N-Trimethylammonium Dicyanamide)-2-Propylmethacrylate and Ionic Conductivity of the Novel Polyelectrolytes. *Polym Adv Technol* **2011**, *22* (4), 448–457. https://doi.org/10.1002/PAT.1569.

(7) Wojnarowska, Z.; Feng, H.; Fu, Y.; Cheng, S.; Carroll, B.; Kumar, R.; Novikov, V. N.; Kisliuk, A. M.; Saito, T.; Kang, N. G.; Mays, J. W.; Sokolov, A. P.; Bocharova, V. Effect of Chain Rigidity on the Decoupling of Ion Motion from Segmental Relaxation in Polymerized Ionic Liquids: Ambient and Elevated Pressure Studies. *Macromolecules* **2017**, *50* (17), 6710–6721. https://doi.org/10.1021/acs.macromol.7b01217.

(8) Ikeda, T. Poly(Ionic Liquid)s with Branched Side Chains: Polymer Design for Breaking the Conventional Record of Ionic Conductivity. *Polym Chem* **2021**, *12* (5), 711–718. https://doi.org/10.1039/d0py01333a.

(9) Lee, M.; Choi, U. H.; Salas-De La Cruz, D.; Mittal, A.; Winey, K. I.; Colby, R. H.; Gibson, H. W. Imidazolium Polyesters: Structure-Property Relationships in Thermal Behavior, Ionic Conductivity, and Morphology. *Adv Funct Mater* **2011**, *21* (4), 708–717. https://doi.org/10.1002/ADFM.201001878.

(10) Li, H.; Zhang, H.; Liao, X.; Sun, R.; Xie, M. Incorporating Trifunctional 1,6-Heptadiyne Moiety into Polyacetylene Ionomer for Improving Its Physical and Conductive Properties. *Polym Chem* **2020**, *11* (19), 3322–3331. https://doi.org/10.1039/d0py00109k.

(11) Lee, M.; Choi, U. H.; Colby, R. H.; Gibson, H. W. Ion Conduction in Imidazolium Acrylate Ionic Liquids and Their Polymers. *Chemistry of Materials* **2010**, *22* (21), 5814–5822. https://doi.org/10.1021/CM101407D.

(12) Chen, M.; Dugger, J. W.; Li, X.; Wang, Y.; Kumar, R.; Meek, K. M.; Uhrig, D. W.; Browning, J. F.; Madsen, L. A.; Long, T. E.; Lokitz, B. S. Polymerized Ionic Liquids: Effects of Counter-Anions on Ion Conduction and Polymerization Kinetics. *J Polym Sci A Polym Chem* **2018**, *56* (13), 1346–1357. https://doi.org/10.1002/pola.29015.

(13) Price, T. L.; Choi, U. H.; Schoonover, D. V.; Wang, D.; Heflin, J. R.; Xie, R.; Colby, R. H.; Gibson, H. W. Studies of Ion Conductance in Polymers Derived from Norbornene Imidazolium Salts Containing Ethyleneoxy Moieties. *Macromolecules* **2019**, *52* (4), 1389–1399. https://doi.org/10.1021/acs.macromol.8b02303.
(14) Cotessat, M.; Flachard, D.; Nosov, D.; Lozinskaya, E. I.; Ponkratov, D. O.; Schmidt, D. F.; Drockenmuller, E.; Shaplov, A. S. Effects of Repeat Unit Charge Density on the Physical and Electrochemical Properties of Novel Heterocationic Poly(Ionic Liquid)s. *New Journal of Chemistry*. **2021**, pp 53–65. https://doi.org/10.1039/d0nj04143b.
(15) Anaya, O.; Kallel Elloumi, A.; Thankappan, H.; Abdelhedi Miladi, I.; Serghei, A.; Ben Romdhane, H.; Drockenmuller, E. Synthesis and Structure/Properties Correlations of Fluorinated Poly(1,2,3-Triazolium)s. *Chemistry Africa* **2020**, *3* (3), 759–768. https://doi.org/10.1007/s42250-020-00164-1.
(16) Sood, R.; Zhang, B.; Serghei, A.; Bernard, J.; Drockenmuller, E. Triethylene Glycol-Based Poly(1,2,3-Triazolium Acrylate)s with Enhanced Ionic Conductivity. *Polym Chem* **2015**, *6* (18), 3521–3528. https://doi.org/10.1039/C5PY00273G.
